# The DCMU Herbicide Shapes T-cell Functions By Modulating Micro-RNA Expression Profiles

**DOI:** 10.3389/fimmu.2022.925241

**Published:** 2022-07-28

**Authors:** Pierre Autin, Sophie Deshayes, Juliette Lea, Nicolas Boisgerault, Emilie Dupré, Nathalie Labarrière, Rémy Leguevel, Jean-François Fonteneau, Christophe Blanquart, Delphine Fradin

**Affiliations:** ^1^ Nantes Université, INSERM UMR1307, CNRS UMR6075, Université d’Angers, CRCI2NA, Nantes, France; ^2^ Université de Rennes, ImPACcell Plateform, BIOSIT, Rennes, France; ^3^ Nantes Université, Univ Angers, INSERM, CNRS, Immunology and New Concepts in ImmunoTherapy, INCIT, UMR 1302/EMR6001, Nantes, France

**Keywords:** diuron, herbicide, CD8+ T cells, micro-RNA, cancer

## Abstract

DCMU [N-(3,4-dichlorophenyl)-N-dimethylurea] or diuron is a widely used herbicide, which can cause adverse effects on human, especially on immune cells, due to their intrinsic properties and wide distribution. These cells are important for fighting not only against virus or bacteria but also against neoplastic cell development. We developed an approach that combines functional studies and miRNA and RNA sequencing data to evaluate the effects of DCMU on the human immune response against cancer, particularly the one carried out by CD8+ T cells. We found that DCMU modulates the expression of miRNA in a dose-dependent manner, leading to a specific pattern of gene expression and consequently to a diminished cytokine and granzyme B secretions. Using mimics or anti-miRs, we identified several miRNA, such as hsa-miR-3135b and hsa-miR-21-5p, that regulate these secretions. All these changes reduce the CD8+ T cells’ cytotoxic activity directed against cancer cells, *in vitro* and *in vivo* in a zebrafish model. To conclude, our study suggests that DCMU reduces T-cell abilities, participating thus to the establishment of an environment conducive to cancer development.

## Introduction

CD8+ T cells are the most powerful effectors in the anti-tumor immune response. They robustly proliferate upon priming and activation, acquire effector functions, and migrate to the site of interest to eliminate tumor cells, through the exocytosis of perforin- and granzyme-containing granules. Moreover, CD8+ T cells secrete high amount of cytokines, such as interleukin (IL)-2, interferon-gamma (IFN-γ) and tumor necrosis factor alpha (TNF-α) to promote the anti-tumoral activity of all immune cells. Deployment of these functions in CD8+ T cells is accompanied by changes in their gene expression profiles. While many of these changes occur at the transcriptional level, as much as 50% are mediated post-transcriptionally (1), including by non-coding RNA such as micro-RNAs (miRNAs) (2). miRNAs are endogenous small non-coding RNA about 19–22 nucleotides that modulate gene expression through translational repression or degradation of target messenger RNA (mRNAs) (3). A single miRNA can target hundreds of mRNA in humans (4), and more than 60% of protein-coding genes are under the control of miRNA (5). This explains how aberrant expression of a small number of miRNA can dramatically alter CD8+ T-cell functions.

Diuron, or DCMU [N-(3,4-dichlorophenyl)-N-dimethylurea], is a photosystem II inhibitor used in agricultural activities and antifouling paints in the shipping sector. Its regulation varies across countries or uses. DCMU was, for example, banished in France since 2002 but still authorized elsewhere in Europe, North America, or Africa. It is widely spread over sugarcane, citrus fruit, banana, coffee, and cotton plantations (6, 7). According to the European Food Safety Authority, acceptable daily intake (ADI) of DCMU is 0.007 mg/kg bw/day, whereas acute reference dose (ARfD) is 0.016 mg/kg bw/day. Occupational exposure is one of the main concerns about this herbicide, mainly through pollution of both soil and water (8–12). However, this herbicide is also responsible for a high number of acute intoxications, voluntary or not, which lead to long-term consequences for poisoned persons (13, 14). It presents, therefore, its fair share of toxicity in humans even if they are poorly described (15, 16).

Several omics studies have been conducted to better decipher the relationship between cancer risks and environmental exposures. Transcriptomic studies about small RNAs have shown that the miRNA machinery and patterns are altered in response to environmental pollutants, interestingly before the onset of cancer (17). Most of these miRNA changes have been observed directly in tumor cells or in circulating cells for their use as potential biomarker, but their investigation in immune cells could be of huge interest as well, since immune cells influence greatly each step of tumor development.

We postulate then that DCMU exposure might change miRNA expressions of CD8+ T cells, leading to an altered anti-tumor immune response favoring tumor development and proliferation. Consistent with this, we demonstrated that DCMU exposure induces massive dysregulations of miRNA patterns leading to altered mRNA expressions and pathways involved in the CD8+ T-cell functions. Then, we showed that these dysregulations consequently reduce cytokine secretions and cytotoxic abilities of CD8+ T cells, creating a permissive environment to tumor development as demonstrated in animal model.

## Materials and Methods

### CD8+ T-Cell Generation and Purification

CTL03.1 and N5.14 CD8+ T-cell clones specific for Melan-A and MUC1, respectively, were obtained and cultured as previously described (18, 19). Polyclonal CD8+ T cells were obtained from fresh blood (Etablissement Français du Sang, ethics agreement CPDL-PLER-2018 021). Peripheral blood mononuclear cells were separated using Ficoll gradient (Eurobio, Les Ulis, France; Cat#CMSMSL01-01). Polyclonal CD8+ T cells were then sorted using EasySep Human CD8+ T Cell Isolation Kit (STEMCELL Technologies, Vancouver, Canada; Cat#17953). Purity was assessed after sorting by flow cytometry following a 20-min staining at 4°C with CD3 and CD8 antibodies directly conjugated to fluorescein isothiocyanate (FITC, BD Biosciences, Pont de Claix, France; Cat#555339, RRID : AB_395745) and phycoerythrin (PE, BD Biosciences, Pont de Claix, France; Cat#555367, RRID : AB_395770), respectively. Polyclonal T cells were considered acceptable for further experiments when CD3+CD8+ population among viable cells represented over 90%.

### Cell Culture and Cell Line Generations

T cells (monoclonal or polyclonal) were cultured in Roswell Park Memorial Institute (RPMI) 1640 Medium [Gibco (Invitrogen), Carlsbad, CA, USA] supplemented with 100 U/ml penicillin, 100 mg/ml streptomycin, 2 mM L-glutamine [Gibco (Invitrogen), Carlsbad, CA, USA], 8% human serum (local production), and 150 UI IL-2 (Proleukin^®^, Novartis, Basel, Switzerland), and cultured at 37°C in a 5% CO_2_ atmosphere. Melanoma cell lines were cultured in RPMI 1640 medium [Gibco (Invitrogen), Carlsbad, CA, USA] supplemented with 100 U/ml penicillin, 100mg/ml streptomycin, 2 mM L-glutamine [Gibco (Invitrogen), Carlsbad, CA, USA], 10% fetal bovine serum (FBS) [Hyclone (GE Healthcare), Chicago, IL, USA]. Mesothelioma cell lines were cultured in RPMI 1640 medium [Gibco (Invitrogen), Carlsbad, CA, USA] supplemented with 100 U/ml penicillin, 100 mg/ml streptomycin, 2 mM L-glutamine [Gibco (Invitrogen), Carlsbad, CA, USA], 10% FBS (Corning, Corning, NY, USA). All cells were tested each week to prevent mycoplasma contaminations using PlasmoTest™ (*In vivo*gen, San Diego, CA, USA; Cat#rep-pt1). Briefly, once every week, 500 µl of supernatant and cells from homogenized cell cultures were heated for 15 min at 100°C; then, 50 µl was loaded in 96-well plates together with 50×10^3^ Hek-Blue cells in specific HEK-Blue™ Detection medium that changes color when Hek-Blue cells secrete alkaline phosphatases (e.g., when infected with mycoplasma). Contamination was assessed after a night incubation at 37°C.

Target cell lines used for *in vitro* cytotoxicity assays were modified cell lines. Briefly, retro-viral particles containing NanoLuciferase (NLuc) plasmid were expressed in Lenti-X 293T cell line (plasmid pMX2.1 Nluc). Supernatants from Lenti-X cells were then added twice a day for 4 days on melanoma [M113, M6; PC-U892-NL Biocollection (CHU Nantes, France)], mesothelioma [Méso13, Méso34; DC-2011-1399 Biocollection (CHU Nantes, France)]. Transduced cells were then selected using puromycin at 1 µg/ml for a week. Finally, they were tested for NLuc expression by measuring luminescence released after a 15-min incubation with increasing concentration of Digitonin (Promega, Madison, WI, USA; Cat#G9441).

For *in vivo* cytotoxicity assays, lentiviral particles containing green fluorescent protein (GFP; plasmid pLX CMV GFP from Addgene, Watertown, MA, USA; Cat#17448, RRID : Addgene_17448) plasmid were expressed in Lenti-X 293T cell line. Supernatants from Lenti-X cells were then added twice a day for 3 days on melanoma (M113 and M6) and mesothelioma (Méso13 and Méso34) cell lines. Transduced cells were then selected using puromycin at 1 µg/ml for a week and assessed for GFP expression by microscopy.

Once validated, all these cells were considered as proper target cells usable in cytotoxicity assays and frequently reselected to ensure optimal expression of either NLuc or GFP.

### DCMU Exposure

Powder DCMU was obtained from Sigma-Aldrich, St. Louis, MO, USA (Cat#D2425-100G), and aliquot was made at a final concentration of 100 mM in dimethyl sulfoxide (DMSO) (Sigma-Aldrich, St. Louis, MO, USA). Each aliquot was kept up to 1 month at 4°C. Exposure protocol to DCMU was as follows: cells were numbered and seeded at a concentration of 800×10^3^ cells/ml. DCMU and DMSO solvent controls were thawed at room temperature and used at three different concentrations: 10 µM (2 mg/L), 100 µM (20 mg/L), and 250 µM (50 mg/L). Cell exposure lasted 24 h before cells were washed and used for further experiments.

### DCMU Toxicity Assays

DCMU toxicity on T cells was assessed using CellTiter-Glo^®^ Luminescent Cell Viability Assay (Promega, Madison, WI, USA) according to the manufacturer’s protocol. Briefly, cells were collected following exposure and incubated for 5 min at a 1:1 dilution with CellTiter-Glo^®^ Reagent. Luminescence was measured using a FLUOstar Omega microplate reader (BMG Labtech, Champigny-sur-Marne, France). In CellTiter-Glo^®^ viability assays, the estimation of living cells is based on metabolically active cells by quantifying luminescent signal proportional to the amount of ATP.

### Proliferation Assays

T cells were stained with CellTrace™ carboxyfluorescein succinimidyl ester (CFSE) according to the manufacturer’s protocol (Invitrogen, Carlsbad, CA, USA; Cat#C34554). Cells were then seeded at 100×10^3^ cells/well in a round 96-well plate and stimulated using CD3–CD28 Dynabeads at a 1:100 bead/cells ratio [Gibco (Invitrogen), Carlsbad, CA, USA; Cat#11131D] to induce proliferation. At the same time, cells were exposed, or not, to DCMU or DMSO as previously described during the first 24 h. Following that 24 h exposure, the medium was washed twice with phosphate-buffered saline (PBS) and replaced by standard T cells medium (described in Section 2.2). After 4 more days of proliferation (total, 5 days post-seeding), cells were collected, and CFSE levels were assessed by flow cytometry on a FACSCanto II (BD Biosciences, Pont de Claix, France). Proliferation controls were set as follows: on one side, the negative control was set as AF488 fluorescence level of unstained CD8+ T cells seeded for 5 days with CD3–CD28 beads and with no exposure to DCMU or DMSO. On the other side, positive control was set as fluorescence level of CD8^+^ T cells seeded for 5 days without CD3–CD28 beads and not exposed to DCMU or DMSO. Percent of proliferating cells was then retrieved and normalized compared to equivalent DMSO-exposed conditions for representation.

### ELISA Assays

To quantify cytokine production, supernatants from proliferation assays, described in Section 2.5, were collected at 24 h (before washing of DCMU) and at 5 days (before flow cytometry). Cytokine production were measured in triplicate by ELISA according to the manufacturer’s instructions using uncoated IFN-γ, IL2, TNF-α ELISA kits, and coated granzyme-B kits (Cat# 88-7316-88, 88-7025-88, 88-7346-88, and BMS2027TEN, respectively; Invitrogen, Carlsbad, CA, USA). Absorbance values at 450 nm/570 nm (for IFN-γ, IL2, and TNF-α) or 450 nm/610 nm (for granzyme B) were read using a Multiskan FC microplate reader (Thermo Scientific, Waltham, MA, USA). Quantification was made by subtracting 570 or 610 nm signal to 450 nm one for each well as advised in the manufacturer’s protocol.

### Cytotoxicity Assays

Monoclonal T cells were exposed for 24 h to DCMU, then cocultured with target cells for the appropriate time based on previous experiments (4 h for CTL03.1 vs. melanoma and 24 h for N5.14 vs. mesothelioma). To evaluate specific lysis, CTL03.1 cells were cocultured HLA-A02 melanoma cell line (M113 NLuc) or non-HLA-A02 melanoma cell line (M6 NLuc, HLA-A01) at different T cell/target cell ratios (1:1, 2:1, 5:1, 20:1, and 40:1). The same process was applied for N5.14 clones (against Méso34 NLuc as HLA-A2 cell line and Méso13 NLuc as HLA-A03 cell line). Cytotoxicity was then assessed by measuring luminescence from released NanoLuciferase in culture wells using a FLUOstar Omega microplate reader (BMG LabTech, Champigny-sur-Marne, France).

### miRNA and mRNA Analysis, Target Genes, and Pathways Analysis

Total RNA (including mRNA and miRNA) were extracted using miRNeasy Micro kit (Qiagen, Hilden, Germany; Catalog# 217084). RNA purity and quantification were assessed using Small RNA chips (Agilent Technologies, Santa Clara, CA, USA). Total RNA was then analyzed using Affymetrix miRNA 4.0 chips (GENOM’IC platform, Institut Cochin, Paris, France) as previously described (20). DCMU-dysregulated mature miRNA target genes were selected using results from three tools: miRNAtap and multimir R packages (R3.4.2) and online software mirDIP (21). In details, each tool questioned retrieved a prediction score for specific miRNA–mRNA interaction. At this stage of the analysis, the only inclusion criterion was that the target should be significantly predicted to be the miRNA target by all three tools used (using aggregate mean score retrieved from each tool for each miRNA–mRNA interaction). Common genes were then incremented into R package PathfindR and Enrichr software (RRID : SCR_001575) (22, 23) to visualize if DCMU-dysregulated mature miRNA target genes were enriched significantly in signaling pathways.

For genes analysis, 3′ sequencing RNA profiling was performed by the GenoBird plateform (IRS-UN, Nantes, France) using a NovaSeq 6000 (Illumina Inc., San Diego, CA, USA). The raw sequence reads were filtered based on quality using FastQC (RRID : SCR_014583). Adapter sequences were trimmed off the raw sequence reads using Cutadapt (RRID : SCR_011841). Reads were then aligned to the reference genome using BWA (RRID : SCR_010910). Differential expressions are detected with the DESeq2 Bioconductor package (RRID : SCR_000154 and RRID : SCR_006442) (24). Significantly dysregulated genes were compared to predicted targets obtained for miRNA analysis.

All original microarray and sequencing data were deposited in the NCBI’s Gene-Expression Omnibus database under the references GSE189440 and GSE189443.

### miRNA Transfection

miRNA mimic (Invitrogen, Carlsbad, CA, USA, Cat# 4464066 IDs MH21722, MC21042, MC21694, MH10206, MC29694, and MC23812; and Sigma-Aldrich, St. Louis, MO, USA, Cat# HMI1456, HMI0958, and HMI0119) or antimir (antisense oligonucleotide; Invitrogen, Carlsbad, CA, USA; Cat# 4464084 ID MH10206; and Sigma-Aldrich, St Louis, MO, USA, Cat# HSTUD1456) were transfected into 100.10^3^ CTL03.1 cells that had reached 80% confluence using TransIT-TKO Transfection Reagent (Mirus Bio, Madison, WI, USA; Cat# MIR2150) on a 96-well plate, in triplicates. A miRNA mimic negative control (Invitrogen, Carlsbad, CA, USA; Cat# 4464058) was also transfected and used to set the 100% of secretion. Following 24-h transfection, supernatants were collected to measure cytokine and granzyme B concentrations.

### RT-qPCR

Total RNA was reverse transcribed using RevertAid H Minus Reverse Transcriptase (Thermo Scientific, Waltham, MA, USA), and the RT product was used for expression analysis using Maxima SYBR Green/ROX qPCR Master Mix (Thermo Scientific, Waltham, MA, USA). XCL1 was amplified using the following primers: 5′-TGGCTAGTGTCTATCAGAGGTGA-3′/5′-ATTGTTGCCATTGTCACAGC-3′. RPLP0 (ribosomal protein lateral stalk subunit P0, 5′-GTGATGTGCAGCTGATCAAGACT-3′/5′-GATGACCAGCCCAAAGGAGA-3′) and PPIA (peptidylprolyl isomerase A, 5′-CCCACCGTGTTCTTCGACAT-3′/5′-CCAGTGCTCAGAGCACGAAA-3′) genes were used as reference genes. Each reaction sample was run in duplicate. To circumvent any issue of non-specific amplification, melting curve analysis was performed with a temperature gradient of 70–95°C. The 2^−ΔΔCt^ method was used to calculate relative changes in expression.

### Zebrafish model

Zebrafish were obtained from the Tg (kdrl:Hsa.HRAS-mCherry) strain. Fertilized eggs were incubated at 28°C in an E3 medium and raised under standard condition in the ImPACcell Plateform. To prevent melanization, at 24 h post-fertilization (hpf), 0.2 mmol/L 1-phenyl-2-thio-urea (PTU) was added. At 48 hpf, ~80 GFP-labeled melanoma cells M113 were injected into the duct of Cuvier alone or with ~400 CD8+ T cells that were previously exposed or not to DCMU *in vitro* as described in Section 2.3 (ratio, 1 target cell/5 effector cells). CD8+ T cells were washed twice before injection to remove DCMU. Injected zebrafish larvae were incubated for 4 days in 0.2 mM PTU at 33°C. Tumor growth was monitored each day using a fluorescent microscope Zeiss Observer Z1 (Zeiss, Oberkochen, Germany). Images were analyzed using ImageJ2 (RRID : SCR_003070). The number of green spots per larvae was counted by the find maxima function.

### Statistical Methods

Error bars indicate ± SEM between biological replicates. Unless stated otherwise, technical and biological triplicates of each experiment were performed. Statistical significance was determined using non-parametric Wilcoxon test: NS, non-significant; *p < 0.05; **p < 0.01; *** p < 0.001. All statistical analyses were conducted using R3.4.2.

## Results

### DCMU Toxicity on Human CD8+ T Cells

**Figure 1:**
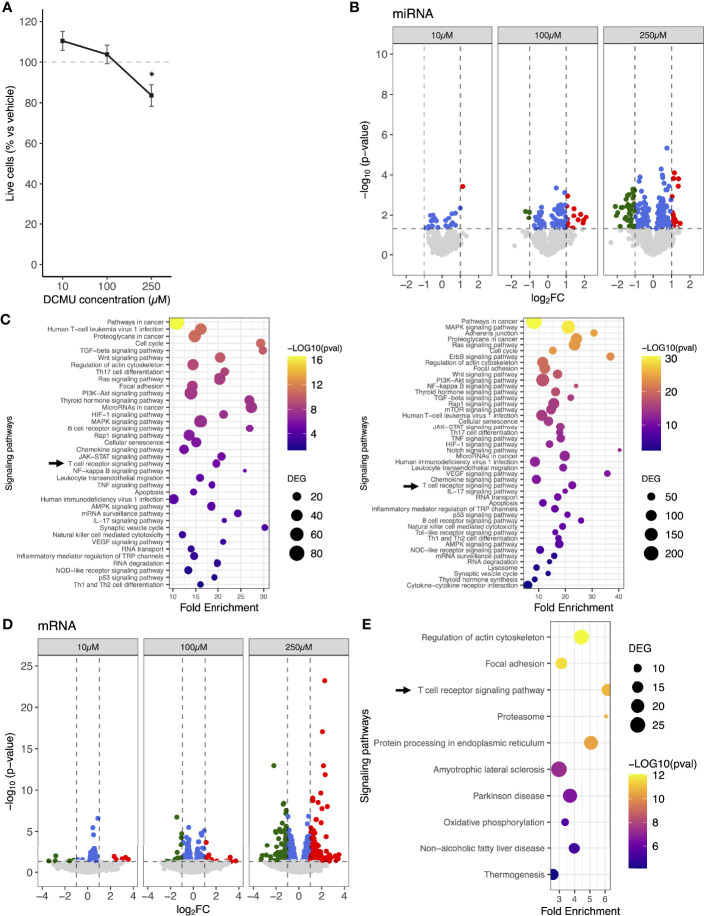
miRNA and mRNA expression changes in human CD8+ T cells following DCMU exposures. **(A)** Cell viability assay of CTL03.1 cells exposed to increasing DCMU concentrations. **(B)** Volcano-plot of the differentially expressed miRNAs. Red: significant upregulation, Green: significant downregulation, Blue: signficant but under the fold-change (FC) cutoff. FC cut off≥1 or ≤-1. **(C)** KEGG pathways enrichment of the DCMU-dysregulated miRNAs at 100µM (left panel) or 250 µM (right panel). **(D)** Volcano-plot of the differentially expressed mRNAs. Red: significant upregulation, Green: significant downregulation, Blue: signficant but under the fold-change (FC) cutoff. FC cutoff≥1 or ≤-1. **(E)** Top 10 of KEGG pathways enrichment of the DCMU-dysregulated mRNAs.

To set DCMU doses used in the following experiments, we first tested toxicity of DCMU in CD8+ T cells by evaluating cell viability in a cancer-specific CD8+ T-cell clone (CTL03.1), directed against the melanoma antigen Melan-A. DCMU concentrations were selected according to two criteria. First is consistency with the literature. We used data from studies investigating blood concentrations of various pesticides following acute intoxications (25, 26). We could not apply such an analysis only on DCMU, since information available on blood concentration is sparse for that herbicide (27). This analysis revealed that blood concentrations are very variable, and the expected correlation between concentration and death is not that obvious. The second criterion was moderate toxicity on CD8+ T cells. We then investigated whether or not DCMU was toxic for T cells around a wide range of concentrations. No significant modification on cell viability was observed following a 24-h exposure to either 10 or 100 µM of DCMU (**Figure 1A**). An exposure to 250 µM of DCMU for 24 h moderately reduces percent live cells by about 16% (p<0.05).

To conclude, DCMU only slightly affects CD8+ T-cell viability at 250 µM. These concentrations are perfectly relevant in a context of acute poisoning; thus, we selected these concentrations to pursue our experiments.

### DCMU Changes miRNA and mRNA Expression Patterns in CD8+ T Cells

To evaluate the impact of DCMU exposure on miRNA expression patterns of CD8+ T cells, CTL03.1 cells were exposed to increasing doses of DCMU, and miRNA expression was analyzed using miRNA 4.0 arrays. We showed that numbers of dysregulated miRNA increased with the herbicide concentration of exposure. Overall, we found that 25, 99, and 189 mature miRNAs were significantly differentially expressed (DEmiRNA) in DCMU-treated conditions, namely, 10, 100, and 250 µM, respectively, compared to DMSO-treated ones (**Figure 1B**; **Supplementary Table S1**). Further in silico analysis described molecular pathways targeted by these DCMU-dysregulated miRNAs (**Figure 1C**), including numerous pathways related to cancer and, interestingly, the T-cell receptor signaling pathway at all DCMU concentration exposures [TcRSP; Kyoto Encyclopedia of Genes and Genomes (KEGG) # hsa04660, p<0.001, **Supplementary Table S2**].

Since miRNA target mRNAs to downregulate their expression, we conducted next a 3′ sequencing RNA profiling of CTL03.1 population exposed to DCMU. Once again, the number of significantly DEmRNAs increased with the dose of DCMU ranging from 51 mRNAs at 10 µM up to 574 at 250 µM (**Figure 1D**; **Supplementary Table S3**). KEGG pathway analysis showed that the dysregulated mRNAs were significantly enriched for various functions (**Supplementary Figure S1**), including the T-cell receptor signaling pathway (p<0.001) among the top 10 of the enriched pathways at 250 µM of DCMU (**Figure 1E**).

Interestingly, more than 80% of the dysregulated pathways identified through our 3′RNA sequencing analysis are also identified through our miRNA analysis, suggesting that a large part of the mRNA expression changes might be imputable to dysregulated miRNA.

Moreover, DCMU did not impact the expression of genes involved in miRNA biogenesis, suggesting that the increasing number of dysregulated miRNA in correlation to DCMU doses was not due to alterations of miRNA biogenesis pathways (**Supplementary Figure S2**).

To conclude, DCMU modulates both miRNA and mRNA expressions in a dose-dependent manner. Pathways involved in T-cell functions were enriched in analyses based on both miRNA and mRNA expression, suggesting that DCMU does exert, at least partially, its effects on CD8+ T-cell functions through miRNA dysregulation that leads to a dysregulation of mRNA targets.

### DCMU Alters CD8+ T-Cell Anti-Tumor Functions

**Figure 2:**
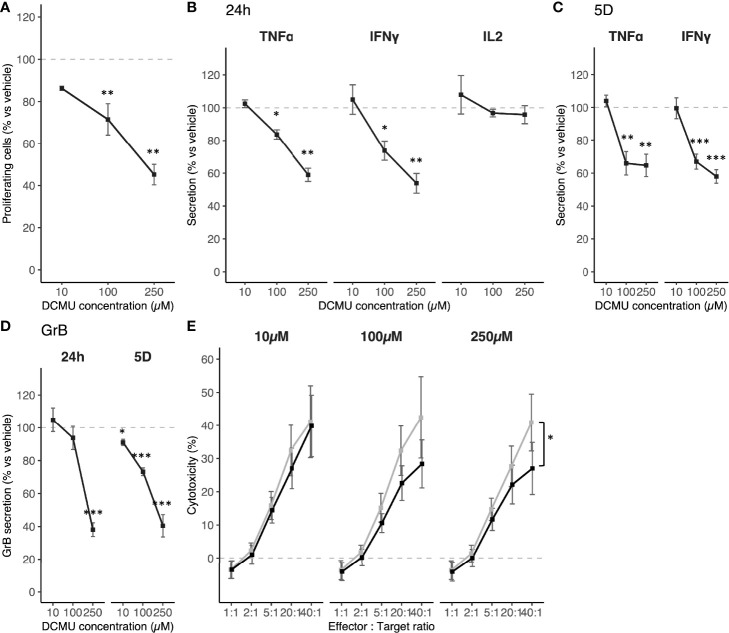
DCMU exposures impact on human CTL03.1 cells in vitro. **(A)** CTL03.1 proliferation under DCMU exposures. **(B)** TNF-α, IFN-y and IL-2 protein concentrations in supernatant of CTL03.1 cells exposed to DCMU during 24h. **(C)** TNF-α and IFN-y protein concentrations in supernatant of CTL03.1 cells 4 days after the end of DCMU exposures. **(D)** Granzyme B protein concentrations in supernatant of CTL03.1 cells exposed to DCMU during 24h (24h) or 4 days after the end of exposure (5D). **(E)** Cytotoxicity assays against M113 cells by CTL03.1 cells. In black CTL03.1 cells exposed to DCMU, in grey CTL03.1 cells exposed to vechicle only. Each time 3 independent experiments were run in triplicate per condition. Statistical significance. * p≤.05, ** p≤0.01, *** p≤0.001.

We next evaluated if these DCMU-induced dysregulations of miRNA, and their consequences on mRNA expressions, impact mechanisms involved in CD8+ T-cell antitumor functions. We first quantified proliferation abilities of CTL03.1 following a 24-h exposure to DCMU under an activation using CD3/CD28 beads (**Figure 2A**). A significant concentration-dependent decrease was observed in CTL03.1 cells, ranging from a 13% decrease at 10 µM up to 55% at 250 µM.

Then, we measured cytokine secretions of CTL03.1 cells [tumor necrosis factor α (TNF-α), interferon-γ (IFN-γ), and interleukin 2 (IL-2)] under DCMU exposures. Again, we first activated CD8+ T cells using CD3/CD28 beads and exposed them to increasing DCMU doses (**Figure 2B**). We observed that a 24-h exposure to DCMU induces a similar pattern of secretion for TNF-α and IFN-γ. Although no significant difference in cytokine secretion was observed at 10 µM DCMU 24 h following exposure, a large alteration was measured for both 100 and 250 µM concentrations. TNF-α secretion was significantly reduced by approximately 20% at 100 µM (p < 0.05) and by 40% at 250 µM of DCMU (p < 0.01). IFN-γ secretion was also significantly reduced from 25% at 100 µM (p < 0.05) up to a 46% decrease at 250 µM exposure to DCMU (p < 0.01). No significant difference was observed on IL-2 cytokine production levels in CTL03.1 after exposure to the herbicide. We also evaluated the long-lasting effect of such an exposure. Briefly, cells were washed from DCMU after a 24-h exposure and maintained in cell culture for several days. Cytokine levels were measured 4 days later, and, interestingly, effects were still significant for both TNF-α and IFN-γ (**Figure 2C**). Their secretions were still impaired by more than 30% in conditions that were exposed to 100 and 250 µM of DCMU at day 5. This demonstrates that even a short exposure may induce long-lasting effects on the activation of CD8+ T cells.

We also evaluated granzyme B (Gr-B) secretion, a serine protease contained into CD8+ T-cell lytic granules (**Figure 2D**, panel 24h). An exposure to 100 µM of DCMU induces a significant reduction in Gr-B secretion by 20% in CTL03.1 (p < 0.05) and by 70% following a 250-µM exposure (p < 0.001). Once more, Gr-B secretion was analyzed 4 days after a 24-h exposure to DCMU, and, interestingly, Gr-B secretion was still significantly downregulated with similar alterations as observed right after DCMU exposure. Secretion alterations even are significant in conditions exposed to 10 µM of DCMU (reduction by 9%, p < 0.05) even though this was not the case right after exposure (**Figure 2D**, panel 5D).

Finally, we measured CD8+ T-cell abilities to kill melanoma tumor cells. To do that, we performed a coculture with adequate target melanoma cells (M113; presenting the specific antigen Melan-A) and CTL03.1 cells. We found that CTL03.1 cells were impaired, in a DCMU concentration-dependent manner, in their abilities to recognize and kill cell line expressing its specific antigen (**Figure 2E**, **Supplemental Figure S3A**, p < 0.05).

Altogether, these results reveal that a short exposure to DCMU is sufficient to induce major and persistent alterations in CD8+ T-cell functions including not only proliferation, secretion of cytokines, and granzyme B but also cytotoxicity.

### DCMU Impairs CD8+ T-Cell Functions by Dysregulating Their miRNAs

**Figure 3:**
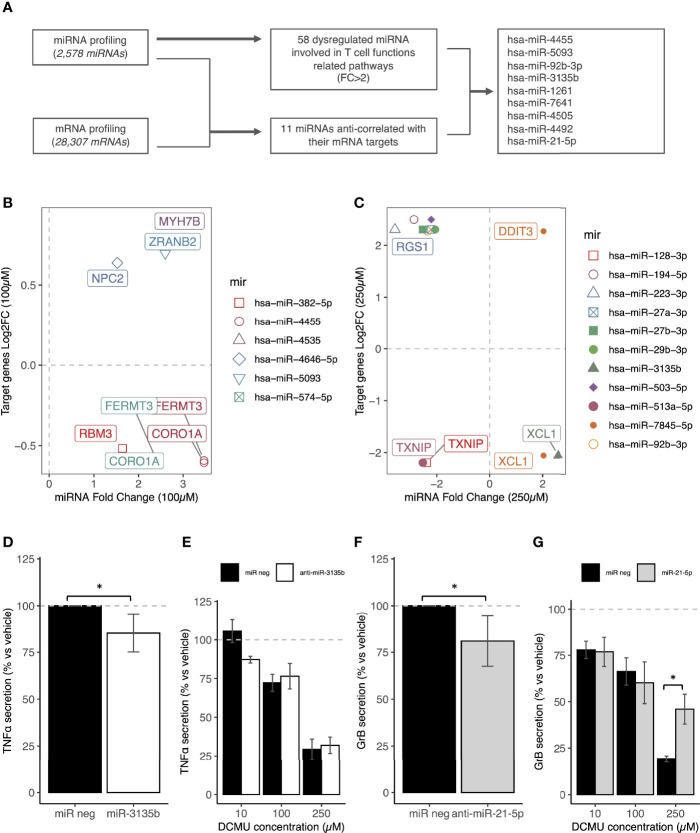
miRNA transfections mimic DCMU- induced alterations in CD8+T cells. **(A)** Flowchart of the miRNA and mRNA analyses of CTL03.1 cells exposed to DCMU in order to identify candidate miRNAs. **(B)** Identification of the upregulated miRNAs/downregulated mRNAs and the downregulated miRNAs/upregulated mRNAs couples after an exposure to a DCMU dose of 100µM. **(C)** Identification of the upregulated miRNAs/downregulated mRNAs and the downregulated miRNAs/upregulated mRNAs couples after an exposure to a DCMU dose of 250µM. **(D)** TNF-α protein concentrations in supernatant of CTL03.1 cells transfected with a miR-3135b mimic or a negative control miRNA during 24h. **(E)** TNF-α protein concentrations in supernatant of CTL03.1 cells exposed to DCMU and transfected with antimiR-3135b during 24h. **(F)** Gr-B protein concentrations in supernatant of CTL03.1 cells transfected with an anti-miR-21-5p or a negative control miRNA during 24h.**(G)** Gr-B protein concentrations in supernatant of CTL03.1 cells exposed to DCMU and transfected with a miRNA-21-5p mimic during 24h. Each time 3 independent experiments were run in triplicate per condition. Statistical significance. * p≤.05.

We selected next, candidate miRNAs by two approaches (**Figure 3A**). First, we tried to establish DEmiRNA–DEmRNA regulatory networks. Targets of DEmiRNA were first predicted using three databases implemented in R. Identified genes were next crossed with DEmRNAs obtained from our sequencing data. Common genes both belonging to DEmiRNA targets and DEmRNAs that have inverse expression relationship with DEmiRNA were selected and plotted in **Figures 3B, C**. Second, since miRNAs also regulate gene expression by translational repression without alteration of the mRNA amount (3), we selected other candidate DEmiRNAs based this time on an FC above 2 or −2 and with at least one target gene in T-cell function/activation pathways (**Table 1**).

**Table 1:** List of candidate miRNAs based on the target prediction analysis.

miRNA	Targets	DCMU doses	log2FC	P-value
hsa-miR-4646-5p	*VAV3, MAPK1, PIK3CA, FOS*	10	1.13	0.0004
hsa-miR-1261	*MAPK1, DLG1, PIK3R1, PIK3CB*	100	2.09	0.0128
hsa-miR-1299	*JUN, PIK3CA, NFATC3, PIK3CB, CBLB, PDPK1, CD28, PAK3, TNF, PAK5*	100	1.03	0.0035
hsa-miR-185-3p	*PAK1, PAK4, MAPK1, VAV2, MAPK13, MAP3K14, ZAP70, PIK3R3, MAPK14*	100	1.92	0.0261
hsa-miR-3135b	*RELA, NFATC1, NFATC2, RHOA, NRAS, MAPK1, PIK3R3, GRB2, AKT3, AKT2*	100	1.09	0.0011
hsa-miR-3911	*CD40LG, NCK2, PDCD1, MAPK10, PIK3CB, AKT2, MAPK1, RAF1, DLG1, IKBKB, PDPK1, PIK3CA, PPP3CB*	100	-1.01	0.0071
hsa-miR-4284	*MAPK1, SOS1, PDPK1, PIK3CA, RELA, NFKBIB*	100	1.96	0.0189
hsa-miR-4455	*TEC, AKT2, PTPRC, CD8B, CD4, CD28, NCK2, GSK3B, SOS1*	100	1.79	0.0091
hsa-miR-4535	*MAPK1, CD28*	100	1.65	0.0183
hsa-miR-4710	*CD4, AKT2, PDPK1, GSK3B, MAPK14, AKT3, NFATC2, PIK3R2*	100	1.04	0.0344
hsa-miR-4717-3p	*GSK3B, MAPK10, PIK3R1, PPP3R1, AKT3, PAK6, PPP3CB, PIK3R3, PIK3CB, MAPK14, FOS*	100	1.42	0.0110
hsa-miR-5093	*CBLB, RASGRP1, GSK3B, KRAS, PIK3CB, AKT3, SOS1, NFATC2, FYN*	100	1.38	0.0466
hsa-miR-5189-3p	*PPP3R1, PLCG1*	100	-1.22	0.0065
hsa-miR-574-5p	*CD40LG, NCK2, PAK3, PRKCQ, AKT3, MAPK10*	100	1.27	0.0494
hsa-miR-595	*AKT3*	100	1.06	0.0167
hsa-miR-6726-5p	*MAPK12, CD8B, AKT3*	100	1.09	0.0279
hsa-let-7f-5p	*RASGRP1, MAPK8, NRAS, ICOS, IL10, TEC, VAV3, PIK3CA*	250	-1.26	0.0096
hsa-miR-1229-5p	*MAPK1*	250	1.09	0.0205
hsa-miR-128-3p	*MAPK14, MAP3K8, SOS1, GSK3B, PIK3R1*	250	-1.25	0.0336
hsa-miR-146b-5p	*DLG1, NRAS, RASGRP1, FYN, RAF1, PIK3CB, MAP3K8*	250	-1.01	0.0006
hsa-miR-1587	*PAK3, PPP3R2, IL10, CTLA4, GRAP2, RAF1, IKBKB, NFATC2*	250	1.04	0.0078
hsa-miR-15a-5p	*AKT3, MAPK8, CD28, PIK3R1, IKBKB, NFATC3, RAF1, MAPK9, SOS2, CD3E, CHUK, PDCD1, CDC42, PAK5, GSK3B, MAP3K7, PIK3R3*	250	-1.41	0.0029
hsa-miR-192-5p	*PAK6, GSK3B*	250	-1.51	0.0016
hsa-miR-194-5p	*PPP3CA, AKT2, PPP3R1, IL10, MAPK1, PAK2*	250	-1.52	0.0128
hsa-miR-195-5p	*AKT3, IKBKB, MAPK8, CD28, NFATC3, PIK3R1, RAF1, MAPK9, SOS2, CD3E, CHUK, PDCD1, CDC42, GSK3B, PIK3R3, PAK5*	250	-1.26	0.0227
hsa-miR-197-3p	*MAPK8, MAPK10, PPP3R2, SOS1*	250	-1.08	0.0027
hsa-miR-21-5p	*RASGRP1, PIK3R1, SOS2, LCP2, RAF1*	250	-1.44	0.0011
hsa-miR-223-3p	*CBLB, RASGRP1, RELA, CSF2, TEC*	250	-1.84	0.0388
hsa-miR-23a-5p	*TEC, PDCD1, NCK2, PAK2*	250	-1.71	0.0041
hsa-miR-23b-3p	*PIK3R3, PDPK1, CHUK, PAK2, PIK3CB, GSK3B, PAK3, MAPK14, CBLB, MAP3K8, PTPRC, MAPK10, GRAP2, TEC*	250	-1.07	0.0011
hsa-miR-26b-5p	*GSK3B, PRKCQ, PAK2, PPP3CB, PPP3R1, CBLB, PAK1, IFNG, NRAS*	250	-1.44	0.0191
hsa-miR-27a-3p	*GRB2, SOS1, MAPK14, VAV3, GSK3B, MAP2K7, PDPK1, IFNG, VAV2, PAK6, PIK3CA, CBLB, ICOS, CD28, KRAS, PPP3R1, MAPK10, TEC, MAP3K7, PIK3R1, NRAS, IL10*	250	-1.15	0.0005
hsa-miR-27b-3p	*GRB2, SOS1, MAPK14, GSK3B, VAV3, MAP2K7, PDPK1, IFNG, VAV2, PAK6, CBLB, ICOS, CD28, PPP3R1, PIK3CA, KRAS, MAPK10, TEC, MAP3K7, PIK3R1, NRAS, IL10*	250	-1.34	0.0079
hsa-miR-29b-3p	*AKT3, PIK3R1, ICOS, MAPK10, AKT2, CDC42, IFNG, GSK3B, GRAP2, NFATC3, FOS, NRAS, FYN, PIK3CB*	250	-1.04	0.0065
hsa-miR-30c-5p	*PPP3R1, PIK3CD, NFATC3, MAPK8, NFATC2, BCL10, PPP3CA, CBLB, SOS1, PAK5, FYN, NCK2, VAV3, KRAS, PPP3CB, MAP3K7, LCP2, PLCG1, SOS2, ICOS, PIK3CA, PIK3R2*	250	-1.15	0.0008
hsa-miR-3135b	*RELA, NFATC1, NFATC2, RHOA, NRAS, MAPK1, PIK3R3, GRB2, AKT3, AKT2*	250	1.37	0.0002
hsa-miR-365b-5p	*CD247, CD8B, PIK3CA, LCP2*	250	-1.07	0.0368
hsa-miR-3911	*CD40LG, NCK2, PDCD1, MAPK10, PIK3CB, AKT2, MAPK1, RAF1, DLG1, IKBKB, PDPK1, PIK3CA, PPP3CB*	250	-1.36	0.0009
hsa-miR-421	*NRAS, LCK*	250	-1.34	0.0378
hsa-miR-424-3p	*FOS, PIK3R3, PIK3R1*	250	-1.23	0.0051
hsa-miR-4462	*MAPK9*	250	1.46	0.0276
hsa-miR-4472	*PIK3R2, GSK3B, AKT2, TEC, NRAS*	250	1.28	0.0455
hsa-miR-4492	*PAK4, RELA, PPP3R2, MAPK1, VAV2, NFATC2, MAP2K7, RAF1, ITK, AKT2, CD8A, MAP3K14, CARD11, HRAS, GRB2, PDPK1, AKT3, TNF, PRKCQ, IL10, LAT, NFATC1, PIK3R3, CD4*	250	1.11	0.0001
hsa-miR-4521	*AKT3, FOS, MAP2K2, MAPK13*	250	-2.10	0.0037
hsa-miR-4530	*CD3E, ICOS, NFKB1, NRAS, MAPK1, CD4*	250	1.15	0.00008
hsa-miR-454-3p	*SOS2, PIK3CB, PAK6, MAPK10, FYN, MAPK8, TNF, CBLB*	250	-1.46	0.0035
hsa-miR-4647	*GSK3B, MAPK9, CD8B, SOS2, NCK2, PIK3R3, AKT3*	250	1.24	0.0222
hsa-miR-4669	*PIK3CA, CD8B*	250	-1.57	0.0144
hsa-miR-503-5p	*AKT3, PIK3R1, CD28, PPP3CB, RAF1, MAPK8*	250	-1.14	0.0407
hsa-miR-510-5p	*MAPK8, NRAS, PIK3CD, NFKBIE*	250	-1.45	0.0176
hsa-miR-513a-5p	*KRAS, GRB2, IFNG, ICOS, MAPK14, VAV2, MAP3K7, PIK3CB, PPP3R1, TEC, CBLB, SOS1, PIK3CA, PDPK1, PAK6, CD28, PPP3CA, NRAS, GSK3B, IL10, PIK3R1*	250	-1.34	0.0048
hsa-miR-513b-5p	*PIK3R3, PPP3CA, PAK1, PAK6, PPP3R1, KRAS, SOS2, RASGRP1, SOS1, MAPK1*	250	-1.98	0.0075
hsa-miR-5196-5p	*PPP3R1, GRB2, PIK3R2, MAPK9, CDC42, CD3D, CBLB, CDK4, CD8A, PIK3R3, PPP3R2, GSK3B, AKT1, PAK2, TNF, MAPK10*	250	1.15	0.0161
hsa-miR-6880-5p	*CD4, GRB2*	250	-1.14	0.0014
hsa-miR-7162-3p	*VAV3, RASGRP1, BCL10, PPP3CB*	250	-1.19	0.0034
hsa-miR-7641	*PIK3R1, MAPK8*	250	1.36	0.0004
hsa-miR-7845-5p	*SOS1, CDK4*	250	1.02	0.0012
hsa-miR-92b-3p	*PIK3CB, MAPK8, PIK3R3, PIK3CA, GSK3B, CDC42, JUN*	250	-1.23	0.0165

Non-exposed CTL03.1 cells were then transfected with each of this candidate miRNA (500 nM) to see if they can mimic DCMU-induced alterations. Cytokine secretions were next measured by ELISA as previously described. We found that numerous candidate miRNA can modulate cytokine secretions as induced by DCMU exposures (**Supplementary Figures S4A–C**).

As a proof of concept, we then pursued our investigations with only two miRNAs, one upregulated by DCMU exposure, hsa-miR-3135b, and one downregulated by it, hsa-mir-21-5p (**Supplementary Figure S5**). First, we transfected miR-3135b mimic to CD8+ T cells unexposed to DCMU in order to evaluate if the decreased cytokine secretion could be mirrored. Indeed, transfection of the miR-3135b mimic reduced TNF-α secretion by about 15% (p < 0.05, **Figure 3D**; **Supplementary Figure S4B**). To validate that the decrease in TNF-α secretion was due to the upregulation of hsa-miR-3135b by DCMU, we transfected next an antimiR-3135b in CD8+ T cells this time exposed to DCMU during 24 h to counteract its overexpression induced by the herbicide. However, we failed to significantly restore the TNF-α secretion with this antimir alone (**Figure 3E**); the upregulation of hsa-mir-3135b by DCMU is probably larger than expected, or other miRNA or mechanisms are also involved. Moreover, according to our prediction target analysis, hsa-mir-3135b does not directly target *TNFA*, but our 3′RNA sequencing analysis identified *XCL1* as a potential target of hsa-miR-3135b (**Figure 3C**), which we confirmed by RT-qPCR (**Supplementary Figure S5C**). XCL1 could be a credible intermediate between TCR activation and TNF-α secretion.

Second, the transfection of an anti-mir-21-5p in CTL03.1 cells significantly decreased about more than 10% the Gr-B secretion (p < 0.05, **Figure 3F**; **Supplementary Figure S4C**), as observed under DCMU exposure. We pursued then our investigation by transfecting its mimics in CTL03.1 cells exposed to DCMU during 24 h, to counteract artificially its downregulation induced by the herbicide. Interestingly, Gr-B secretion was partially but significantly restored in CTL03.1 cells transfected with hsa-miR-21-5p mimic and exposed to DCMU (p < 0.05, **Figure 3G**).

To conclude, miRNAs are interesting candidates to link DCMU exposure to altered CD8+ T-cell antitumor functions.

### DCMU Reduces Abilities of Different T-Cells Models

**Figure 4:**
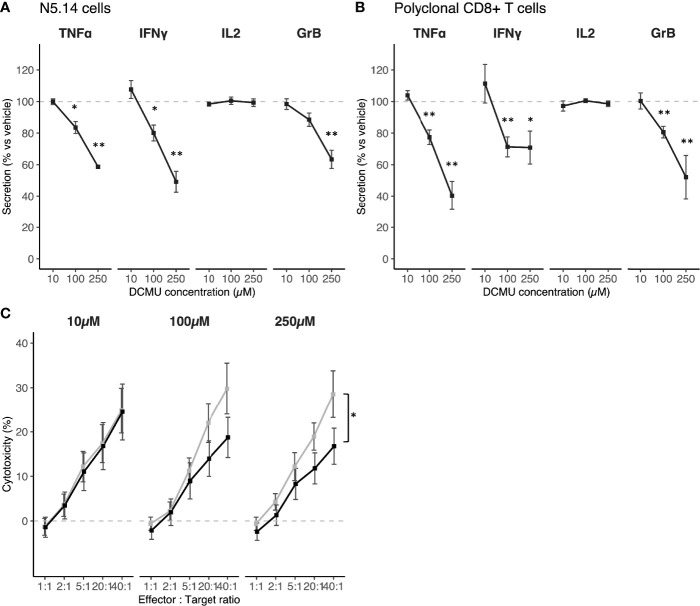
Validation of the effect of DCMU exposures on different CD8+T cell populations. **(A)** TNF-α, IFN-γ, IL-2 and Gr-B protein concentrations in supernatant of N5.14 cells exposed to DCMU during 24h. **(B)** TNF-α, IFN-γ, IL-2 and Gr-B protein concentrations in supernatant of polyclonal CD8+ T cells exposed to DCMU during 24h. **(C)** Cytotoxicity assays against Meso34 cells by N5.14 cells. In black, N5.14 cells exposed to DCMU, in grey N5.14 cells exposed to vehicle only. Each time 3 independent experiments were run in triplicate per condition. Statistical significance: * p≤.05, ** p≤0.01.

We next try to confirm these results in different models of T cells. First, we investigated in the same way the impact of DCMU on another cancer-specific CD8+ T-cell clone, this time specific for the mesothelioma antigen MUC1 (N5.14). We observed that a 24-h exposure to DCMU reduces TNF-α secretion by approximately 20% at 100 µM (p < 0.05, **Figure 4A**). The decrease is more significant when cells are exposed to 250 µM of DCMU (p < 0.01, **Figure 4A**). IFN-γ secretion was also significantly reduced by 20% at 100 µM (p < 0.05) and by more than 50% at 250 µM (p < 0.01, **Figure 4A**), as previously described for CTL03.1 cells. As observed with the Melan-A specific CD8+ T-cell clone, DCMU exposure did not impact IL-2 secretion by N5.14 cells (**Figure 4A**).

Next, we evaluated the impact of DCMU exposure on polyclonal CD8+ T cells freshly sorted from healthy donors. Here, we sought to investigate the impact of DCMU in the immune surveillance against cancer. DCMU induced a pattern of cytokine secretions in freshly isolated CD8+ T cells, which is similar to that in CTL03.1 and N5.14 cells (**Figure 2**). Indeed, TNF-α secretion was significantly reduced by approximately 20% at 100 µM (p < 0.05, **Figure 4B**) and by 60% at 250 µM (p < 0.01, **Figure 4B**). IFN-γ secretions were also significantly reduced at 100 and 250 µM exposure to DCMU (**Figure 4B**). Again, DCMU exposure did not impact IL-2 secretion by polyclonal CD8+ T cells (**Figure 4B**).

Finally, we evaluated cytotoxicity functions, through granzyme B release and cytotoxicity as described earlier for CTL03.1 clone. Gr-B secretion is, as observed for CTL03.1 cells, largely reduced by DCMU in a dose-dependent manner in N5.14 and polyclonal CD8+ T cells (**Figures 4A, B** p < 0.01 at 250 µM). Next, we performed coculture with N5.14 cells and Meso13, a mesothelioma cell line presenting the specific antigen MUC1. Again, we replicated the results that we found in CTL03.1 cells, since N5.14 cells exposed to 250 µM DCMU had a significant decline in their ability to induce the death of mesothelioma cells (**Figure 4C**; **Supplementary Figure S1**, p < 0.05).

To conclude, all tested CD8+ populations are equally affected by DCMU.

### DCMU Reduces T Cell Functions and Favor Tumor Development in a Zebrafish Model

**Figure 5:**
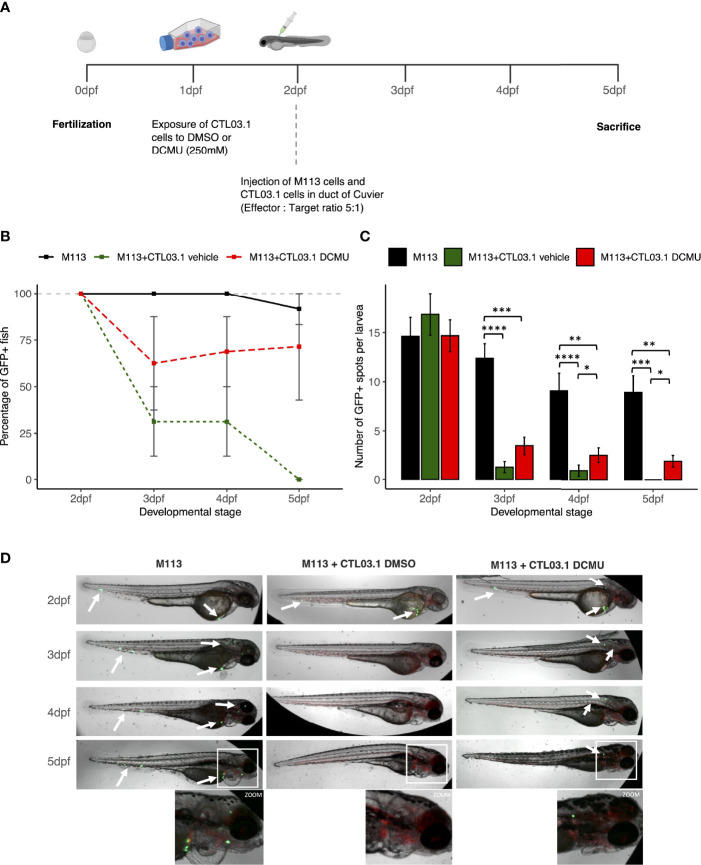
DCMU exposures create a permissive environment to tumor development. **(A)** Schematic diagram of the experimental design. **(B)** Percent of GFP+ larvae. In black, zebrafish injected with M113 cells alone, in dark green, zebrafish injected with M113 and CTL03.1 cells exposed to vehicle and in red, zebrafish co-injected with M113 and CTL03.1 cells exposed to DCMU (250 µM). **(C)** Quantification of GFP+ spots per larvae. In black, zebrafish injected with M113 cells alone, in darkgreen, zebrafish co-injected with M113 and CTL03.1 cells exposed to vehicle and in red, zebrafish co-injected with M113 and CTL03.1 cells exposed to DCMU (250μM).**(D)** Representative lateral view of zebrafish larvae bearing M113 cells (green) in the three groups. Zoom 2.5 x. Mean of 3 independent experiments, n=8 larvae per group, per experiment. Statistical significance. * p≤.05, ** p≤0.01, *** p≤0.001. **** p≤0.0001.

Finally, since DCMU exposure alters anti-tumor functions and immune surveillance, we sought to determine whether CD8+ T cells exposed to DCMU are more prone to favor tumor development in zebrafish. To visualize tumor development, we first transduced melanoma M113 cells with a green fluorescent protein (GFP) and injected them alone, or with CTL03.1 cells at a ratio of 1:5, into the duct of Cuvier of larvae (**Figure 5A**). At 24 h post-implantation [3 days post fertilization (dpf)], all zebrafish larvae injected with M113 cells alone remained fluorescent, indicating that tumor development can occur (**Figures 5B, D**). In contrast, co-injection of M113 and CTL03.1 cells resulted in a large elimination of tumor cells in zebrafish, since only 31% of the larvae remained fluorescent at 3 dpf and no one at 5 dpf. When CTL03.1 cells were pretreated before injection with DCMU (250 µM), more than 60% of larvae remained fluorescent at 3 or 5 dpf (**Figure 5B**). Interestingly, the number of GFP+ spots per larvae is also significantly higher in zebrafish injected with M113 alone than those co-injected with CTL03.1 cells exposed or not to DCMU. Indeed, at 48 and 72 h post-implantation (4 and 5 dpf), we observed more GFP+ spots per larvae in zebrafish injected with CTL03.1 cells exposed to DCMU than those exposed to vehicle (p < 0.05) (**Figures 5C, D**).

Overall, CD8+ T cells exposed to DCMU create a permissive environment to tumor development in zebrafish model.

## Discussion

CD8+ T cells constantly screen blood, lymph, tissues, and organs for potential neoplastic cells. It is therefore crucial to better understand the impact of environmental pollutants, such as DCMU, on immunocompetent cells. In the present work, we provide consistent experimental data for the understanding of the effect of DCMU on human CD8+ T cells and show how pollutant not only can dysregulate T cells miRNAs and mRNAs but also reduce lymphocyte functions to favor tumor development and proliferation.

TNF-α is a well-known cytokine secreted by activated CD8+ T (28, 29). IFN-γ is a cytokine with antiviral, antitumor, and immunomodulatory properties, mainly secreted by CD8+ T cells. It induces apoptosis of cancer cells by activating JAK-STAT1-caspase signaling but interacts also with several cytokines/cells from the tumor microenvironment to induce cancer growth arrest (30). In this work, we demonstrated that exposure to the herbicide DCMU alters both TNF-α and IFN-γ secretion by CD8+ T cells in a dose-dependent manner. This decrease in cytokine secretion might be deleterious for the immune response, since both TNF-α and IFN-γ usually act toward the promotion of the inflammation by inducing cell death in targeted cell with the additional involvement of Fas/FasL interactions (31). Another pro-inflammatory role driven by these cytokines is to facilitate dendritic cell maturation and CD8+ T-cell activation and tumor infiltration, thus strengthening the immune response at the site of inflammation (32). However, TNF-α can also constrain the immune activation especially by increasing number of regulatory T (Treg), B cells, and myeloid-derived suppressor cells (MDSC), thus avoiding an overactivation upon the site of inflammation. DCMU-related effects on the maintenance of the immune response through other cell types is also a key point that might be addressed in future work to fully understand the effect of DCMU on the human immune system.

We also described that DCMU effects on CD8+ T cells can be long lasting and thus impact T-cell functions for a larger period of time than the time of exposure itself. This observation is of importance, since this might render the organism more susceptible to neoplastic aggressions over time following exposure to herbicides such as DCMU. Cytokine secretion dysregulations induced by pesticides have been largely documented for other biocides than DCMU and is surely an important link in the chain of creating a conducive environment for the development of pathologies such as cancers, as it severely impairs immune surveillance (33). Our *in vivo* model confirmed the creation of a permissive environment by DCMU.

One major advancement of our work is the demonstration of the role of miRNA in DCMU immunotoxicity. It is well known that toxic environmental factors, notably air pollutants such as tobacco, can alter miRNA expression profiles, but it is less investigated in the biocide field. We found that DCMU alters miRNA patterns of CD8+ T cells in a dose-dependent manner, without modifying the miRNA biogenesis pathway. Alterations of miRNA expression profiles affect in turn the gene network targeted by miRNA and can lead to dysfunctional cells. An interesting candidate is hsa-miR-3135b, induced after DCMU exposure. This miRNA, previously associated with acute coronary syndrome (34), malaria (35), and ovarian carcinoma (36), has no functionally validated target gene. Our results showed that XCL1 could be a good candidate target in CD8+ T cells. XCL1 is a polypeptide secreted by CD8+ T cells upon activation. It increases the survival of these cells and their differentiation to IFN-γ secreting effectors after antigen contact, and more importantly, its absence impaired the development of antigen-specific cytotoxicity *in vivo* (37). Its upregulation by hsa-miR-3135b could explain the deleterious effects of DCMU on CD8+ T cells.

Interestingly, our miRNA screen analysis also identified candidates, such as hsa-miR-21-5p, downregulated by a DCMU exposure. hsa-miR-21 was previously associated with the detoxication of environmental pollutants through their potential target genes CYP1A1 (cytochrome P450, family 1, subfamily A, polypeptide 1) and CYP2B1 (cytochrome P450, family 2, subfamily B, polypeptide 1) (38). Although the link between miRNA and mRNA still needs to be firmly established, our data strengthened the idea that acute exposure to DCMU can, through miR-21-5p downregulation, solicitate the detoxification machinery in human cells, in line with what have been observed previously by Rudzok and colleagues (39). In their study, they demonstrated that CYP1A1 mRNA expression is largely increased upon DCMU exposure.

Individually, each miRNA showed a slight effect on T-cell functions, but the dysregulation of several miRNA as observed under DCMU exposure leads to large alterations of the CD8+ T-cell functions. Although this work has been conducted only under a range of concentrations matching acute exposure, it will be interesting to see if the observed dysregulations of miRNA are maintained at lower DCMU concentrations. Indeed, miRNAs are also promising means to follow exposure to environmental pollutants, even before the onset of a disease. There is a growing body of evidence suggesting that miRNA can be used as potential biomarkers for pesticide exposures (40, 41). The measurement of miRNA from CD8+ T cells in large cohort could be easy, since only a blood sample will be necessary. It could help to identify workers or people living in agricultural area that could be at risk to develop serious illness due to an altered immune response.

To conclude, we demonstrated that exposure to the herbicide DCMU affects human CD8+ T cells by decreasing not only their cytokine secretion abilities but also their cytotoxicity in several CD8+ T-cell populations. We also highlighted that DCMU exposure, as observed already for other environmental pollutants, dysregulates miRNA, and we identified *in silico* some leads that might be interesting to follow in further work, notably hsa-miR-21-5p and hsa-miR-3135b. Overall, CD8+ T-cell immune capacities are impaired by DCMU exposure, and this might participate to the establishment of a suitable environment for the development of cancers. This work provides strong basis to study environmental pollutant effects on immune cells by combining transcriptomic, functional, and *in vivo* analysis. Further investigation will be required to evaluate whether or not our observations could, to some extent, be shared by other pattern of exposure (mimicking chronic exposure for instance) or to assess the impact that such exposures (acute or chronic) might have on other cells types. Altogether, our result describes for the first time the deleterious effects of DCMU acute exposure to human CD8+ T-cell functions.

## Data Availability Statement

The datasets presented in this study can be found in online repositories. The names of the repository/repositories and accession number(s) can be found below: NCBI’s Gene-Expression Omnibus database under references GSE189440 and GSE189443.

## Ethics Statement

Ethical review and approval was not required for the animal study because the Animals (Scientific Procedures) Act 1986 only regulates fish from the time at which they become capable of independent feeding (after 6 days post-hatching).

## Author Contributions

PA, CB, and DF conceived and designed the study. PA, SD, and DF conducted experiments. NB, JL, and RL conducted *in vivo* studies. ED, J-FF, and NL contributed to T-cell clone culture. PA and DF wrote the manuscript. All authors reviewed and provided comments on the manuscript.

## Funding

Our research was funded by INSERM, CNRS, and grants from “Ligue contre le Cancer, Comités 16, 22, 29, 35, 44, 56.” This work was realized in the context of the LabEX IGO program supported by the National Research Agency *via* the investment of the future program ANR-11-LABX-0016-01. This work was realized in the context of the SIRIC ILIAD program supported by the French National Cancer Institute national (INCa), the Ministry of Health, and the Institute for Health and Medical Research (Inserm) (SIRIC ILIAD, INCa-DGOS Inserm-12558). PA was supported by a fellowship from EpiSAVMEN (“Dynamique scientifique” program of Région des Pays de la Loire).

## Conflict of Interest

The authors declare that the research was conducted in the absence of any commercial or financial relationships that could be construed as a potential conflict of interest.

## Publisher’s Note

All claims expressed in this article are solely those of the authors and do not necessarily represent those of their affiliated organizations, or those of the publisher, the editors and the reviewers. Any product that may be evaluated in this article, or claim that may be made by its manufacturer, is not guaranteed or endorsed by the publisher.
