# Supplementary material for: The DCMU Herbicide Shapes T-cell Functions By Modulating Micro-RNA Expression Profiles
Source: Front Immunol. 2022 Jul 28;13:925241. doi: 10.3389/fimmu.2022.925241 (PMC9366666; doi:10.3389/fimmu.2022.925241)
Supplement: Supplementary file 1 [file DataSheet_1.pdf]

Supplemental Table 1. miRNAs dysregulated in CTL03.1 cells in response to DCMU exposure during 24h.

DCMU dose (µM)	miRNA ID	Accession	species	pvalue	Fold change	Sequence_Type	Sequence_Source	Sequence
10	hsa-miR-1229-5p	MIMAT0022942	Homo_sapiens	0.0466313	-1.87471	miRNA	miRBase	GUGGGUAGGGUUUGGGGGAGAGCG
10	hsa-miR-8064	MIMAT0030991	Homo_sapiens	0.0456624	-1.68568	miRNA	miRBase	AGCACACUGAGCGAGCGGAC
10	hsa-miR-2277-5p	MIMAT0017352	Homo_sapiens	0.0395127	-1.65864	miRNA	miRBase	AGCGCGGGCUGAGCGCUGCCAGUC
10	hsa-miR-6715a-3p	MIMAT0025841	Homo_sapiens	0.01045	-1.55473	miRNA	miRBase	CCAAACCAGUCGUGCCUGUGG
10	hsa-miR-939-5p	MIMAT0004982	Homo_sapiens	0.0207082	-1.51222	miRNA	miRBase	UGGGGAGCUGAGGCUCUGGGGGUG
10	hsa-miR-2115-3p	MIMAT0011159	Homo_sapiens	0.0188104	-1.46747	miRNA	miRBase	CAUCAGAAUUCAUGGAGGCUAG
10	hsa-miR-6731-3p	MIMAT0027364	Homo_sapiens	0.0371884	-1.44727	miRNA	miRBase	UCUAUUCCCCACUCUCCCCAG
10	hsa-miR-5010-5p	MIMAT0021043	Homo_sapiens	0.0100017	-1.41421	miRNA	miRBase	AGGGGGAUGGCAGAGCAAAAUU
10	hsa-miR-4756-3p	MIMAT0019900	Homo_sapiens	0.0187881	-1.36289	miRNA	miRBase	CCAGAGAUGGUUGCCUUCCUAU
10	hsa-miR-5191	MIMAT0021122	Homo_sapiens	0.0146839	-1.32562	miRNA	miRBase	AGGAUAGGAAGAAUGAAGUGCU
10	hsa-miR-378b	MIMAT0014999	Homo_sapiens	0.0204055	-1.24258	miRNA	miRBase	ACUGGACUUGGAGGCAGAA
10	hsa-miR-105-3p	MIMAT0004516	Homo_sapiens	0.04282	-1.17283	miRNA	miRBase	ACGGAUGUUUGAGCAUGUGCUA
10	hsa-miR-152-3p	MIMAT0000438	Homo_sapiens	0.019522	1.1487	miRNA	miRBase	UCAGUGCAUGACAGAACUUGG
10	hsa-miR-5586-5p	MIMAT0022287	Homo_sapiens	0.0364058	1.15402	miRNA	miRBase	UAUCCAGCUUGUUACUAUAUGC
10	hsa-miR-362-5p	MIMAT0000705	Homo_sapiens	0.0412302	1.23399	miRNA	miRBase	AAUCCUUGGAACCUAGGUGUGAGU
10	hsa-miR-3688-3p	MIMAT0018116	Homo_sapiens	0.0467141	1.24545	miRNA	miRBase	UAUGGAAAGACUUUGCCACUCU
10	hsa-miR-548az-5p	MIMAT0025456	Homo_sapiens	0.046595	1.27162	miRNA	miRBase	CAAAAGUGAUUGUGGUUUUUGC
10	hsa-miR-3173-3p	MIMAT0015048	Homo_sapiens	0.0144389	1.29235	miRNA	miRBase	AAAGGAGGAAAUAGGCAGGCCA
10	hsa-miR-6516-5p	MIMAT0030417	Homo_sapiens	0.0337208	1.38831	miRNA	miRBase	UUUGCAGUAACAGGUGUGAGCA
10	hsa-miR-3150b-3p	MIMAT0018194	Homo_sapiens	0.00856524	1.45397	miRNA	miRBase	UGAGGAGAUCGUCGAGGUUGG
10	hsa-miR-4316	MIMAT0016867	Homo_sapiens	0.0257822	1.48452	miRNA	miRBase	GGUGAGGCUAGCUGGUG
10	hsa-miR-4721	MIMAT0019835	Homo_sapiens	0.0340021	1.65864	miRNA	miRBase	UGAGGGCUCCAGGUGACGGUGG
10	hsa-miR-6759-5p	MIMAT0027418	Homo_sapiens	0.00792058	1.65864	miRNA	miRBase	UUGUGGGUGGGCAGAAGUCUGU
10	hsa-miR-331-3p	MIMAT0000760	Homo_sapiens	0.00443292	1.99538	miRNA	miRBase	GCCCCUGGGCCUAUCCUAGAA
10	hsa-miR-4646-5p	MIMAT0019707	Homo_sapiens	0.000374428	2.18859	miRNA	miRBase	ACUGGGAAGAGGAGCUGAGGGA
100	hsa-miR-5189-3p	MIMAT0027088	Homo_sapiens	0.00657891	-2.33486	miRNA	miRBase	UGCCAACCGUCAGAGCCCAGA
100	hsa-miR-29b-1-5p	MIMAT0004514	Homo_sapiens	0.014143	-2.06575	miRNA	miRBase	GCUGGUUUCAUAUGGUGGUUUAGA
100	hsa-miR-3911	MIMAT0018185	Homo_sapiens	0.00718079	-2.01857	miRNA	miRBase	UGUGUGGAUCCUGGAGGAGGCA
100	hsa-miR-138-1-3p	MIMAT0004607	Homo_sapiens	0.0353617	-1.67018	miRNA	miRBase	GCUACUUCACAACACCAGGGCC
100	hsa-miR-5000-5p	MIMAT0021019	Homo_sapiens	0.0127029	-1.60956	miRNA	miRBase	CAGUUCAGAAGUGUUCCUGAGU
100	hsa-miR-455-3p	MIMAT0004784	Homo_sapiens	0.0476822	-1.59107	miRNA	miRBase	GCAGUCCAUGGGCAUAUACAC
100	hsa-miR-3663-3p	MIMAT0018085	Homo_sapiens	0.019121	-1.55833	miRNA	miRBase	UGAGCACCACACAGGCCGGGCGC
100	hsa-miR-7152-3p	MIMAT0028215	Homo_sapiens	0.0164698	-1.45734	miRNA	miRBase	UCUGGUCCUGGACAGGAGGC
100	hsa-miR-3646	MIMAT0018065	Homo_sapiens	0.0156459	-1.41748	miRNA	miRBase	AAAAUGAAAUGAGCCCAGCCCA
100	hsa-miR-3667-5p	MIMAT0018089	Homo_sapiens	0.0107815	-1.40444	miRNA	miRBase	AAAGACCCAUUGAGGAGAAGGU
100	hsa-miR-340-5p	MIMAT0004692	Homo_sapiens	0.0280107	-1.38191	miRNA	miRBase	UUAUAAAGCAAUGAGACUGAUU
100	hsa-miR-4736	MIMAT0019862	Homo_sapiens	0.00728462	-1.38191	miRNA	miRBase	AGGCAGGUUAUCUGGGCUG
100	hsa-miR-3662	MIMAT0018083	Homo_sapiens	0.0499809	-1.36604	miRNA	miRBase	GAAAAUGAUGAGUAGUGACUGAUG
100	hsa-miR-4781-3p	MIMAT0019943	Homo_sapiens	0.0198576	-1.35347	miRNA	miRBase	AAUGUUGGAAUCCUCGCUAGAG
100	hsa-miR-548j-3p	MIMAT0026737	Homo_sapiens	0.0102196	-1.33793	miRNA	miRBase	CAAAAACUGCAUUACUUUUGC
100	hsa-miR-8054	MIMAT0030981	Homo_sapiens	0.0106072	-1.33484	miRNA	miRBase	GAAAGUACAGAUCGGAUGGGU
100	hsa-miR-3913-5p	MIMAT0018187	Homo_sapiens	0.0229277	-1.32869	miRNA	miRBase	UUUGGGACUGAUCUUGAUGUCU
100	hsa-miR-4520b-3p	MIMAT0020300	Homo_sapiens	0.00886532	-1.31646	miRNA	miRBase	UUUGGACAGAAAACACGCAGGU
100	hsa-miR-3156-5p	MIMAT0015030	Homo_sapiens	0.0376279	-1.31342	miRNA	miRBase	AAAGAUCUGGAAGUGGGAGACA
100	hsa-miR-6812-5p	MIMAT0027524	Homo_sapiens	0.0443082	-1.31342	miRNA	miRBase	AUGGGGUGAGAUGGGGAGGAGCAGC
100	hsa-miR-4774-3p	MIMAT0019930	Homo_sapiens	0.00404913	-1.27456	miRNA	miRBase	AUUGCCUAACAUGUGCCAGAA
100	hsa-miR-550a-3p	MIMAT0003257	Homo_sapiens	0.0326382	-1.27456	miRNA	miRBase	UGUCUUACUCCCUCAGGCACAU
100	hsa-miR-640	MIMAT0003310	Homo_sapiens	0.00721819	-1.27162	miRNA	miRBase	AUGAUCCAGGAACCUGCCUCU
100	hsa-miR-7847-3p	MIMAT0030422	Homo_sapiens	0.0337809	-1.26284	miRNA	miRBase	CGUGGAGGACGAGGAGGAGGC
100	hsa-miR-7515	MIMAT0029310	Homo_sapiens	0.0344725	-1.25122	miRNA	miRBase	AGAAGGGAAGAUGGUGAC
100	hsa-miR-30c-2-3p	MIMAT0004550	Homo_sapiens	0.0215639	-1.24545	miRNA	miRBase	CUGGGAGAAGGCUGUUUACUCU
100	hsa-miR-5692c	MIMAT0022476	Homo_sapiens	0.0484063	-1.217	miRNA	miRBase	AAUAAUAUCACAGUAGGUGUAC
100	hsa-miR-5002-5p	MIMAT0021023	Homo_sapiens	0.0355711	-1.18921	miRNA	miRBase	AAUUUGGUUUCUGAGGCACUUAGU
100	hsa-miR-548y	MIMAT0018354	Homo_sapiens	0.0271253	-1.17013	miRNA	miRBase	AAAAGUAAUCACUGUUUUUGCC
100	hsa-miR-548ag	MIMAT0018969	Homo_sapiens	0.0289278	-1.15936	miRNA	miRBase	AAAGGUAAUUGUGGUUUCUGC
100	hsa-miR-7161-5p	MIMAT0028232	Homo_sapiens	0.0383806	-1.14605	miRNA	miRBase	UAAAGACUGUAGAGGCAACUGGU
100	hsa-miR-7108-5p	MIMAT0028113	Homo_sapiens	0.0106339	1.15936	miRNA	miRBase	GUGUGGCCGGCAGGCGGGUGG
100	hsa-miR-6791-5p	MIMAT0027482	Homo_sapiens	0.0278445	1.17827	miRNA	miRBase	CCCCUGGGGCUGGGCAGGCGGA
100	hsa-miR-7704	MIMAT0030019	Homo_sapiens	0.048799	1.21419	miRNA	miRBase	CGGGGUCGGCGGCGACGUG
100	hsa-miR-4488	MIMAT0019022	Homo_sapiens	0.039534	1.21982	miRNA	miRBase	AGGGGGCGGGCUCCGGCG
100	hsa-miR-1227-5p	MIMAT0022941	Homo_sapiens	0.0367387	1.23399	miRNA	miRBase	GUGGGGCCAGGCGGUGG
100	hsa-miR-3960	MIMAT0019337	Homo_sapiens	0.0144964	1.23971	miRNA	miRBase	GGCGGCGGCGGAGGCGGGGG
100	hsa-miR-5787	MIMAT0023252	Homo_sapiens	0.0136743	1.23971	miRNA	miRBase	GGGCUGGGGCGCGGGGAGGU
100	hsa-miR-762	MIMAT0010313	Homo_sapiens	0.00524132	1.29235	miRNA	miRBase	GGGGCUGGGGCCGGGGCCGAGC
100	hsa-miR-3945	MIMAT0018361	Homo_sapiens	0.0341821	1.31039	miRNA	miRBase	AGGGCAUAGGAGAGGGUUGAUAU
100	hsa-miR-1343-5p	MIMAT0027038	Homo_sapiens	0.0281633	1.32869	miRNA	miRBase	UGGGGAGCGGCCCCCGGGUGGG
100	hsa-miR-6804-5p	MIMAT0027508	Homo_sapiens	0.0377511	1.33176	miRNA	miRBase	UGAGGGUGUCAGCAGGUGACG
100	hsa-miR-4634	MIMAT0019691	Homo_sapiens	0.0306005	1.34102	miRNA	miRBase	CGGCGCGACCGGCCCGGGG
100	hsa-miR-665	MIMAT0004952	Homo_sapiens	0.0416409	1.34102	miRNA	miRBase	ACCAGGAGGCUGAGGCCCCU
100	hsa-miR-4505	MIMAT0019041	Homo_sapiens	0.0284432	1.35347	miRNA	miRBase	AGGCUGGGCUGGGACGGA
100	hsa-miR-6500-3p	MIMAT0025455	Homo_sapiens	0.0184594	1.35974	miRNA	miRBase	ACACUUGUUGGGAUGACCUGC
100	hsa-miR-6743-5p	MIMAT0027387	Homo_sapiens	0.000445323	1.37237	miRNA	miRBase	AAGGGGCAGGGACGGGUGGCCC
100	hsa-miR-92b-5p	MIMAT0004792	Homo_sapiens	0.0198784	1.37554	miRNA	miRBase	AGGGACGGGACGCGGUGCAGUG
100	hsa-miR-4530	MIMAT0019069	Homo_sapiens	0.0374555	1.37872	miRNA	miRBase	CCCAGCAGGACGGGAGCG
100	hsa-miR-4497	MIMAT0019032	Homo_sapiens	0.00467352	1.43065	miRNA	miRBase	CUCCGGGACGGCUGGGC
100	hsa-miR-6855-5p	MIMAT0027610	Homo_sapiens	0.0499245	1.45397	miRNA	miRBase	UUGGGGUUUGGGGUGCAGACAUUGC
100	hsa-miR-4690-5p	MIMAT0019779	Homo_sapiens	0.00701147	1.45734	miRNA	miRBase	GAGCAGGCGAGGCUGGGCUGAA
100	hsa-miR-6737-5p	MIMAT0027375	Homo_sapiens	0.0486871	1.46747	miRNA	miRBase	UUGGGGUGGUCGGCCCUGGAG
100	hsa-miR-3620-5p	MIMAT0022967	Homo_sapiens	0.0406369	1.47087	miRNA	miRBase	GUGGGCUGGGCUGGGCUGGGCC
100	hsa-miR-323b-5p	MIMAT0001630	Homo_sapiens	0.0419554	1.4811	miRNA	miRBase	AGGUUGUCCGUGGUGAGUUCGCA
100	hsa-miR-769-5p	MIMAT0003886	Homo_sapiens	0.00840249	1.4914	miRNA	miRBase	UGAGACCUCUGGGUUCUGAGCU
100	hsa-miR-6779-5p	MIMAT0027458	Homo_sapiens	0.00653608	1.49831	miRNA	miRBase	CUGGGAGGGGCUGGGUUUGGC
100	hsa-miR-4508	MIMAT0019045	Homo_sapiens	0.00335359	1.50873	miRNA	miRBase	GCGGGGCUGGGCGCGCG
100	hsa-miR-4750-5p	MIMAT0019887	Homo_sapiens	0.0086886	1.50873	miRNA	miRBase	CUCGGGCGGAGGUGGUUGAGUG
100	hsa-miR-6501-5p	MIMAT0025458	Homo_sapiens	0.0311269	1.51572	miRNA	miRBase	AGUUGCCAGGGCUGCCUUUGGU
100	hsa-miR-3195	MIMAT0015079	Homo_sapiens	0.0301089	1.52626	miRNA	miRBase	CGCGCCGGGCCCGGGUU
100	hsa-miR-4646-5p	MIMAT0019707	Homo_sapiens	0.0216612	1.52626	miRNA	miRBase	ACUGGGAAGAGGAGCUGAGGGA
100	hsa-miR-1236-5p	MIMAT0022945	Homo_sapiens	0.00789583	1.52979	miRNA	miRBase	UGAGUGACAGGGGAAAUGGGGA
100	hsa-miR-4800-3p	MIMAT0019979	Homo_sapiens	0.0313334	1.52979	miRNA	miRBase	CAUCCGUCCGUCUGUCCAC
100	hsa-miR-504-3p	MIMAT0026612	Homo_sapiens	0.00575332	1.54399	miRNA	miRBase	GGGAGUGCAGGGCAGGGUUUC
100	hsa-miR-4259	MIMAT0016880	Homo_sapiens	0.0342382	1.59107	miRNA	miRBase	CAGUUGGGUCUAGGGGUCAGGA
100	hsa-miR-1909-5p	MIMAT0007882	Homo_sapiens	0.023071	1.63203	miRNA	miRBase	UGAGUGCCGGUGCCUGCCCUG
100	hsa-miR-4467	MIMAT0018994	Homo_sapiens	0.0354501	1.63203	miRNA	miRBase	UGGCGGCGGUAGUUAUGGGCUU

100	hsa-miR-382-5p	MIMAT0000737	Homo_sapiens	0.00305457	1.63959	miRNA	miRBase	GAAGUUGUUCGUGGUGGAUUCG
100	hsa-miR-22-5p	MIMAT0004495	Homo_sapiens	0.0083791	1.65481	miRNA	miRBase	AGUUCUUCAGUGGCAAGCUUUA
100	hsa-miR-3178	MIMAT0015055	Homo_sapiens	0.00447069	1.65864	miRNA	miRBase	GGGGCGCGGCCGGAUCG
100	hsa-miR-7641	MIMAT0029782	Homo_sapiens	0.0212972	1.67018	miRNA	miRBase	UUGAUCUCGGAAGCUAAGC
100	hsa-miR-6782-5p	MIMAT0027464	Homo_sapiens	0.0361093	1.68179	miRNA	miRBase	UAGGGGUGGGGGAAUUCAGGGGUGU
100	hsa-miR-6759-5p	MIMAT0027418	Homo_sapiens	0.00543458	1.71317	miRNA	miRBase	UUGUGGGUGGGCAGAAGUCUGU
100	hsa-miR-4486	MIMAT0019020	Homo_sapiens	0.0385485	1.72907	miRNA	miRBase	GCUGGGCGAGGCUGGCA
100	hsa-miR-4732-5p	MIMAT0019855	Homo_sapiens	0.0279479	1.73708	miRNA	miRBase	UGUAGAGCAGGGAGCAGGAAGCU
100	hsa-miR-3689a-3p	MIMAT0018118	Homo_sapiens	0.0319095	1.86607	miRNA	miRBase	CUGGGAGGUGUGAUAUCGUGGU
100	hsa-miR-4492	MIMAT0019027	Homo_sapiens	0.000763139	1.88775	miRNA	miRBase	GGGGCUGGGCGCGCGCC
100	hsa-miR-664b-5p	MIMAT0022271	Homo_sapiens	0.0457466	1.88775	miRNA	miRBase	UGGGCUAAGGGAGAUGAUUGGGUA
100	hsa-miR-6772-5p	MIMAT0027444	Homo_sapiens	0.0068067	1.88775	miRNA	miRBase	UGGGUGUAGGCUGGAGCUGAGG
100	hsa-miR-628-3p	MIMAT0003297	Homo_sapiens	0.00313941	1.91853	miRNA	miRBase	UCUAGUAAGAGUGGCAGUCGA
100	hsa-miR-1587	MIMAT0019077	Homo_sapiens	0.0119977	1.94981	miRNA	miRBase	UUGGGCUGGGCUGGGUUGGG
100	hsa-miR-4485	MIMAT0019019	Homo_sapiens	0.0183008	1.97703	miRNA	miRBase	UAACGGCCGCGGUACCCUAA
100	hsa-miR-1299	MIMAT0005887	Homo_sapiens	0.00355229	2.04202	miRNA	miRBase	UUCUGGAAUUCUGUGUGAGGGA
100	hsa-miR-4710	MIMAT0019815	Homo_sapiens	0.0344819	2.06098	miRNA	miRBase	GGGUGAGGGCAGGUGGUU
100	hsa-miR-1247-3p	MIMAT0022721	Homo_sapiens	0.024787	2.08493	miRNA	miRBase	CCCCGGGAACGUCGAGACUGGAGC
100	hsa-miR-595	MIMAT0003263	Homo_sapiens	0.0167553	2.08493	miRNA	miRBase	GAAGUGUGCCGUGGUGUGUCU
100	hsa-miR-3135b	MIMAT0018985	Homo_sapiens	0.00111547	2.12383	miRNA	miRBase	GGCUGGAGCGAGUGCAGUGGUG
100	hsa-miR-6726-5p	MIMAT0027353	Homo_sapiens	0.0279663	2.12383	miRNA	miRBase	CGGGAGCUGGGGUCUGCAGGU
100	hsa-miR-574-5p	MIMAT0004795	Homo_sapiens	0.0494754	2.40605	miRNA	miRBase	UGAGUGUGUGUGUGUGAGUGUGU
100	hsa-miR-1306-3p	MIMAT0005950	Homo_sapiens	0.0474054	2.54324	miRNA	miRBase	ACGUUGGCUCUGGUGGUG
100	hsa-miR-5093	MIMAT0021085	Homo_sapiens	0.0466651	2.59668	miRNA	miRBase	AGGAAAUGAGGCUGGCUAGGAGC
100	hsa-miR-4717-3p	MIMAT0019830	Homo_sapiens	0.0110086	2.67586	miRNA	miRBase	ACACAUGGGUGGCUGUGGCCU
100	hsa-miR-1250-5p	MIMAT0005902	Homo_sapiens	0.0047967	2.69447	miRNA	miRBase	ACGGUGCUGGAUGUGGCCUUU
100	hsa-miR-4535	MIMAT0019075	Homo_sapiens	0.0183158	3.1456	miRNA	miRBase	GUGGACCUGGCUGGGAC
100	hsa-miR-4455	MIMAT0018977	Homo_sapiens	0.00915105	3.46615	miRNA	miRBase	AGGGUGUGUGUGUUUUU
100	hsa-miR-185-3p	MIMAT0004611	Homo_sapiens	0.0261356	3.79298	miRNA	miRBase	AGGGGCUGGCUUUCCUCUGGUC
100	hsa-miR-4284	MIMAT0016915	Homo_sapiens	0.0189302	3.89962	miRNA	miRBase	GGGCUCACAUCACCCCAU
100	hsa-miR-1261	MIMAT0005913	Homo_sapiens	0.0128907	4.26733	miRNA	miRBase	AUGGAUAAGGCUUUGGCUU
250	hsa-miR-4521	MIMAT0019058	Homo_sapiens	0.00370262	-4.29701	miRNA	miRBase	GCUAAGGAAGUCCUGUGCUCAG
250	hsa-miR-7977	MIMAT0031180	Homo_sapiens	0.010934	-4.14106	miRNA	miRBase	UUCCCAGCCAACGCACCA
250	hsa-miR-513b-5p	MIMAT0005788	Homo_sapiens	0.00758923	-3.93583	miRNA	miRBase	UUCACAAGGAGGUGUCAUUUAU
250	hsa-miR-223-3p	MIMAT0000280	Homo_sapiens	0.0388933	-3.5801	miRNA	miRBase	UGUCAGUUUGUCAAAUACCCCA
250	hsa-miR-27a-5p	MIMAT0004501	Homo_sapiens	0.00418236	-3.43426	miRNA	miRBase	AGGGCUUAGCUGCUUGUGAGCA
250	hsa-miR-23a-5p	MIMAT0004496	Homo_sapiens	0.00409178	-3.27918	miRNA	miRBase	GGGGUUCCUGGGGAUGGGAUUU
250	hsa-miR-4454	MIMAT0018976	Homo_sapiens	0.0159749	-2.9828	miRNA	miRBase	GGAUCCGAGUCACGGCACCA
250	hsa-miR-4669	MIMAT0019749	Homo_sapiens	0.014491	-2.96905	miRNA	miRBase	UGUGUCCGGGAAGUGGAGGAGG
250	hsa-miR-194-5p	MIMAT0000460	Homo_sapiens	0.0128401	-2.86129	miRNA	miRBase	UGUAACAGCAACUCCAUGUGGA
250	hsa-miR-192-5p	MIMAT0000222	Homo_sapiens	0.00162661	-2.85469	miRNA	miRBase	CUGACCUAUGAAUUGACAGCC
250	hsa-miR-454-3p	MIMAT0003885	Homo_sapiens	0.003595	-2.75745	miRNA	miRBase	UAGUGCAAUAUUGCUUAUAGGGU
250	hsa-miR-510-5p	MIMAT0002882	Homo_sapiens	0.0176947	-2.73208	miRNA	miRBase	UACUCAGGAGAGUGGCAAUCAC
250	hsa-miR-26b-5p	MIMAT0000083	Homo_sapiens	0.0191297	-2.71948	miRNA	miRBase	UUCAAGUAAUUCAGGAUAGGU
250	hsa-miR-21-5p	MIMAT0000076	Homo_sapiens	0.00106473	-2.71321	miRNA	miRBase	UAGCUUAUCAGACUGAUGUUGA
250	hsa-miR-15a-5p	MIMAT0000068	Homo_sapiens	0.00295857	-2.65737	miRNA	miRBase	UAGCAGCACAUAAUGGUUUGUG
250	hsa-miR-3911	MIMAT0018185	Homo_sapiens	0.000945662	-2.57279	miRNA	miRBase	UGUGUGGAUCCUGGAGGAGGCA
250	hsa-miR-27b-3p	MIMAT0000419	Homo_sapiens	0.00796694	-2.53151	miRNA	miRBase	UUCACAGUGGCUAAGUUCUGC
250	hsa-miR-421	MIMAT0003339	Homo_sapiens	0.0378511	-2.52567	miRNA	miRBase	AUCAACAGACAUUAAUUGGGCGC
250	hsa-miR-513a-5p	MIMAT0002877	Homo_sapiens	0.00485624	-2.52567	miRNA	miRBase	UUCACAGGGAGGUGUCAU
250	hsa-let-7f-5p	MIMAT0000067	Homo_sapiens	0.00963421	-2.39496	miRNA	miRBase	UGAGGUAGUAGAUUGUAUAGUU
250	hsa-miR-195-5p	MIMAT0000461	Homo_sapiens	0.0227582	-2.38943	miRNA	miRBase	UAGCAGCACAGAAAUAUUGGC
250	hsa-miR-128-3p	MIMAT0000424	Homo_sapiens	0.033601	-2.38392	miRNA	miRBase	UCACAGUGAACCGGUCUCUUU
250	hsa-miR-424-3p	MIMAT0004749	Homo_sapiens	0.00510722	-2.3511	miRNA	miRBase	CAAAACGUGAGGCGCUGCUAU
250	hsa-miR-92b-3p	MIMAT0003218	Homo_sapiens	0.0164732	-2.34567	miRNA	miRBase	UAUUGCACUCGUCCCGGCCUCC
250	hsa-miR-7162-3p	MIMAT0028235	Homo_sapiens	0.003432	-2.27626	miRNA	miRBase	UCUGAGGUGGAACAGCAGC
250	hsa-miR-30c-5p	MIMAT0000244	Homo_sapiens	0.000777445	-2.21914	miRNA	miRBase	UGUAAACAUCCUACACUCUCAGC
250	hsa-miR-27a-3p	MIMAT0000084	Homo_sapiens	0.000514566	-2.21402	miRNA	miRBase	UUCACAGUGGCUAAGUUCCGC
250	hsa-miR-503-5p	MIMAT0002874	Homo_sapiens	0.0407797	-2.20891	miRNA	miRBase	UAGCAGCGGGAACAGUUCUGCAG
250	hsa-miR-6880-5p	MIMAT0027660	Homo_sapiens	0.00141783	-2.19872	miRNA	miRBase	UGGUGGAGGAAGAGGGCAGCUC
250	hsa-miR-197-3p	MIMAT0000227	Homo_sapiens	0.00274739	-2.11404	miRNA	miRBase	UUCACCACCUUCUCCACCCAGC
250	hsa-miR-365b-5p	MIMAT0022833	Homo_sapiens	0.0368902	-2.10429	miRNA	miRBase	AGGGACUUUCAGGGGCAGCUGU
250	hsa-miR-23b-3p	MIMAT0000418	Homo_sapiens	0.00112927	-2.09943	miRNA	miRBase	AUCACAUUGCCAGGGAUUACC
250	hsa-miR-29b-3p	MIMAT0000100	Homo_sapiens	0.0065521	-2.05148	miRNA	miRBase	UAGCACCAUUUGAAAUCAGUGUU
250	hsa-miR-146b-5p	MIMAT0002809	Homo_sapiens	0.000625415	-2.01857	miRNA	miRBase	UGAGAACUGAAUUCCAUAGGCU
250	hsa-miR-34a-5p	MIMAT0000255	Homo_sapiens	0.0127188	-1.97247	miRNA	miRBase	UGGCAGUGUCUUAGCUGGUUGU
250	hsa-miR-29a-3p	MIMAT0000086	Homo_sapiens	0.00335034	-1.96791	miRNA	miRBase	UAGCACCAUCUGAAAUCGGUUA
250	hsa-let-7g-5p	MIMAT0000414	Homo_sapiens	0.00564593	-1.92297	miRNA	miRBase	UGAGGUAGUAGUUUGUACAGUU
250	hsa-let-7d-3p	MIMAT0004484	Homo_sapiens	0.0405721	-1.86607	miRNA	miRBase	CUAUACGACCUGCUGCCUUUCU
250	hsa-miR-374b-5p	MIMAT0004955	Homo_sapiens	0.00542493	-1.8025	miRNA	miRBase	AUAUAAUACAACCUGCUAAGUG
250	hsa-miR-148b-3p	MIMAT0000759	Homo_sapiens	0.0421696	-1.79834	miRNA	miRBase	UCAGUGCAUCACAGAACUUUGU
250	hsa-miR-19a-3p	MIMAT0000073	Homo_sapiens	0.00761486	-1.79419	miRNA	miRBase	UGUGCAAAUCUAUGCAAAACUGA
250	hsa-miR-20b-5p	MIMAT0001413	Homo_sapiens	0.0327554	-1.79419	miRNA	miRBase	CAAAGUGCUCAUAGUGCAGGUAG
250	hsa-miR-138-1-3p	MIMAT0004607	Homo_sapiens	0.0200261	-1.78592	miRNA	miRBase	GCUACUUCACAACACCAGGGCC
250	hsa-miR-6795-5p	MIMAT0027490	Homo_sapiens	0.0389695	-1.75727	miRNA	miRBase	UGGGGGGACAGGAUGAGAGGCUGU
250	hsa-miR-532-3p	MIMAT0004780	Homo_sapiens	0.0311045	-1.73708	miRNA	miRBase	CCUCCCACACCCAAGGCUUGCA
250	hsa-miR-15b-5p	MIMAT0000417	Homo_sapiens	0.0200631	-1.70527	miRNA	miRBase	UAGCAGCACAUCAUGGUUUACA
250	hsa-miR-23a-3p	MIMAT0000078	Homo_sapiens	0.000220263	-1.70527	miRNA	miRBase	AUCACAUUGCCAGGGAUUUCC
250	hsa-miR-28-5p	MIMAT0000085	Homo_sapiens	0.0253379	-1.68568	miRNA	miRBase	AAGGAGCUCACAGUCUAUUGAG
250	hsa-miR-22-3p	MIMAT0000077	Homo_sapiens	0.00316892	-1.68179	miRNA	miRBase	AAGCUGCCAGUUGAAGAACUGU
250	hsa-miR-3609	MIMAT0017986	Homo_sapiens	0.02524	-1.68179	miRNA	miRBase	CAAAGUGAUGAGUAAUACUGGCUG
250	hsa-miR-26a-5p	MIMAT0000082	Homo_sapiens	0.000135955	-1.64338	miRNA	miRBase	UUCAAGUAAUCCAGGAUAGGCU
250	hsa-miR-2115-5p	MIMAT0011158	Homo_sapiens	0.0353866	-1.63959	miRNA	miRBase	AGCUUCCAUGACUCCUGAUGGA
250	hsa-miR-25-3p	MIMAT0000081	Homo_sapiens	0.00287314	-1.6358	miRNA	miRBase	CAUUGCACUUGUCUCGGUCUGA
250	hsa-miR-19b-3p	MIMAT0000074	Homo_sapiens	0.0221838	-1.59107	miRNA	miRBase	UGUGCAAAUCCAUGCAAAACUGA
250	hsa-miR-3663-3p	MIMAT0018085	Homo_sapiens	0.0181492	-1.56555	miRNA	miRBase	UGAGCACCACACAGGCCGGGCGC
250	hsa-miR-7847-3p	MIMAT0030422	Homo_sapiens	0.000689079	-1.55473	miRNA	miRBase	CGUGGAGGACGAGGAGGAGGC
250	hsa-miR-3607-5p	MIMAT0017984	Homo_sapiens	0.0362765	-1.54399	miRNA	miRBase	GCAUGUGAUGAAGCAAAUCAGU
250	hsa-miR-6074	MIMAT0023699	Homo_sapiens	0.0282931	-1.54399	miRNA	miRBase	GAUAUUCAGAGGCUAGGUGG
250	hsa-miR-4298	MIMAT0016852	Homo_sapiens	0.0207467	-1.50873	miRNA	miRBase	CUGGGACAGGAGGAGGAGGCAG
250	hsa-miR-16-5p	MIMAT0000069	Homo_sapiens	0.00519187	-1.49485	miRNA	miRBase	UAGCAGCACGUAAAUAUUGGCG
250	hsa-miR-20a-5p	MIMAT0000075	Homo_sapiens	0.00925899	-1.49485	miRNA	miRBase	UAAAGUGCUUAUAGUGCAGGUAG
250	hsa-miR-146a-5p	MIMAT0000449	Homo_sapiens	0.0193865	-1.4811	miRNA	miRBase	UGAGAACUGAAUUCCAUGGGUU
250	hsa-let-7i-5p	MIMAT0000415	Homo_sapiens	0.00964129	-1.47087	miRNA	miRBase	UGAGGUAGUAGUUUGUGCUGUU
250	hsa-miR-423-3p	MIMAT0001340	Homo_sapiens	0.0307227	-1.47087	miRNA	miRBase	AGCUCGGUCUGAGGCCCCUCAGU
250	hsa-miR-142-5p	MIMAT0000433	Homo_sapiens	0.0107865	-1.44393	miRNA	miRBase	CAUAAAGUAGAAAGCACUACU
250	hsa-miR-514b-3p	MIMAT0015088	Homo_sapiens	0.0138683	-1.42076	miRNA	miRBase	AUUGACACCUCUGUGAGUGGA

250	hsa-miR-24-3p	MIMAT0000080	Homo_sapiens	0.00270097	-1.41748	miRNA	miRBase	UGGCUCAGUUCAGCAGGAACAG
250	hsa-miR-107	MIMAT0000104	Homo_sapiens	0.00244463	-1.41095	miRNA	miRBase	AGCAGCAUUGUACAGGGCUAUCA
250	hsa-miR-6882-5p	MIMAT0027664	Homo_sapiens	0.0142096	-1.39797	miRNA	miRBase	UACAAGUCAGGAGCUGAAGCAG
250	hsa-let-7a-5p	MIMAT0000062	Homo_sapiens	0.0410258	-1.38511	miRNA	miRBase	UGAGGUAGUAGGUUGUAUAGUU
250	hsa-miR-17-5p	MIMAT0000070	Homo_sapiens	0.00137981	-1.38511	miRNA	miRBase	CAAAGUGCUUACAGUGCAGGUAG
250	hsa-miR-191-5p	MIMAT0000440	Homo_sapiens	0.0188203	-1.37872	miRNA	miRBase	CAACGGAAUCCCAAAAGCAGCUG
250	hsa-miR-4317	MIMAT0016872	Homo_sapiens	0.00865922	-1.37237	miRNA	miRBase	ACAUUGCCAGGGAGUUU
250	hsa-miR-342-3p	MIMAT0000753	Homo_sapiens	0.0160483	-1.3566	miRNA	miRBase	UCUCACACAGAAAUCGCACCCGU
250	hsa-miR-3942-5p	MIMAT0018358	Homo_sapiens	0.0395785	-1.35347	miRNA	miRBase	AAGCAAUACUGUUACCUGAAAU
250	hsa-miR-103a-3p	MIMAT0000101	Homo_sapiens	0.00442585	-1.34723	miRNA	miRBase	AGCAGCAUUGUACAGGGCUAUGA
250	hsa-miR-2355-5p	MIMAT0016895	Homo_sapiens	0.00795528	-1.34723	miRNA	miRBase	AUCCCCAGAUACAAUGGACAA
250	hsa-miR-4797-5p	MIMAT0019972	Homo_sapiens	0.0211061	-1.34723	miRNA	miRBase	GACAGAGUGCCACUUACUGAA
250	hsa-miR-23c	MIMAT0018000	Homo_sapiens	0.00298585	-1.33176	miRNA	miRBase	AUCACAUUGCCAGUGAUUACCC
250	hsa-miR-6740-5p	MIMAT0027381	Homo_sapiens	0.0376125	-1.32562	miRNA	miRBase	AGUUUGGGAUGGAGAGAGGAGA
250	hsa-miR-106a-5p	MIMAT0000103	Homo_sapiens	0.00979143	-1.31039	miRNA	miRBase	AAAAGUGCUUACAGUGCAGGUAG
250	hsa-miR-342-5p	MIMAT0004694	Homo_sapiens	0.0111126	-1.30737	miRNA	miRBase	AGGGGUGCUAUCUGUGAUUGA
250	hsa-miR-7109-5p	MIMAT0028115	Homo_sapiens	0.0464736	-1.30737	miRNA	miRBase	CUGGGGGGAGGAGACCCUGCU
250	hsa-miR-3945	MIMAT0018361	Homo_sapiens	0.0382098	-1.30134	miRNA	miRBase	AGGGCAUAGGAGAGGGUUGAUAU
250	hsa-miR-3136-5p	MIMAT0015003	Homo_sapiens	0.030952	-1.27751	miRNA	miRBase	CUGACUGAAUAGGUAGGGUCAUU
250	hsa-miR-5681a	MIMAT0022469	Homo_sapiens	0.0342972	-1.27751	miRNA	miRBase	AGAAAGGGUGGCAAUACCUCUU
250	hsa-miR-1304-5p	MIMAT0005892	Homo_sapiens	0.0131391	-1.25411	miRNA	miRBase	UUUGAGGCUACAGUGAGAUGUG
250	hsa-miR-105-3p	MIMAT0004516	Homo_sapiens	0.0208316	-1.20581	miRNA	miRBase	ACGGAUGUUUGAGCAUGUGCUA
250	hsa-miR-92a-3p	MIMAT0000092	Homo_sapiens	0.00621384	-1.19748	miRNA	miRBase	UAUUGCACUUGUCCCGGCCUGU
250	hsa-miR-152-3p	MIMAT0000438	Homo_sapiens	0.0051494	-1.19196	miRNA	miRBase	UCAGUGCAUGACAGAACUUGG
250	hsa-miR-548y	MIMAT0018354	Homo_sapiens	0.0496539	-1.14605	miRNA	miRBase	AAAAGUAAUCACUGUUUUUGCC
250	hsa-miR-4745-5p	MIMAT0019878	Homo_sapiens	0.0494844	1.12506	miRNA	miRBase	UGAGUGGGGCUCCCGGGACGGCG
250	hsa-miR-1469	MIMAT0007347	Homo_sapiens	0.0440744	1.14076	miRNA	miRBase	CUCGGCGCGGGGCGCGGGCUCC
250	hsa-miR-6090	MIMAT0023715	Homo_sapiens	0.0256245	1.17013	miRNA	miRBase	GGGGAGCGAGGGGCGGGGC
250	hsa-miR-3665	MIMAT0018087	Homo_sapiens	0.0266305	1.18099	miRNA	miRBase	AGCAGGUGCGGGGCGGCG
250	hsa-miR-6089	MIMAT0023714	Homo_sapiens	0.00862132	1.18099	miRNA	miRBase	GGAGGCCGGGGUGGGGCGGGGCGG
250	hsa-miR-4466	MIMAT0018993	Homo_sapiens	0.0269221	1.18372	miRNA	miRBase	GGGUGCGGGCCGGCGGGG
250	hsa-miR-6786-5p	MIMAT0027472	Homo_sapiens	0.0314176	1.18921	miRNA	miRBase	GCGGUGGGGCCGGAGGGGCGU
250	hsa-miR-6765-5p	MIMAT0027430	Homo_sapiens	0.0356743	1.19472	miRNA	miRBase	GUGAGGCGGGGCCAGGAGGGUGUGU
250	hsa-miR-6125	MIMAT0024598	Homo_sapiens	0.00383293	1.20581	miRNA	miRBase	GCGGAAGGCGGAGCGGCGGA
250	hsa-miR-7161-5p	MIMAT0028232	Homo_sapiens	0.00773703	1.20581	miRNA	miRBase	UAAAGACUGUAGAGGCAACUGGU
250	hsa-miR-4707-5p	MIMAT0019807	Homo_sapiens	0.0158665	1.21982	miRNA	miRBase	GCCCCGGCGCGGGCGGGUUCUGG
250	hsa-miR-33b-5p	MIMAT0003301	Homo_sapiens	0.0168636	1.2283	miRNA	miRBase	GUGCAUUGCUGUUGCAUUGC
250	hsa-miR-3529-5p	MIMAT0019828	Homo_sapiens	0.0423497	1.23114	miRNA	miRBase	AGGUAGACUGGGAUUUGUUGUU
250	hsa-miR-6134	MIMAT0024618	Homo_sapiens	0.0449742	1.24833	miRNA	miRBase	UGAGGUGGUAGGAUGUAGA
250	hsa-miR-640	MIMAT0003310	Homo_sapiens	0.0114513	1.24833	miRNA	miRBase	AUGAUCCAGGAACCUGCCUCU
250	hsa-miR-6087	MIMAT0023712	Homo_sapiens	0.00513652	1.25122	miRNA	miRBase	UGAGGCGGGGGGGCGAGC
250	hsa-miR-638	MIMAT0003308	Homo_sapiens	0.00148791	1.25122	miRNA	miRBase	AGGGAUCGCGGGCGGGUGGCGGCCU
250	hsa-miR-4433-3p	MIMAT0018949	Homo_sapiens	0.0383565	1.25411	miRNA	miRBase	ACAGGAGUGGGGGUGGGACAU
250	hsa-miR-208b-5p	MIMAT0026722	Homo_sapiens	0.00391218	1.26868	miRNA	miRBase	AAGCUUUUUGCUCGAAUUAUGU
250	hsa-miR-3173-3p	MIMAT0015048	Homo_sapiens	0.0211431	1.26868	miRNA	miRBase	AAAGGAGGAAAUAGGCAGGCCA
250	hsa-miR-3940-5p	MIMAT0019229	Homo_sapiens	0.00367144	1.27162	miRNA	miRBase	GUGGGUUGGGGCGGGCUCUG
250	hsa-miR-3960	MIMAT0019337	Homo_sapiens	0.007742	1.27162	miRNA	miRBase	GGCGGCGGCGGAGGCGGGGG
250	hsa-miR-6789-5p	MIMAT0027478	Homo_sapiens	0.0245962	1.27162	miRNA	miRBase	GUAGGGGCGUCCCGGGCGCGCGGG
250	hsa-miR-4690-5p	MIMAT0019779	Homo_sapiens	0.0424354	1.30134	miRNA	miRBase	GAGCAGGCGAGGCUGGGCUGAA
250	hsa-miR-5787	MIMAT0023252	Homo_sapiens	0.00344486	1.31039	miRNA	miRBase	GGGCUGGGGCGCGGGGAGGU
250	hsa-miR-6821-5p	MIMAT0027542	Homo_sapiens	0.0426688	1.31342	miRNA	miRBase	GUGCGUGGUGGCUCGAGGCGGGG
250	hsa-miR-1343-5p	MIMAT0027038	Homo_sapiens	0.0303363	1.32256	miRNA	miRBase	UGGGGAGCGGCCCCCGGGUGGG
250	hsa-miR-3656	MIMAT0018076	Homo_sapiens	0.00159476	1.32562	miRNA	miRBase	GGCGGGUGCGGGGGUGG
250	hsa-miR-4695-5p	MIMAT0019788	Homo_sapiens	0.0116799	1.32869	miRNA	miRBase	CAGGAGGCAGUGGGCGAGCAGG
250	hsa-miR-4741	MIMAT0019871	Homo_sapiens	0.00342267	1.32869	miRNA	miRBase	CGGGCUGUCCGGAGGGGUCGGCU
250	hsa-miR-6818-5p	MIMAT0027536	Homo_sapiens	0.0384806	1.33176	miRNA	miRBase	UUGUGUGAGUACAGAGAGCAUC
250	hsa-miR-762	MIMAT0010313	Homo_sapiens	0.00252308	1.33176	miRNA	miRBase	GGGGCUGGGGCCGGGGCCGAGC
250	hsa-miR-6724-5p	MIMAT0025856	Homo_sapiens	0.0016813	1.33484	miRNA	miRBase	CUGGGCCCGCGGCGGGCGUGGGG
250	hsa-miR-4687-3p	MIMAT0019775	Homo_sapiens	0.0327663	1.34723	miRNA	miRBase	UGGCUGUUGGAGGGGGCAGGC
250	hsa-miR-7108-5p	MIMAT0028113	Homo_sapiens	0.0000541668	1.34723	miRNA	miRBase	GUGUGGCCGGCAGGCGGGUGG
250	hsa-miR-6857-5p	MIMAT0027614	Homo_sapiens	0.0440462	1.3566	miRNA	miRBase	UUGGGGAUUGGGUCAGGCCAGU
250	hsa-miR-4763-3p	MIMAT0019913	Homo_sapiens	0.00527847	1.35974	miRNA	miRBase	AGGCAGGGGCUGGUGCUGGGCGGG
250	hsa-miR-1236-5p	MIMAT0022945	Homo_sapiens	0.0365986	1.3692	miRNA	miRBase	UGAGUGACAGGGGAAAUGGGGA
250	hsa-miR-92b-5p	MIMAT0004792	Homo_sapiens	0.0206061	1.37237	miRNA	miRBase	AGGGACGGGACGCGGUGCAGUG
250	hsa-miR-4632-5p	MIMAT0022977	Homo_sapiens	0.0235594	1.37554	miRNA	miRBase	GAGGGCAGCGUGGGUGUGGCGGA
250	hsa-miR-6752-5p	MIMAT0027404	Homo_sapiens	0.018558	1.37554	miRNA	miRBase	GGGGGGUGUGGAGCCAGGGGGC
250	hsa-miR-4692	MIMAT0019783	Homo_sapiens	0.0344624	1.37872	miRNA	miRBase	UCAGGCAGUGUGGGUAUCAGAU
250	hsa-miR-6756-5p	MIMAT0027412	Homo_sapiens	0.00366404	1.38511	miRNA	miRBase	AGGGUGGGGCUGGAGGUGGGGCU
250	hsa-miR-6779-5p	MIMAT0027458	Homo_sapiens	0.0179486	1.4012	miRNA	miRBase	CUGGGAGGGGCUGGGUUUGGC
250	hsa-miR-665	MIMAT0004952	Homo_sapiens	0.0215885	1.40444	miRNA	miRBase	ACCAGGAGGCUGAGGCCCCU
250	hsa-miR-149-3p	MIMAT0004609	Homo_sapiens	0.0032008	1.40769	miRNA	miRBase	AGGGAGGGACGGGGGCUGUGC
250	hsa-miR-2861	MIMAT0013802	Homo_sapiens	0.000108342	1.41095	miRNA	miRBase	GGGGCCUGGCGGUGGGCGG
250	hsa-miR-4270	MIMAT0016900	Homo_sapiens	0.0434003	1.41095	miRNA	miRBase	UCAGGGAGUCAGGGGAGGGC
250	hsa-miR-6798-5p	MIMAT0027496	Homo_sapiens	0.0400342	1.41421	miRNA	miRBase	CCAGGGGGAUGGGCGAGCUUGGG
250	hsa-miR-6794-5p	MIMAT0027488	Homo_sapiens	0.00621589	1.42405	miRNA	miRBase	CAGGGGGACUGGGGGUGAGC
250	hsa-miR-1268b	MIMAT0018925	Homo_sapiens	0.0318672	1.42734	miRNA	miRBase	CGGGCGUGGUGGUGGGGGUG
250	hsa-miR-6858-5p	MIMAT0027616	Homo_sapiens	0.0022221	1.42734	miRNA	miRBase	GUGAGGAGGGGCUGGCAGGGAC
250	hsa-miR-6791-5p	MIMAT0027482	Homo_sapiens	0.000145124	1.43065	miRNA	miRBase	CCCCUGGGGCUGGGCAGGCGGA
250	hsa-miR-1227-5p	MIMAT0022941	Homo_sapiens	0.0016755	1.43396	miRNA	miRBase	GUGGGGCCAGGCGGUGG
250	hsa-miR-6722-3p	MIMAT0025854	Homo_sapiens	0.00216906	1.45397	miRNA	miRBase	UGCAGGGGUCGGGUGGGCCAGG
250	hsa-miR-939-5p	MIMAT0004982	Homo_sapiens	0.0303726	1.46409	miRNA	miRBase	UGGGGAGCUGAGGCUCUGGGGGUG
250	hsa-miR-3185	MIMAT0015065	Homo_sapiens	0.00418763	1.4811	miRNA	miRBase	AGAAGAAGGCGGUCGGUCUGCGG
250	hsa-miR-4433b-3p	MIMAT0030414	Homo_sapiens	0.00340585	1.48796	miRNA	miRBase	CAGGAGUGGGGGGUGGGACGU
250	hsa-miR-4651	MIMAT0019715	Homo_sapiens	0.00735428	1.49485	miRNA	miRBase	CGGGGUGGGUGAGGUCGGGC
250	hsa-miR-5001-5p	MIMAT0021021	Homo_sapiens	0.000275455	1.50177	miRNA	miRBase	AGGGCUGGACUCAGCGGCGGAGCU
250	hsa-miR-4758-5p	MIMAT0019903	Homo_sapiens	0.00414364	1.50525	miRNA	miRBase	GUGAGUGGGAGCCGGUGGGGCUG
250	hsa-miR-3178	MIMAT0015055	Homo_sapiens	0.0150106	1.50873	miRNA	miRBase	GGGGCGCGGCCGGAUCG
250	hsa-miR-6132	MIMAT0024616	Homo_sapiens	0.0252389	1.52274	miRNA	miRBase	AGCAGGGCUGGGGAUUGCA
250	hsa-miR-6732-5p	MIMAT0027365	Homo_sapiens	0.0127075	1.52626	miRNA	miRBase	UAGGGGGUGGCAGGCUGGCC
250	hsa-miR-6889-5p	MIMAT0027678	Homo_sapiens	0.0477804	1.54756	miRNA	miRBase	UCGGGGAGUCUGGGGUCCGGAAU
250	hsa-miR-4259	MIMAT0016880	Homo_sapiens	0.0349872	1.5874	miRNA	miRBase	CAGUUGGGUCUAGGGGUCAGGA
250	hsa-miR-3651	MIMAT0018071	Homo_sapiens	0.0443327	1.59475	miRNA	miRBase	CAUAGCCCGGUCGCUGGUACAUGA
250	hsa-miR-382-5p	MIMAT0000737	Homo_sapiens	0.00446883	1.59475	miRNA	miRBase	GAAGUUGUUCGUGGUGGAUUCG
250	hsa-miR-6803-5p	MIMAT0027506	Homo_sapiens	0.000585287	1.60956	miRNA	miRBase	CUGGGGGUGGGGGGCUGGGCGU
250	hsa-miR-6772-5p	MIMAT0027444	Homo_sapiens	0.0277805	1.62826	miRNA	miRBase	UGGGUGUAGGCUGGAGCUGAGG
250	hsa-miR-4711-3p	MIMAT0019817	Homo_sapiens	0.0211802	1.6358	miRNA	miRBase	CGUGUCUUCUGGCUUGAU
250	hsa-miR-4463	MIMAT0018987	Homo_sapiens	0.00369961	1.63959	miRNA	miRBase	GAGACUGGGGUGGGGCC

250	hsa-miR-4655-5p	MIMAT0019721	Homo_sapiens	0.0333557	1.64338	miRNA	miRBase	CACCGGGGAUGGCAGAGGGUCG
250	hsa-miR-937-5p	MIMAT0022938	Homo_sapiens	0.00418059	1.65864	miRNA	miRBase	GUGAGUCAGGGUGGGGCUGG
250	hsa-miR-6824-5p	MIMAT0027548	Homo_sapiens	0.0280743	1.67018	miRNA	miRBase	GUAGGGGAGGUUGGGCCAGGGA
250	hsa-miR-6743-5p	MIMAT0027387	Homo_sapiens	0.0000049477	1.67404	miRNA	miRBase	AAGGGGCAGGGACGGGUGGCCC
250	hsa-miR-4508	MIMAT0019045	Homo_sapiens	0.000581439	1.68568	miRNA	miRBase	GCGGGGCUGGGCGCGCG
250	hsa-miR-6827-5p	MIMAT0027554	Homo_sapiens	0.0497049	1.70921	miRNA	miRBase	UGGGAGCCAUGAGGGUCUGUGC
250	hsa-miR-4725-3p	MIMAT0019844	Homo_sapiens	0.0149591	1.72508	miRNA	miRBase	UGGGGAAGGCGUCAGUGUCGGG
250	hsa-miR-4505	MIMAT0019041	Homo_sapiens	0.000387897	1.80667	miRNA	miRBase	AGGCUGGGCUGGGACGGA
250	hsa-miR-6870-5p	MIMAT0027640	Homo_sapiens	0.0245061	1.81924	miRNA	miRBase	UGGGGGAGAUGGGGGUUGA
250	hsa-miR-4484	MIMAT0019018	Homo_sapiens	0.0338122	1.90088	miRNA	miRBase	AAAAGGCGGGAGAAGCCCCA
250	hsa-miR-4507	MIMAT0019044	Homo_sapiens	0.0069198	1.90528	miRNA	miRBase	CUGGGUUGGGCUGGGCUGGG
250	hsa-miR-3620-5p	MIMAT0022967	Homo_sapiens	0.00186001	1.94981	miRNA	miRBase	GUGGGCUGGGCUGGGCUGGGCC
250	hsa-miR-7845-5p	MIMAT0030420	Homo_sapiens	0.00127578	2.03261	miRNA	miRBase	AAGGGACAGGGAGGGUCGUGG
250	hsa-miR-1587	MIMAT0019077	Homo_sapiens	0.00789776	2.05148	miRNA	miRBase	UUGGGCUGGGCUGGGUUGGG
250	hsa-miR-1229-5p	MIMAT0022942	Homo_sapiens	0.0205574	2.12874	miRNA	miRBase	GUGGGUAGGGUUUGGGGGAGAGCG
250	hsa-miR-4485	MIMAT0019019	Homo_sapiens	0.010384	2.13366	miRNA	miRBase	UAACGGCCGCGGUACCCUAA
250	hsa-miR-4701-3p	MIMAT0019799	Homo_sapiens	0.0384218	2.13366	miRNA	miRBase	AUGGGUGAUGGGUGUGGUGU
250	hsa-miR-4492	MIMAT0019027	Homo_sapiens	0.000160397	2.15348	miRNA	miRBase	GGGGCUGGGCGCGCGCC
250	hsa-miR-572	MIMAT0003237	Homo_sapiens	0.0257722	2.20381	miRNA	miRBase	GUCCGCUCGGCGGUGGCCCA
250	hsa-miR-4530	MIMAT0019069	Homo_sapiens	0.0000840265	2.21914	miRNA	miRBase	CCCAGCAGGACGGGAGCG
250	hsa-miR-5196-5p	MIMAT0021128	Homo_sapiens	0.0161374	2.22427	miRNA	miRBase	AGGGAAGGGGACGAGGGUUGGG
250	hsa-miR-4647	MIMAT0019709	Homo_sapiens	0.0222122	2.35653	miRNA	miRBase	GAAGAUGGUGCUGUGCUGAGGAA
250	hsa-miR-4472	MIMAT0018999	Homo_sapiens	0.0455119	2.43401	miRNA	miRBase	GGUGGGGGGUGUUGUUUU
250	hsa-miR-7641	MIMAT0029782	Homo_sapiens	0.000388426	2.56685	miRNA	miRBase	UUGAUCUCGGAAGCUAAGC
250	hsa-miR-3135b	MIMAT0018985	Homo_sapiens	0.000165706	2.59068	miRNA	miRBase	GGCUGGAGCGAGUGCAGUGGUG
250	hsa-miR-4462	MIMAT0018986	Homo_sapiens	0.0276486	2.75745	miRNA	miRBase	UGACACGGAGGGUGGCUUGGGAA
