# Supplementary material for: The DCMU Herbicide Shapes T-cell Functions By Modulating Micro-RNA Expression Profiles
Source: Front Immunol. 2022 Jul 28;13:925241. doi: 10.3389/fimmu.2022.925241 (PMC9366666; doi:10.3389/fimmu.2022.925241)
Supplement: Supplementary file 2 [file DataSheet_2.pdf]

Supplemental Table 2. KEGG pathways enriched in the target genes of DEmiRNAs.

DCMU concentration	Pathway ID	Pathway	Fold Enrichment	-LOG10(pval)	pval	Up_regulated
10μM	hsa03015	mRNA surveillance pathway	24.264550	1.69	2.0e-02	CPSF6, CPSF7, PPP1CB, MSI2, ETF1, SMG1, PPP2R1B, PPP2R2B, PPP2R3A, HBS1L
10μM	hsa04010	MAPK signaling pathway	7.361943	7.20	6.3e-08	PRKCA, PRKCB, PPP3CA, PPP3R1, RAPGEF2, NF1, IGF1, IGF2, VEGFA, FGFR1, NTRK2, IGF1R, SOS2, NRAS, TNF, RAC1, GADD45A, PAK2, MAP3K13, TAOK1, RPS6KA5, ATF2, MAX, MEF2C, PTPN7, DUSP5, DUSP3, CHUK
10μM	hsa04012	ErbB signaling pathway	23.122689	4.54	2.9e-05	CAMK2A, CAMK2D, PRKCA, PRKCB, CBL, PAK2, HBEGF, SOS2, NRAS, GAB1, RPS6KB1
10μM	hsa04014	Ras signaling pathway	8.838656	7.48	3.3e-08	IGF1, IGF2, VEGFA, FGFR1, NTRK2, IGF1R, GAB1, PTPN11, SOS2, GNB1, GNB2, NRAS, NF1, RAC1, PAK2, CHUK, FOXO4, ETS1, ETS2, RGL1, PRKCA, PRKCB, RAB5B
10μM	hsa04015	Rap1 signaling pathway	5.620098	1.72	1.9e-02	ADCY2, IGF1, VEGFA, FGFR1, IGF1R, MAGI2, RAPGEF2, RAPGEF6, PRKCA, PRKCB, CNR1, RAC1, TLN2, NRAS
10μM	hsa04020	Calcium signaling pathway	18.014590	4.89	1.3e-05	ATP2B4, ATP2B2, ADCY2, PLN, STIM1, RYR2, GRM5, HTR2C, CAMK2A, CAMK2D, PPP3CA, PPP3R1, PRKCA, PRKCB
10μM	hsa04062	Chemokine signaling pathway	9.243638	3.52	3.0e-04	CCL20, CCR9, JAK2, ADCY2, SOS2, NRAS, CHUK, ITK, VAV3, RAC1, GNB1, GNB2, PRKCB, GRK6
10μM	hsa04064	NF-kappa B signaling pathway	18.737487	1.79	1.6e-02	PRKCB, BCL10, TNF, CHUK, CSNK2A1, CSNK2A2
10μM	hsa04066	HIF-1 signaling pathway	13.721410	5.34	4.6e-06	IGF1, IGF1R, RPS6KB1, HIF1A, EGLN1, PRKCA, PRKCB, CAMK2A, CAMK2D, VEGFA, HK1
10μM	hsa04150	mTOR signaling pathway	19.988792	5.48	3.3e-06	ATP6V1A, LAMTOR1, RRAGB, RPS6KB1, WNT5B, FZD4, TNF, IGF1, IGF1R, SOS2, NRAS, CHUK, RICTOR, PRKCA, PRKCB
10μM	hsa04151	PI3K-Akt signaling pathway	9.019668	4.82	1.5e-05	IGF1, IGF2, VEGFA, FGFR1, NTRK2, IGF1R, SOS2, NRAS, RAC1, PRLR, JAK2, COL4A4, COL4A1, LAMC1, FN1, ITGA6, ITGA10, ITGAV, GNB1, GNB2, RPS6KB1, PRKCA, MAGI2, PPP2R1B, PPP2R2B, PPP2R3A, PHLPP1, YWHAB, ATF2, CHUK
10μM	hsa04152	AMPK signaling pathway	16.233628	3.42	3.8e-04	LEP, LEPR, FBP1, PPARGC1A, ELAVL1, FOXO1, SCD, PPP2R1B, PPP2R2B, PPP2R3A, RAB10, RAB14, IGF1, IGF1R, RPS6KB1
10μM	hsa04218	Cellular senescence	13.990238	2.21	6.1e-03	FOXO1, NRAS, LIN54, BTRC, HIPK1, PPP1CB, ETS1, GADD45A, ZFP36L1, PPP3CA, PPP3R1, NFATC2
10μM	hsa04310	Wnt signaling pathway	9.494824	1.96	1.1e-02	WNT5B, FZD4, CSNK2A1, CSNK2A2, TCF7L2, BTRC, VANGL2, VANGL1, PRICKLE2, RAC1, CAMK2A, CAMK2D, PPP3CA, PPP3R1, PRKCA, PRKCB, NFATC2
10μM	hsa04350	TGF-beta signaling pathway	11.465000	2.20	6.3e-03	LTBP1, INHBB, BMPR2, ACVR1, TNF, PPP2R1B, RPS6KB1
10μM	hsa04370	VEGF signaling pathway	22.591133	4.28	5.2e-05	VEGFA, PRKCA, PRKCB, NRAS, PPP3CA, PPP3R1, NFATC2, RAC1
10μM	hsa04620	Toll-like receptor signaling pathway	16.349376	1.64	2.3e-02	RAC1, TOLLIP, FADD, CHUK, TNF
10μM	hsa04650	Natural killer cell mediated cytotoxicity	13.065527	3.46	3.5e-04	PTPN11, VAV3, RAC1, TNF, SOS2, NRAS, PPP3CA, PPP3R1, NFATC2, PRKCA, PRKCB
10μM	hsa04659	Th17 cell differentiation	15.732418	1.59	2.6e-02	IRF4, JAK2, RORA, HIF1A, AHR, HLA-DQA1, PPP3CA, PPP3R1, NFATC2, CHUK, RARA
**10μM**	**hsa04660**	**T cell receptor signaling pathway**	**13.235209**	**2.89**	**1.3e-03**	**ITK, VAV3, PAK2, PPP3CA, PPP3R1, NFATC2, SOS2, NRAS, BCL10, CHUK, TNF**
10μM	hsa04662	B cell receptor signaling pathway	17.331822	2.37	4.2e-03	VAV3, RAC1, PPP3CA, PPP3R1, NFATC2, SOS2, NRAS, PRKCB, BCL10, CHUK
10μM	hsa04670	Leukocyte transendothelial migration	10.110229	1.42	3.8e-02	CLDN1, OCLN, RAC1, PTPN11, PRKCA, PRKCB, MYL12B, ITK, VAV3
10μM	hsa04750	Inflammatory mediator regulation of TRP channels	23.960293	3.24	5.7e-04	HTR2C, PRKCE, PPP1CB, CAMK2A, CAMK2D, ADCY2, PRKCA, PRKCB, IGF1
10μM	hsa04810	Regulation of actin cytoskeleton	11.240196	4.24	5.7e-05	FGFR1, FN1, ITGA6, ITGA10, ITGAV, SOS2, NRAS, VAV3, ARHGEF12, RAC1, PAK2, PPP1CB, MYL12B, PIP4K2A, CFL2, SSH1, SSH3, SSH2, MYH10
10μM	hsa04918	Thyroid hormone synthesis	17.752258	3.19	6.4e-04	CGA, ADCY2, PRKCA, PRKCB, ATF2, ATP1B2, LRP2
10μM	hsa04919	Thyroid hormone signaling pathway	12.478912	2.54	2.9e-03	ITGAV, NRAS, NCOA1, HIF1A, PLN, PRKCA, PRKCB, ATP1B2, FOXO1
10μM	hsa05170	Human immunodeficiency virus 1 infection	7.086882	2.54	2.9e-03	BTRC, RPS6KB1, NRAS, PPP3CA, PPP3R1, NFATC2, PRKCA, PRKCB, GNB1, GNB2, RAC1, PAK2, CFL2, TNF, CHUK, FADD, WEE2
10μM	hsa05200	Pathways in cancer	4.786469	5.55	2.8e-06	TCF7L2, WNT5B, FZD4, ARHGEF12, ADCY2, GNB1, GNB2, COL4A4, COL4A1, LAMC1, FN1, ITGA6, ITGAV, NKX3-1, CHUK, RPS6KB1, FOXO1, CBL, PRKCA, PRKCB, JAK2, VEGFA, IGF1, IGF2, IGF1R, FGFR1, SOS2, NRAS, ETS1, RPS6KA5, CCDC6, CAMK2A, CAMK2D, RAC1, NCOA1, RARA, RUNX1T1, MAX, GADD45A, FADD, EGLN1, HIF1A, DLL1
10μM	hsa05205	Proteoglycans in cancer	10.395725	8.33	4.7e-09	NRAS, RAC1, ANK3, GAB1, PPP1CB, ARHGEF12, CAMK2A, CAMK2D, IGF1, IGF1R, RPS6KB1, TNF, HIF1A, VEGFA, CBL, TIMP3, ITGAV, FN1, FZD4, FGFR1, PTPN11, SOS2, PRKCA, PRKCB, HBEGF, IGF2, WNT5B
100μM	hsa04658	Th1 and Th2 cell differentiation	16.017510	1.32	4.8e-02	RBPJ, NFKB1, JAK1, PPP3CA, PPP3R1, NFATC3, IKBKB, MAPK8, NOTCH1, NOTCH2
100μM	hsa04115	p53 signaling pathway	19.132026	1.66	4.8e-02	CDK6, CCNE2, GADD45A, ZMAT3, SIAH1, IGFBP3, IGF1, RRM2B, SESN3, PTEN, TP73
100μM	hsa04621	NOD-like receptor signaling pathway	13.363769	1.69	2.0e-02	IKBKB, NFKB1, MAPK8, TRAF3, ANTXR1, TRPM7, PLCB1, ITPR1, MCU, DNM1L, NAMPT, NEK7, NAIP, RHOA, IFNAR2, JAK1
100μM	hsa03018	RNA degradation	19.799784	2.39	4.0e-03	CNOT6L, CNOT1, TOB1, TOB2, BTG1, BTG2, PABPC5, DCP2, DDX6, PATL1, XRN1, XRN2, PNPT1, PFKM, HSPD1
100μM	hsa04750	Inflammatory mediator regulation of TRP channels	14.636000	2.44	4.5e-02	HTR2C, GNAQ, PLCB1, ITPR1, PRKCE, ASIC1, CAMK2D, IL1RAP, MAPK8, PIK3R3, PRKACA, PRKACB, TRPV4, PRKCA, PRKCB, IGF1
100μM	hsa03013	RNA transport	13.987894	2.49	3.2e-03	EEF1A1, XPO1, NUP50, NUP153, SUMO2, EIF4G1, EIF4E2, EIF4B, PABPC5, PAIP1, FMR1, PNN
100μM	hsa04370	VEGF signaling pathway	21.119769	2.77	1.7e-03	PRKCA, PRKCB, RAF1, PPP3CA, PPP3R1, PXN, PIK3R3, RAC1, AKT3
100μM	hsa04650	Natural killer cell mediated cytotoxicity	12.088732	2.96	2.1e-03	VAV3, VAV2, RAC1, PIK3R3, SHC4, SOS2, RAF1, PPP3CA, PPP3R1, PRKCA, PRKCB, SH2D1B, IFNAR2
100μM	hsa04721	Synaptic vesicle cycle	30.239669	3.41	3.9e-04	SLC17A6, SYT1, VAMP2, STXBP1, UNC13A, SNAP25, CPLX2, CPLX3, DNM1, CLTC, ATP6V0E2
100μM	hsa04657	IL-17 signaling pathway	21.376826	3.54	2.9e-04	NFKB1, TRAF3, IKBKB, MAPK8, USP25, ELAVL1, GSK3B
100μM	hsa03015	mRNA surveillance pathway	24.339246	4.04	9.2e-05	PNN, RNMT, CPSF6, CPSF7, PAPOLA, CSTF2T, PABPC5, ETF1, GSPT1, SMG1, SMG7, PPP2R2B, PPP2R3A, PPP2R5D, PPP2R5E
100μM	hsa04152	AMPK signaling pathway	18.459820	4.30	5.0e-05	CAB39, ADIPOR1, PRKAA1, PRKAA2, PRKAB2, PFKFB2, PFKM, CREB5, ELAVL1, SCD, PPP2R2B, PPP2R3A, PPP2R5D, PPP2R5E, IGF1, IGF1R, IRS1, PIK3R3, PDPK1, AKT3, RPS6KB1
100μM	hsa05170	Human immunodeficiency virus 1 infection	10.163889	4.41	9.1e-05	BTRC, GNAI2, GNAO1, PIK3R3, PXN, AKT3, RPS6KB1, NFKB1, RAF1, GNAQ, ITPR1, PPP3CA, PPP3R1, NFATC3, PRKCA, PRKCB, GNG12, RAC1, PAK4, CFL2, TNFRSF1B, IKBKB, MAPK8, TRIM5, AP1G1, AP1S1, CUL5, CUL4A
100μM	hsa04210	Apoptosis	14.519841	4.43	3.7e-05	ITPR1, EIF2AK3, DAB2IP, MAPK8, IKBKB, NFKB1, GADD45A, CASP2, PIK3R3, PDPK1, AKT3, RAF1
100μM	hsa04668	TNF signaling pathway	18.587083	4.44	3.6e-05	MAPK8, ITCH, IKBKB, NFKB1, CREB5, DNM1L, CSF1, TNFRSF1B, TRAF3, PIK3R3, AKT3, DAB2IP, IRF1
100μM	hsa04670	Leukocyte transendothelial migration	15.930340	4.49	4.1e-03	CLDN18, EZR, PIK3R3, RAC1, CTNND1, PRKCA, PRKCB, PXN, ARHGAP35, RHOA, ROCK1, GNAI2, RAPGEF3, ITK, VAV3, VAV2
100μM	hsa04064	NF-kappa B signaling pathway	25.871717	5.41	3.9e-06	PRKCB, BCL10, TRAF3, IKBKB, ERC1, NFKB1
100μM	hsa04630	JAK-STAT signaling pathway	20.760284	5.46	3.5e-06	IL6R, IL22RA1, IL6ST, IFNAR2, PRLR, EGFR, PDGFRB, JAK1, STAT3, SOCS5, SOCS6, PIM1, EP300, PIAS2, SOS2, RAF1, PIK3R3, AKT3
**100μM**	**hsa04660**	**T cell receptor signaling pathway**	**19.563576**	**5.45**	**3.5e-06**	**ITK, NCK2, VAV3, VAV2, PAK4, RHOA, DLG1, PPP3CA, PPP3R1, NFATC3, SOS2, RAF1, BCL10, IKBKB, NFKB1, PIK3R3, PDPK1, AKT3, GSK3B**
100μM	hsa04062	Chemokine signaling pathway	12.429724	5.66	2.2e-05	CCL22, STAT3, GNAI2, PRKACA, PRKACB, SHC4, SOS2, RAF1, PIK3R3, AKT3, IKBKB, NFKB1, GSK3A, GSK3B, ITK, VAV3, VAV2, RAC1, WASL, RHOA, ROCK1, GNG12, PXN, PLCB1, PRKCB
100μM	hsa04218	Cellular senescence	15.104231	6.03	2.3e-05	TGFBR1, SMAD2, SMAD3, CDK6, RB1, RBL1, PIK3R3, CCNE2, AKT3, PTEN, LIN9, LIN54, BTRC, HIPK3, HIPK1, RAF1, ETS1, GADD45A, GATA4, NFKB1, IGFBP3, TRPV4, TRPM7, PPP3CA, PPP3R1, NFATC3, ITPR1, SLC25A5, MCU

100µM	hsa04015	Rap1 signaling pathway	13.551852	6.24	5.8e-07	GRIN2A, F2R, RAPGEF3, GNAQ, PLCB1, FGF23, IGF1, CSF1, EGFR, FGFR1, FGFR2, NGFR, IGF1R, PDGFRB, FLT1, MAGI3, RAPGEF6, PRKCA, PRKCB, PRKD3, CNR1, GNAI2, GNAO1, SIPA1L2, SIPA1L3, RALGDS, RALA, RALB, RAC1, TLN2, RHOA, PRKCI, CTNND1, RAF1, PIK3R3, AKT3, VAV2
100µM	hsa04662	B cell receptor signaling pathway	26.935358	6.68	2.1e-07	VAV3, VAV2, RAC1, PPP3CA, PPP3R1, NFATC3, SOS2, RAF1, PRKCB, BCL10, IKBKB, NFKB1, PIK3R3, AKT3, GSK3B
100µM	hsa04010	MAPK signaling pathway	16.048246	7.35	1.2e-06	CACNA1C, CACNB1, PRKACA, PRKACB, PRKCA, PRKCB, GNG12, PPP3CA, PPP3R1, NF1, RASA1, TGFA, FGF23, IGF1, IGF2, CSF1, EGFR, FGFR1, FGFR2, NGFR, NTRK2, IGF1R, PDGFRB, FLT1, SOS2, RAF1, LAMTOR3, RPS6KA2, ELK1, ELK4, SRF, MAPT, IL1RAP, TGFBR1, RAC1, GADD45A, STK4, MAP3K1, MAP3K2, TAOK1, MAPK8, CDC25B, NFATC3, MAX, MEF2C, AKT3, PPM1A, DUSP8, DUSP3, PPM1B, IKBKB, NLK, NFKB1
100µM	hsa04066	HIF-1 signaling pathway	21.211594	7.58	2.5e-06	IL6R, STAT3, NFKB1, IGF1, EGFR, IGF1R, PIK3R3, AKT3, EIF4E2, RPS6KB1, HIF1A, EGLN1, EGLN3, ARNT, EP300, PRKCA, PRKCB, CAMK2D, FLT1, CDKN1B
100µM	hsa05206	MicroRNAs in cancer	27.293240	7.68	1.8e-06	PTEN, CDKN1B, TIMP3, CDK6, CDC25B, CCNE2, PIM1, EGFR, EZR, EP300, ZEB1, ABL1, TP63, DDIT4, PAK4, SLC7A1, STAT3, APC2, IRS1, HNRNPK, RECK, NOTCH1, NOTCH2, CD44, DICER1, FZD3, RHOA, ZFPM2, IGF2BP1, NFKB1, IKBKB, PRKCA, PRKCB, RAF1, SOS2, SHC4, PDGFRB, BMPR2, ROCK1, PRKCE, HDAC4
100µM	hsa04919	Thyroid hormone signaling pathway	26.855046	7.69	2.0e-08	PRKACA, PRKACB, ITGAV, RAF1, NCOA1, NCOA3, EP300, MED4, MED12L, MED13, GATA4, HIF1A, NOTCH1, NOTCH2, PLCB1, PRKCA, PRKCB, SLC9A1, ATP1A2, ATP1B4, ATP1B2, SLC16A2, PIK3R3, PDPK1, AKT3, GSK3B, PFKFB2, RCAN2
100µM	hsa04151	PI3K-Akt signaling pathway	13.939048	8	3.2e-08	TGFA, FGF23, IGF1, IGF2, CSF1, EGFR, FGFR1, FGFR2, NGFR, NTRK2, IGF1R, PDGFRB, FLT1, SOS2, RAF1, IRS1, RAC1, PRLR, IL6R, IFNAR2, JAK1, COL4A1, LAMC1, FN1, ITGA6, ITGAV, PIK3R3, F2R, GNG12, PDPK1, PRKAA1, PRKAA2, DDIT4, EIF4E2, RPS6KB1, EIF4B, PRKCA, AKT3, PTEN, PPP2R2B, PPP2R3A, PPP2R5D, PPP2R5E, GSK3B, CDKN1B, CDK6, CCNE2, YWHAZ, YWHAQ, YWHAG, CREB5, IKBKB, NFKB1
100µM	hsa04510	Focal adhesion	14.252285	8.15	3.8e-07	COL4A1, LAMC1, FN1, ITGA6, ITGAV, IGF1, PDGFRB, IGF1R, EGFR, FLT1, ARHGAP35, RHOA, DIAPH1, ROCK1, PPP1R12A, TLN2, PXN, PDPK1, AKT3, GSK3B, PRKCA, PRKCB, PIK3R3, PTEN, VAV3, VAV2, RAC1, PAK4, MAPK8, CAV2, SHC4, SOS2, RAF1, ELK1
100µM	hsa04014	Ras signaling pathway	20.327778	8.21	6.2e-09	TGFA, FGF23, IGF1, IGF2, CSF1, EGFR, FGFR1, FGFR2, NGFR, NTRK2, IGF1R, PDGFRB, FLT1, GAB1, SHC4, SOS2, GNG12, PRKACA, PRKACB, GRIN2A, NF1, RASA1, STK4, RAC1, PAK4, RHOA, PIK3R3, AKT3, IKBKB, NFKB1, SHOC2, RAF1, ELK1, ETS1, ETS2, RALGDS, RGL1, RALA, RALB, MAPK8, PRKCA, PRKCB, ABL1, RAB5B
100µM	hsa04659	Th17 cell differentiation	21.466133	8.69	2.5e-08	IL1RAP, IRF4, TGFBR1, SMAD2, SMAD3, SMAD4, JAK1, IL6R, IL6ST, STAT3, RORA, HIF1A, AHR, PPP3CA, PPP3R1, NFATC3, IKBKB, NFKB1, MAPK8, RARA
100µM	hsa04810	Regulation of actin cytoskeleton	14.379228	9	7.7e-08	F2R, FGF23, EGFR, FGFR1, FGFR2, PDGFRB, FN1, ITGA6, ITGAV, GNG12, SOS2, PIK3R3, VAV3, VAV2, RAF1, ARHGEF12, ARHGAP35, RHOA, RAC1, PAK4, ROCK1, PPP1R12A, DIAPH1, SLC9A1, PIP4K2A, PIKFYVE, WASL, WASF2, NCKAP1, WASF1, PXN, EZR, CFL2, SSH2, IQGAP1, IQGAP3, APC2, MYH10
100µM	hsa04310	Wnt signaling pathway	20.458925	9.89	2.3e-10	WNT5A, SFRP1, FZD7, FZD3, FZD4, CXXC4, GSK3B, APC2, LEF1, EP300, SMAD4, NLK, PPARD, PRKACA, PRKACB, SIAH1, TBL1X, BTRC, VANGL2, PRICKLE2, DAAM1, RHOA, RAC1, MAPK8, PLCB1, CAMK2D, PPP3CA, PPP3R1, PRKCA, PRKCB, NFATC3, SMAD3
100µM	hsa04350	TGF-beta signaling pathway	29.803036	9.96	1.1e-10	NOG, LTBP1, BMPR2, TGFBR1, ACVR1B, ACVR1C, SMAD5, SMAD2, SMAD3, SMAD4, SMAD7, SMURF1, RBL1, EP300, SP1, RHOA, ROCK1, RPS6KB1
100µM	hsa04110	Cell cycle	29.325319	10.64	2.6e-11	CDK6, RB1, RBL1, ABL1, TFDP2, GSK3B, SMAD2, SMAD3, SMAD4, CDKN1B, CCNE2, CDC25B, YWHAZ, YWHAQ, YWHAG, STAG2, STAG1, RAD21, TTK, CDC14B, CDC14A, EP300, GADD45A, ORC4
100µM	hsa05205	Proteoglycans in cancer	14.767856	10.72	5.0e-10	CD44, RAF1, RAC1, IQGAP1, ELK1, RHOA, ROCK1, ANK3, GAB1, PIK3R3, AKT3, SLC9A1, PPP1R12A, ARHGEF12, ITPR1, CAMK2D, STAT3, IGF1, IGF1R, PDPK1, RPS6KB1, EIF4B, EGFR, CAV2, HIF1A, CBL, TIMP3, SDC1, ITGAV, FN1, FZD7, FZD3, FZD4, EZR, FGFR1, FRS2, SOS2, PRKCA, PRKCB, PXN, HBEGF, IGF2, WNT5A, PRKACA, PRKACB, SMAD2, VAV2
100µM	hsa05166	Human T-cell leukemia virus 1 infection	16.107117	10.89	9.0e-08	NRP1, TLN2, XPO1, PPP3CA, PPP3R1, NFATC3, JAK1, SLC25A5, RB1, WNT5A, FZD7, FZD3, FZD4, GSK3B, APC2, DLG1, PDGFRB, PIK3R3, AKT3, MAP3K1, IKBKB, NFKB1, NFYB, SRF, ELK4, ELK1, ETS1, ETS2, PRKACA, PRKACB, CREM, ATF1, ATF3, CRTC1, EP300, TGFBR1, SMAD2, SMAD3, SMAD4, TP53INP1, MAPK8
100µM	hsa05200	Pathways in cancer	10.772427	16.52	3.0e-15	APPL1, APC2, GSK3B, LEF1, WNT5A, FZD7, FZD3, FZD4, F2R, ARHGEF12, RHOA, ROCK1, GNAI2, PRKACA, PRKACB, GNG12, COL4A1, LAMC1, FN1, ITGA6, ITGAV, PIK3R3, PTEN, AKT3, IKBKB, NFKB1, TRAF3, RPS6KB1, CDKN1B, ABL1, CBL, PIM1, GNAQ, PLCB1, PRKCA, PRKCB, IL6R, IL6ST, IFNAR2, JAK1, STAT3, TGFA, EGFR, PDGFRB, IGF1, IGF2, IGF1R, FGF23, FGFR1, FGFR2, SOS2, RAF1, ETS1, CCDC6, STK4, ELK1, CAMK2D, RALGDS, RALA, RALB, RAC1, MAPK8, NCOA1, NCOA3, SP1, PPARD, PML, RARA, MAX, CDK6, CCNE2, RB1, GADD45A, TGFBR1, SMAD2, SMAD3, SMAD4, EGLN1, EGLN3, HIF1A, ARNT, ARNT2, EP300, NOTCH1, NOTCH2, AR
250µM	hsa03013	RNA transport	17.115269	9.74	1.1e-07	RPP14, XPO5, EEF1A1, XPO1, CLNS1A, TGS1, NUP50, NUP153, SEH1L, NUP133, NUP54, NUP188, NUP35, NUP210, NUP214, RANGAP1, UBE2I, SUMO3, SUMO2, SUMO1, GEMIN5, EIF3J, EIF1AY, EIF1AX, EIF5, EIF2S2, EIF4G3, EIF4G1, EIF4G2, EIF4E2, EIF4A2, EIF4B, PABPC5, EIF4EBP2, FMR1, FXR1, FXR2, MAGOHB, CASC3, ACIN1, THOC2, NXT2, UPF2
250µM	hsa03015	mRNA surveillance pathway	15.815375	3.74	4.6e-03	MAGOHB, CASC3, ACIN1, NXT2, RNGTT, RNMT, CPSF6, CPSF7, PAPOLG, PAPOLA, CLP1, WDR33, WDR82, PPP1CB, PPP1CC, CSTF2T, DAZAP1, MSI1, MSI2, PABPC5, ETF1, GSPT1, UPF2, SMG1, SMG7, SMG5, SMG6, PPP2R2A, PPP2R3C, PPP2R3A, PPP2R5D, PPP2R5E, HBS1L
250µM	hsa03018	RNA degradation	14.035290	3.15	4.0e-02	EXOSC2, TTC37, CNOT6L, CNOT1, CNOT2, CNOT3, CNOT4, CNOT7, CNOT8, PARN, TOB1, TOB2, BTG3, BTG1, BTG2, PABPC5, PAN3, DCP2, DDX6, EDC3, PATL1, XRN1, ENO1, PFKM, PFKP
250µM	hsa04010	MAPK signaling pathway	21.119769	29.69	2.0e-30	CACNA1A, CACNA1B, CACNA1C, CACNA1D, CACNA1E, CACNA1G, CACNA2D1, CACNA2D2, CACNB1, CACNB2, CACNB3, CACNB4, PRKACA, PRKACB, PRKACG, PRKCA, PRKCB, PRKCG, GNG12, PPP3CA, PPP3CB, PPP3R1, PPP3R2, RASGRF2, RASGRP1, RASGRP3, RASGRP4, RAPGEF2, NF1, RASA1, RAP1A, RAP1B, TGFA, EREG, FGF1, FGF2, FGF17, FGF9, FGF5, FGF18, FGF19, FGF23, NTF3, IGF1, IGF2, CSF1, VEGFA, VEGFC, HGF, EFNA1, EFNA3, EFNA5, EGFR, ERBB3, FGFR1, FGFR2, FGFR3, NGFR, NTRK2, INSR, IGF1R, PDGFRA, PDGFRB, CSF1R, FLT3, FLT1, FLT4, MET, GRB2, SOS1, SOS2, KRAS, NRAS, RRAS, RRAS2, MRAS, ARAF, RAF1, LAMTOR3, MAPK1, MKNK2, RPS6KA2, RPS6KA6, ELK1, ELK4, MYC, SRF, MAPT, STMN1, PLA2G4E, TNF, TGFB2, TNFRSF1A, IL1RAP, TGFBR1, TGFBR2, FAS, RAC2, CDC42, CASP3, MYD88, IRAK1, GADD45A, TAB2, MAP4K3, MAP4K4, PAK2, STK4, MAP3K1, MAP3K2, MAP3K3, MAP3K13, MAP3K12, TAOK2, TAOK3, TAOK1, MAP2K4, MAP2K7, MAP2K3, MAPK8IP1, MAPK8IP3, FLNA, FLNB, ARRB1, MAPK8, MAPK10, MAPK9, MAPK13, MAPK14, MAPKAPK2, MAPKAPK3, RPS6KA5, RPS6KA4, CDC25B, JUND, ATF2, MAX, MEF2C, AKT1, AKT2, AKT3, PPM1A, PTPRR, PTPN7, DUSP1, DUSP4, DUSP2, DUSP7, DUSP8, DUSP5, DUSP16, DUSP9, DUSP10, DUSP3, PPM1B, HSPA8, MECOM, NR4A1, CHUK, IKBKB, NLK, NFKB1
250µM	hsa04012	ErbB signaling pathway	36.875787	22.89	1.3e-23	TGFA, EGFR, PLCG2, CAMK2A, CAMK2D, CAMK2G, PRKCA, PRKCB, PRKCG, CBL, CBLB, STAT5B, SRC, PTK2, ABL1, ABL2, PAK2, PAK3, PAK4, PAK6, MAP2K4, MAP2K7, MAPK8, MAPK10, MAPK9, ELK1, HBEGF, EREG, ERBB3, GRB2, SOS1, SOS2, KRAS, NRAS, ARAF, RAF1, MAPK1, MYC, GAB1, PIK3CA, PIK3CD, PIK3CB, PIK3R2, PIK3R3, AKT1, AKT2, AKT3, RPS6KB1, GSK3B, CDKN1B, CDKN1A
250µM	hsa04014	Ras signaling pathway	23.437793	24.03	8.9e-16	TGFA, FGF1, FGF2, FGF17, FGF9, FGF5, FGF18, FGF19, FGF23, NTF3, IGF1, IGF2, CSF1, VEGFA, VEGFC, HGF, EFNA1, EFNA3, EFNA5, EGFR, FGFR1, FGFR2, FGFR3, NGFR, NTRK2, INSR, IGF1R, PDGFRA, PDGFRB, CSF1R, FLT3, FLT1, FLT4, MET, GRB2, GAB1, GAB2, PTPN11, SOS1, SOS2, PLCG2, RASGRP1, RASGRP3, RASGRP4, GNB2, GNB4, GNG2, GNG4, GNG5, GNG7, GNG12, GNG13, PRKACA, PRKACB, PRKACG, RASGRF2, GRIN2A, CALM2, CALM1, KRAS, NRAS, MRAS, RRAS, RRAS2, NF1, RASA1, RASA4, SYNGAP1, STK4, TIAM1, RAC2, PAK2, PAK3, PAK4, PAK6, PIK3CA, PIK3CD, PIK3CB, PIK3R2, PIK3R3, AKT1, AKT2, AKT3, CHUK, IKBKB, NFKB1, BCL2L1, FOXO4, SHOC2, RAF1, MAPK1, PLA2G2D, PLA2G4E, ELK1, ETS1, BRAP, KSR1, KSR2, RAPGEF5, RAP1A, RAP1B, RALGDS, RGL1, RGL2, RALA, RALB, MAPK8, MAPK10, MAPK9, RALBP1, CDC42, PRKCA, PRKCB, PRKCG, ABL1, ABL2, RAB5A, RAB5B, ARF6
250µM	hsa04015	Rap1 signaling pathway	15.665443	15.11	7.8e-16	RAP1A, RAP1B, MRAS, RAPGEF5, GRIN2A, CALM2, CALM1, LPAR1, LPAR3, LPAR4, ADORA2B, ADCY2, ADCY3, RAPGEF3, RAPGEF4, GNAQ, PLCB1, PLCB2, PLCB4, RASGRP3, FGF1, FGF2, FGF17, FGF9, FGF5, FGF18, FGF19, FGF23, IGF1, CSF1, VEGFA, VEGFC, HGF, EFNA1, EFNA3, EFNA5, EGFR, FGFR1, FGFR2, FGFR3, NGFR, INSR, IGF1R, PDGFRA, PDGFRB, CSF1R, FLT1, FLT4, MET, RAPGEF1, CDH1, MAGI2, MAGI3, RAPGEF2, RAPGEF6, PRKCA, PRKCB, PRKCG, PRKD3, CNR1, GNAI1, GNAI2, GNAO1, RAP1GAP, SIPA1L2, SIPA1L3, RALGDS, RALA, RALB, RAC2, TLN1, TLN2, PFN1, VASP, ACTB, ACTG1, SRC, CDC42, TIAM1, PARD3, PARD6B, PRKCI, CTNND1, KRIT1, RGS14, ITGB1, RAF1, MAPK1, MAP2K3, MAPK13, MAPK14, PIK3CA, PIK3CD, PIK3CB, PIK3R2, PIK3R3, AKT1, AKT2, AKT3, KRAS, NRAS, RRAS, VAV2

250μM	hsa04060	Cytokine-cytokine receptor interaction	5.913535	2.34	1.0e-02	CCL22, CCL7, CXCL5, CXCL13, CXCL16, CX3CL1, IL15, TSLP, IFNA4, IFNA17, IL1RN, CSF1, TNF, TNFSF10, EDA, TNFSF12, TNFSF8, TGFB2, INHBB, GDF6, BMP7, ACKR4, CCR7, IL2RB, IL2RG, IL7R, IL9R, CSF2RB, IL13RA1, PRLR, CSF3R, IL6R, IL6ST, IL12RB1, CNTFR, OSMR, IL22RA1, IFNLR1, IFNAR2, IL1RAP, CSF1R, TNFRSF1A, TNFRSF1B, LTBR, TNFRSF14, FAS, TNFRSF21, EDAR, EDA2R, NGFR, TNFRSF11B, TNFRSF11A, TNFRSF12A, RELT, TGFBR1, TGFBR2, ACVRL1, ACVR2A, BMPR2, ACVR2B, ACVR1B, ACVR1C, ACVR1
250μM	hsa04062	Chemokine signaling pathway	9.139503	11.18	5.8e-09	CXCL5, CXCL13, CXCL16, CX3CL1, CCL7, CCL22, CCR7, JAK3, STAT2, STAT3, STAT5B, GNAI1, GNAI2, ADCY2, ADCY3, PRKACA, PRKACB, PRKACG, SRC, GRB2, SOS1, SOS2, KRAS, NRAS, RAF1, MAPK1, PIK3CA, PIK3CD, PIK3CB, PIK3R2, PIK3R3, AKT1, AKT2, AKT3, CHUK, IKBKB, NFKB1, GSK3A, GSK3B, ITK, VAV3, VAV2, RAC2, CDC42, WASL, GNB2, GNB4, GNG2, GNG4, GNG5, GNG7, GNG12, GNG13, ELMO1, PTK2, PXN, PLCB1, PLCB2, PLCB4, RAP1A, RAP1B, PARD3, TIAM1, PRKCB, PRKCD, GRK5, GRK6, ARRB1
250μM	hsa04064	NF-kappa B signaling pathway	23.993443	17.44	2.2e-17	PRKCQ, PLCG2, PRKCB, MYD88, IRAK1, TNF, TNFRSF1A, BIRC2, TRAF3, TNFRSF11A, LTBR, TAB2, TAB3, CHUK, IKBKB, UBE2I, ERC1, NFKB1, BCL2L1, TRAF1, TNFAIP3, PTGS2, CSNK2A1
250μM	hsa04066	HIF-1 signaling pathway	14.037084	13.10	1.2e-12	IL6R, STAT3, NFKB1, IGF1, INSR, EGFR, IGF1R, MAPK1, MKNK2, PIK3CA, PIK3CD, PIK3CB, PIK3R2, PIK3R3, AKT1, AKT2, AKT3, EIF4E2, RPS6KB1, HIF1A, CUL2, EGLN1, EGLN3, CREBBP, EP300, PLCG2, PRKCA, PRKCB, PRKCG, CAMK2A, CAMK2D, CAMK2G, LTBR, VEGFA, FLT1, EDN1, SLC2A1, PDK1, HK1, ENO1, PFKFB3, CDKN1A, CDKN1B, PDHA1, LDHA
250μM	hsa04110	Cell cycle	15.289939	23.85	1.4e-20	CCND1, CCND2, CDK6, RB1, ABL1, HDAC1, HDAC2, E2F1, E2F5, TFDP1, TFDP2, GSK3B, TGFB2, SMAD2, SMAD3, MYC, CDKN2D, CDKN1B, CDKN1A, CCNE1, CCNE2, SKP2, CCNA2, CDC25B, YWHAZ, YWHAB, YWHAQ, YWHAE, YWHAH, YWHAG, WEE1, WEE2, CCNH, CDC27, CDC23, STAG2, STAG1, CDC14B, CDC14A, CHEK1, CREBBP, EP300, GADD45A, SFN, CDC25A, ORC5
250μM	hsa04115	p53 signaling pathway	20.768318	8.72	2.6e-09	CHEK1, GORAB, MDM4, CDKN1A, CCND1, CCND2, CDK6, CCNE1, CCNE2, SFN, GADD45A, FAS, SIVA1, BCL2L1, EI24, ZMAT3, SIAH1, APAF1, CASP9, CASP3, IGFBP3, IGF1, CD82, RRM2, SESN1, SESN3, SESN2, PTEN, STEAP3, CCNG1, CCNG2, TP73
250μM	hsa04142	Lysosome	9.187696	2.85	2.1e-03	ATP6V0A1, CTSD, GLA, ARSB, GNS, SGSH, LIPA, ACP2, ASAH1, PSAP, GM2A, LAMP1, LAMP2, SLC11A2, LAPTM4B, LAPTM5, LAPTM4A, ABCA2, ABCB9, CD164, SORT1, MFSD8, SUMF1, GNPTAB, CLTB, CLTC, AP1G1, AP1S1, AP3M1, GGA2, GGA3, GGA1, LITAF
250μM	hsa04150	mTOR signaling pathway	14.650651	15.08	2.9e-14	SLC7A5, ATP6V1A, ATP6V1B2, ATP6V1C1, ATP6V1E1, LAMTOR1, LAMTOR3, FNIP1, FNIP2, RRAGD, AKT1S1, DEPTOR, MLST8, CLIP1, GRB10, ULK1, ULK2, EIF4E2, RPS6KB1, EIF4B, SKP2, DEPDC5, MIOS, SEH1L, SESN2, STRADB, CAB39, PRKAA1, PRKAA2, TSC1, DDIT4, WNT2, WNT2B, WNT3, WNT3A, WNT4, WNT5B, WNT7B, FZD7, FZD3, FZD4, FZD5, FZD6, LRP6, DVL3, GSK3B, TNF, TNFRSF1A, IKBKB, IGF1, INSR, IGF1R, GRB2, SOS1, SOS2, KRAS, NRAS, RAF1, MAPK1, RPS6KA2, RPS6KA6, IRS1, PIK3R2, PIK3R3, PIK3CA, PIK3CD, PIK3CB, PTEN, PDPK1, AKT1, AKT2, AKT3, CHUK, MAPKAP1, RICTOR, PRR5, PRKCA, PRKCB, PRKCG, SGK1
250μM	hsa04151	PI3K-Akt signaling pathway	11.509305	17.49	1.3e-17	TGFA, EREG, FGF1, FGF2, FGF17, FGF9, FGF5, FGF18, FGF19, FGF23, NTF3, IGF1, IGF2, CSF1, VEGFA, VEGFC, HGF, EFNA1, EFNA3, EFNA5, EGFR, ERBB3, FGFR1, FGFR2, FGFR3, NGFR, NTRK2, INSR, IGF1R, PDGFRA, PDGFRB, CSF1R, FLT3, FLT1, FLT4, MET, GRB2, SOS1, SOS2, KRAS, NRAS, RAF1, MAPK1, IRS1, IFNA4, IFNA17, PRLR, OSMR, IL2RB, IL2RG, IL6R, IL7R, IFNAR2, CSF3R, JAK3, COL1A1, COL1A2, COL2A1, COL4A2, COL4A1, COL4A5, COL4A3, COL6A1, COL9A2, LAMA4, LAMC1, RELN, THBS2, THBS3, ITGA2, ITGA3, ITGA5, ITGA6, ITGA7, ITGA11, ITGAV, ITGB1, ITGB6, ITGB8, PTK2, PIK3CA, PIK3CD, PIK3CB, PIK3R2, PIK3R3, CHRM1, CHRM2, LPAR1, LPAR3, LPAR4, LPAR6, GNB2, GNB4, GNG2, GNG4, GNG5, GNG7, GNG12, GNG13, PDPK1, PRKAA1, PRKAA2, DDIT4, TSC1, MLST8, EIF4E2, RPS6KB1, EIF4B, PRKCA, PKN2, SGK1, AKT1, AKT2, AKT3, MAGI2, PTEN, PPP2R2A, PPP2R3C, PPP2R3A, PPP2R5D, PPP2R5E, CRTC2, PHLPP1, PHLPP2, GSK3B, GYS1, MYC, CCND1, CDKN1A, CDKN1B, CDK6, CCND2, CCNE1, CCNE2, BCL2L11, YWHAZ, YWHAB, YWHAQ, YWHAE, YWHAH, YWHAG, BCL2L1, CASP9, CREB1, ATF2, CREB3L1, CREB3L2, NR4A1, CHUK, IKBKB, NFKB1
250μM	hsa04152	AMPK signaling pathway	17.687981	7.72	2.8e-07	CAB39, STRADB, CAMKK2, ADIPOQ, ADIPOR2, PRKAA1, PRKAA2, PRKAB2, PFKFB2, PFKFB3, PFKFB4, FBP1, PFKM, PFKP, HNF4A, CRTC2, CREB1, CREB3L1, CREB3L2, PPARGC1A, ELAVL1, CCND1, CCNA2, EEF2K, SIRT1, SLC2A4, GYS1, HMGCR, FASN, SCD, PPP2R2A, PPP2R3C, PPP2R3A, PPP2R5D, PPP2R5E, RAB10, RAB11B, RAB14, IGF1, IGF1R, INSR, IRS1, IRS2, PIK3CA, PIK3CD, PIK3CB, PIK3R2, PIK3R3, PDPK1, AKT1, AKT2, AKT3, TSC1, AKT1S1, RPS6KB1, PPARG, ULK1
250μM	hsa04210	Apoptosis	11.588894	8.82	3.2e-08	TNFSF10, FAS, TNF, TNFRSF1A, CASP3, CASP7, APAF1, CASP9, TUBA4A, ACTB, ACTG1, SPTAN1, PARP3, ITPR1, CAPN1, EIF2AK3, CTSD, BIRC2, BCL2L11, BCL2L1, DAB2IP, MAPK8, MAPK10, MAPK9, CHUK, IKBKB, NFKB1, PTPN13, GADD45A, TRAF1, CASP2, CSF2RB, PIK3CA, PIK3CD, PIK3CB, PIK3R2, PIK3R3, PDPK1, AKT1, AKT2, AKT3, KRAS, NRAS, RAF1, MAPK1
250μM	hsa04218	Cellular senescence	13.662696	14.79	9.8e-14	TGFB2, TGFBR1, TGFBR2, SMAD2, SMAD3, CDK6, CCND1, CCND2, RB1, E2F1, E2F5, PIK3CA, PIK3CD, PIK3CB, PIK3R2, PIK3R3, CDKN1A, CCNE1, CCNE2, KRAS, NRAS, RRAS, RRAS2, MRAS, AKT1, AKT2, AKT3, TSC1, PTEN, SIRT1, CCNA2, MYBL2, LIN9, LIN54, FOXM1, MYC, BTRC, FBXW11, HIPK3, HIPK1, PPP1CB, PPP1CC, RAF1, MAPK1, ETS1, MAP2K3, MAPK13, MAPK14, GADD45A, RAD9A, HUS1, CHEK1, CDC25A, NFKB1, IGFBP3, MAPKAPK2, ZFP36L1, ZFP36L2, CACNA1D, TRPV4, TRPM7, CAPN1, CALM2, CALM1, PPP3CA, PPP3CB, PPP3R1, PPP3R2, NFATC2, ITPR1, SLC25A5, MCU
250μM	hsa04310	Wnt signaling pathway	17.064905	18.15	4.9e-12	WNT2, WNT2B, WNT3, WNT3A, WNT4, WNT5B, WNT7B, SFRP1, SFRP2, SFRP5, FZD7, FZD3, FZD4, FZD5, FZD6, LRP6, DVL3, FRAT2, CSNK2A1, GSK3B, AXIN2, APC, APC2, CSNK1A1, TCF7, TCF7L1, TCF7L2, LEF1, CHD8, CREBBP, EP300, NLK, MYC, CCND1, CCND2, PPARD, PRKACA, PRKACB, PRKACG, SIAH1, TBL1X, TBL1XR1, BTRC, FBXW11, VANGL2, VANGL1, PRICKLE2, DAAM1, DAAM2, RAC2, MAPK8, MAPK10, MAPK9, PLCB1, PLCB2, PLCB4, CAMK2A, CAMK2D, CAMK2G, PPP3CA, PPP3CB, PPP3R1, PPP3R2, PRKCA, PRKCB, PRKCG, NFATC2, SMAD3
250μM	hsa04330	Notch signaling pathway	40.319559	13.07	8.4e-14	DLL1, JAG1, NOTCH1, NOTCH2, RBPJ, DVL3, NUMB, DTX3L, DTX1, DTX3, DTX4, NCSTN, APH1A, MAML2, MAML1, CREBBP, EP300, KAT2B, SNW1, NCOR2, HDAC1, HDAC2
250μM	hsa04350	TGF-beta signaling pathway	22.005346	15.32	7.7e-12	CHRD, NOG, NBL1, BMP7, GDF6, LTBP1, TGFB2, INHBB, BMPR2, TGFBR2, ACVR2A, ACVR2B, ACVR1, TGFBR1, ACVR1B, ACVR1C, SMAD5, SMAD2, SMAD3, SMAD7, SMURF1, ZFYVE9, ID2, E2F5, TFDP1, CREBBP, EP300, SP1, TGIF1, TGIF2, MYC, PITX2, MAPK1, TNF, RPS6KB1
250μM	hsa04370	VEGF signaling pathway	35.697561	11.21	5.5e-10	VEGFA, PLCG2, PRKCA, PRKCB, PRKCG, KRAS, NRAS, RAF1, MAPK1, PLA2G4E, PPP3CA, PPP3CB, PPP3R1, PPP3R2, NFATC2, PTGS2, PTK2, PXN, CDC42, MAPK13, MAPK14, MAPKAPK2, MAPKAPK3, SRC, PIK3CA, PIK3CD, PIK3CB, PIK3R2, PIK3R3, RAC2, AKT1, AKT2, AKT3, CASP9
250μM	hsa04510	Focal adhesion	12.272346	19.96	3.8e-20	COL1A1, COL1A2, COL2A1, COL4A2, COL4A1, COL4A5, COL4A3, COL6A1, COL9A2, LAMA4, LAMC1, RELN, THBS2, THBS3, ITGA2, ITGA3, ITGA5, ITGA6, ITGA7, ITGA11, ITGAV, ITGB1, ITGB6, ITGB8, IGF1, VEGFA, VEGFC, HGF, PDGFRA, PDGFRB, IGF1R, EGFR, FLT1, FLT4, MET, SRC, ARHGAP35, DIAPH1, MYL12B, MYL12A, PPP1CB, PPP1CC, PPP1R12A, MYLK, ACTB, ACTG1, ACTN1, TLN1, TLN2, FLNA, FLNB, PXN, ILK, ZYX, VASP, VCL, PARVB, PARVG, PDPK1, AKT1, AKT2, AKT3, GSK3B, PRKCA, PRKCB, PRKCG, PTK2, PIK3CA, PIK3CD, PIK3CB, PIK3R2, PIK3R3, PTEN, VAV3, VAV2, RAC2, PAK2, PAK3, PAK4, PAK6, CDC42, DOCK1, RAPGEF1, RAP1A, RAP1B, MAPK8, MAPK10, MAPK9, CAV2, CAV3, GRB2, SOS1, SOS2, RAF1, MAPK1, ELK1, CCND1, CCND2, BIRC2, PIP5K1C
250μM	hsa04520	Adherens junction	30.725447	24.54	1.8e-16	PARD3, SRC, CDC42, RAC2, WASL, IQGAP1, WASF1, WASF2, SSX2IP, SORBS1, ACTN1, VCL, TJP1, CDH1, CTNND1, CTNNA2, ACTB, ACTG1, PTPRB, PTPRF, PTPN1, PTPRJ, CSNK2A1, TCF7, TCF7L1, TCF7L2, LEF1, IGF1R, INSR, MET, EGFR, FGFR1, MAPK1, SNAI1, TGFBR1, TGFBR2, SMAD2, SMAD3, CREBBP, EP300, NLK
250μM	hsa04620	Toll-like receptor signaling pathway	16.203301	8.21	8.5e-08	PIK3CA, PIK3CD, PIK3CB, PIK3R2, PIK3R3, AKT1, AKT2, AKT3, TOLLIP, MYD88, IRAK1, TAB2, CHUK, IKBKB, NFKB1, MAPK1, MAP2K3, MAP2K4, MAP2K7, MAPK13, MAPK14, MAPK8, MAPK10, MAPK9, TNF, IRF5, TRAF3, IFNA4, IFNA17, IFNAR2
250μM	hsa04621	NOD-like receptor signaling pathway	10.537661	6.35	2.3e-06	NOD1, CHUK, IKBKB, NFKB1, TNF, TAB2, TAB3, MAPK1, MAPK8, MAPK10, MAPK9, MAPK13, MAPK14, ATG16L1, ATG5, ATG12, GABARAPL1, GABARAPL2, BIRC2, TNFAIP3, TRAF3, IFNA4, IFNA17, NLRX1, ANTXR1, BCL2L1, NLRP3, P2RX7, TRPM7, PLCB1, PLCB2, PLCB4, ITPR1, MCU, OAS2, RNASEL, DHX33, MFN2, DNM1L, NAMPT, TXN, TXN2, NEK7, PRKCD, IFNAR2, TYK2, STAT2, MYD88, TMEM173

250µM	hsa04630	JAK-STAT signaling pathway	18.093825	14.58	2.6e-15	IL15, IFNA4, IFNA17, TSLP, IL2RB, IL2RG, IL6R, IL7R, IL9R, IL12RB1, IL13RA1, IL22RA1, IL22RA2, IL6ST, IFNAR2, IFNLR1, OSMR, CNTFR, CSF2RB, CSF3R, PRLR, EGFR, PDGFRA, PDGFRB, JAK3, TYK2, STAT2, STAT3, STAT5B, CISH, SOCS3, SOCS5, SOCS6, BCL2L1, PIM1, MYC, CCND1, CCND2, CDKN1A, GFAP, STAM2, PTPN2, CREBBP, EP300, PIAS3, FHL1, PTPN11, GRB2, SOS1, SOS2, RAF1, PIK3CA, PIK3CD, PIK3CB, PIK3R2, PIK3R3, AKT1, AKT2, AKT3
250µM	hsa04650	Natural killer cell mediated cytotoxicity	18.954457	8.60	1.5e-05	KLRD1, PTPN11, VAV3, VAV2, RAC2, MAPK1, TNF, PLCG2, SH3BP2, PIK3CA, PIK3CD, PIK3CB, PIK3R2, PIK3R3, GRB2, SOS1, SOS2, KRAS, NRAS, ARAF, RAF1, KLRK1, PPP3CA, PPP3CB, PPP3R1, PPP3R2, NFATC2, PRKCA, PRKCB, PRKCG, SH2D1A, IFNA4, IFNA17, IFNAR2, TNFSF10, FAS, CASP3
250µM	hsa04657	IL-17 signaling pathway	19.904592	10.17	2.1e-10	CASP3, NFKB1, FOSB, JUND, TRAF3, TNFAIP3, TAB2, TAB3, CHUK, IKBKB, MAPK13, MAPK14, MAPK8, MAPK10, MAPK9, MAPK1, CEBPB, TRAF4, USP25, ELAVL1, GSK3B, CXCL5, CCL7, TNF, PTGS2, MMP13
250µM	hsa04658	Th1 and Th2 cell differentiation	17.392751	8.14	1.8e-07	DLL1, MAML2, MAML1, RBPJ, NFKB1, IL12RB1, TYK2, TBX21, RUNX3, HLA-DOB, CD3E, PPP3CA, PPP3CB, PPP3R1, PPP3R2, NFATC2, PRKCQ, CHUK, IKBKB, MAPK1, MAPK13, MAPK14, MAPK8, MAPK10, MAPK9, IL2RB, IL2RG, JAK3, STAT5B, MAF, JAG1, NOTCH1, NOTCH2
250µM	hsa04659	Th17 cell differentiation	17.848781	13.47	2.8e-11	IL1RAP, MAPK13, MAPK14, IRF4, TGFBR1, TGFBR2, SMAD2, SMAD3, IL2RG, JAK3, IL6R, IL6ST, IL12RB1, TYK2, STAT3, RORC, RORA, HIF1A, AHR, HLA-DOB, CD3E, PPP3CA, PPP3CB, PPP3R1, PPP3R2, NFATC2, PRKCQ, CHUK, IKBKB, NFKB1, MAPK1, MAPK8, MAPK10, MAPK9, RUNX1, TBX21, IL2RB, STAT5B, RARA, RXRG
**250µM**	**hsa04660**	**T cell receptor signaling pathway**	**22.545828**	**10.58**	**1.6e-10**	**CD3E, CD8A, PTPRC, ITK, TEC, VAV3, VAV2, GRAP2, GRB2, PAK2, PAK3, PAK4, PAK6, CDC42, DLG1, MAPK13, MAPK14, PPP3CA, PPP3CB, PPP3R1, PPP3R2, NFATC2, SOS1, SOS2, RASGRP1, KRAS, NRAS, RAF1, MAPK1, PRKCQ, MAP2K7, CHUK, IKBKB, NFKB1, CD28, ICOS, PIK3R2, PIK3R3, PIK3CA, PIK3CD, PIK3CB, PDPK1, AKT1, AKT2, AKT3, GSK3B, CBLB, TNF, MAPK9**
250µM	hsa04662	B cell receptor signaling pathway	25.867798	8.72	1.1e-07	VAV3, VAV2, RAC2, PLCG2, PPP3CA, PPP3CB, PPP3R1, PPP3R2, NFATC2, GRB2, SOS1, SOS2, RASGRP3, KRAS, NRAS, RAF1, MAPK1, PRKCB, CHUK, IKBKB, NFKB1, PIK3R2, PIK3R3, PIK3CA, PIK3CD, PIK3CB, AKT1, AKT2, AKT3, GSK3B, CD22
250µM	hsa04668	TNF signaling pathway	18.257914	13.12	1.2e-12	TNF, TNFRSF1A, BAG4, BIRC2, TAB2, TAB3, MAP2K4, MAP2K7, MAPK8, MAPK10, MAPK9, ITCH, MAP2K3, MAPK13, MAPK14, CEBPB, IKBKB, CHUK, NFKB1, MAPK1, RPS6KA5, RPS6KA4, CREB1, CREB3L1, CREB3L2, ATF2, MLKL, DNM1L, CASP7, CASP3, CX3CL1, CSF1, FAS, JAG1, IL15, SOCS3, TNFAIP3, TRAF1, JUNB, MMP14, EDN1, PTGS2, TNFRSF1B, TRAF3, PIK3CA, PIK3CD, PIK3CB, PIK3R2, PIK3R3, AKT1, AKT2, AKT3, DAB2IP, IRF1, CXCL5, VEGFC
250µM	hsa04670	Leukocyte transendothelial migration	19.182176	11.82	2.9e-11	JAM3, JAM2, ITGB1, CD99, CLDN4, CLDN3, CLDN19, CLDN18, CLDN1, ESAM, MSN, ACTB, ACTG1, PIK3CA, PIK3CD, PIK3CB, PIK3R2, PIK3R3, CTNND1, CTNNA2, PTPN11, MAPK13, MAPK14, PLCG2, PRKCA, PRKCB, PRKCG, PTK2, PXN, ARHGAP35, MYL12B, MYL12A, RAP1A, RAP1B, VASP, ACTN1, VCL, GNAI1, GNAI2, RAPGEF3, RAPGEF4, ITK, TXK, VAV3, VAV2, RAC2, CDC42, RHOH
250µM	hsa04721	Synaptic vesicle cycle	13.530971	2.60	3.6e-03	SLC32A1, SLC18A2, SLC17A6, SYT1, VAMP2, RAB3A, STX3, STX1B, STXBP1, CPLX2, CPLX3, CACNA1A, CACNA1B, NAPA, DNM1, DNM3, CLTB, CLTC, AP2B1, AP2M1, ATP6V1A, ATP6V1B2, ATP6V1C1, ATP6V1E1, ATP6V0E2, ATP6V0A1
250µM	hsa04750	Inflammatory mediator regulation of TRP channels	16.069932	8.79	1.0e-08	HTR2A, GNAQ, PLA2G4E, PLCB1, PLCB2, PLCB4, ITPR1, PPP1CB, PPP1CC, ASIC1, ASIC2, ASIC4, CALM2, CALM1, CAMK2A, CAMK2D, CAMK2G, IL1RAP, MAP2K3, MAPK13, MAPK14, MAPK8, MAPK10, MAPK9, PLCG2, PIK3CA, PIK3CD, PIK3CB, PIK3R2, PIK3R3, PRKCD, SRC, PTGER4, ADCY2, ADCY3, PRKACA, PRKACB, PRKACG, TRPV4, PRKCA, PRKCB, PRKCG, PRKCQ, IGF1, TRPV3
250µM	hsa04810	Regulation of actin cytoskeleton	11.497251	20.27	7.8e-19	LPAR1, LPAR4, FGF1, FGF2, FGF17, FGF9, FGF5, FGF18, FGF19, FGF23, EGFR, FGFR1, FGFR2, FGFR3, PDGFRA, PDGFRB, ITGA2, ITGA3, ITGA5, ITGA6, ITGA7, ITGA11, ITGAV, ITGAE, ITGB1, ITGB6, ITGB8, CHRM1, CHRM2, GNG12, FGD1, PTK2, DOCK1, SRC, SOS1, SOS2, KRAS, NRAS, RRAS, RRAS2, MRAS, ARHGEF6, PIK3CA, PIK3CD, PIK3CB, PIK3R2, PIK3R3, VAV3, VAV2, TIAM1, ARAF, RAF1, MAPK1, ARHGEF1, ARHGEF12, ARHGAP35, RAC2, CDC42, PAK2, PAK3, PAK4, PAK6, ARHGEF7, GIT1, MYLK, PPP1CB, PPP1CC, PPP1R12A, MYL12B, MYL12A, DIAPH1, DIAPH2, SLC9A1, PIP5K1C, PIP5K1A, PIP4K2C, PIP4K2A, PIKFYVE, LIMK1, LIMK2, WASL, WASF2, NCKAP1, WASF1, ACTB, ACTG1, PFN1, PXN, RDX, MSN, CFL2, SSH3, SSH2, VCL, IQGAP1, IQGAP3, ACTN1, APC, APC2, ARHGEF4, MYH9, MYH14
250µM	hsa04918	Thyroid hormone synthesis	8.512130	2.58	2.4e-02	CGA, TSHR, ADCY2, ADCY3, PRKACA, PRKACB, PRKACG, GNAQ, PLCB1, PLCB2, PLCB4, PRKCA, PRKCB, PRKCG, PAX8, CREB1, ATF2, CREB3L1, CREB3L2, CANX, ITPR1, SLC5A5, ATP1A2, ATP1B4, ATP1B1, ATP1B2, GPX3, TTR, LRP2
250µM	hsa04919	Thyroid hormone signaling pathway	16.330771	17.27	3.3e-14	PRKACA, PRKACB, PRKACG, ITGAV, KRAS, NRAS, RAF1, MAPK1, ESR1, THRB, NCOR1, HDAC1, HDAC2, THRA, RXRG, KAT2B, NCOA1, NCOA3, CREBBP, EP300, MED12L, MED13, MED14, CCND1, RCAN1, HIF1A, MYC, WNT4, NOTCH1, NOTCH2, PLCB1, PLCB2, PLCB4, PLCG2, PRKCA, PRKCB, PRKCG, SLC9A1, ATP1A2, ATP1B4, ATP1B1, ATP1B2, SLC16A2, SRC, PIK3CA, PIK3CD, PIK3CB, PIK3R2, PIK3R3, PDPK1, AKT1, AKT2, AKT3, GSK3B, TBC1D4, PFKFB2, CASP9, RCAN2, SLC2A1, DIO2, ACTB, ACTG1, ATP2A2, PFKP
250µM	hsa05166	Human T-cell leukemia virus 1 infection	11.139664	14.96	2.3e-12	TGFB2, SLC2A1, NRP1, CD3E, TLN1, TLN2, MYC, CCND2, RANBP3, XPO1, PPP3CA, PPP3CB, PPP3R1, PPP3R2, NFATC2, CANX, IL2RB, IL2RG, JAK3, STAT5B, KRAS, NRAS, RRAS, RRAS2, MRAS, SLC25A5, CDC27, CDC23, CDKN1A, POLD3, CCND1, RB1, E2F1, WNT2, WNT2B, WNT3, WNT3A, WNT4, WNT5B, WNT7B, FZD7, FZD3, FZD4, FZD5, FZD6, DVL3, GSK3B, APC, APC2, DLG1, PDGFRA, PDGFRB, PIK3R2, PIK3R3, PIK3CA, PIK3CD, PIK3CB, AKT1, AKT2, AKT3, TNF, TNFRSF1A, MAP3K1, MAP2K4, MAP3K3, CHUK, IKBKB, NFKB1, LTBR, IL15, BCL2L1, NFYB, HLA-DOB, SRF, ELK4, ELK1, SPI1, ETS1, TBPL1, ADCY2, ADCY3, PRKACA, PRKACB, PRKACG, CREB1, ATF2, ATF3, CRTC1, CRTC2, CRTC3, CREBBP, EP300, KAT2B, POLB, MYBL2, TGFBR1, TGFBR2, SMAD2, SMAD3, CHEK1, TP53INP1, MSX1, MAPK8
250µM	hsa05170	Human immunodeficiency virus 1 infection	8.594465	12.35	4.9e-11	BTRC, FBXW11, GNAI1, GNAI2, GNAO1, PIK3CA, PIK3CD, PIK3CB, PIK3R2, PIK3R3, PTK2, PXN, AKT1, AKT2, AKT3, RPS6KB1, NFKB1, KRAS, NRAS, RAF1, MAPK1, GNAQ, PLCG2, ITPR1, CALM2, CALM1, PPP3CA, PPP3CB, PPP3R1, PPP3R2, NFATC2, PRKCA, PRKCB, PRKCG, GNB2, GNB4, GNG2, GNG4, GNG5, GNG7, GNG12, GNG13, RAC2, PAK2, PAK3, PAK4, PAK6, LIMK1, LIMK2, CFL2, TNF, TNFRSF1B, BCL2L1, MYD88, IRAK1, TAB2, CHUK, IKBKB, IFNA4, IFNA17, MAPK13, MAPK14, MAPK8, MAPK10, MAPK9, TMEM173, APOBEC3H, TRIM5, TNFRSF1A, MAP2K3, MAP2K7, FAS, CASP9, CASP3, CD3E, TAP1, TAP2, AP1G1, AP1S1, CUL5, CUL4A, CHEK1, WEE1, WEE2
250µM	hsa05200	Pathways in cancer	8.225553	30.64	5.3e-26	DCC, CASP3, CASP9, APPL1, CDH1, CTNNA2, AXIN2, APC, APC2, GSK3B, TCF7, TCF7L1, TCF7L2, LEF1, MYC, CCND1, WNT2, WNT2B, WNT3, WNT3A, WNT4, WNT5B, WNT7B, FZD7, FZD3, FZD4, FZD5, FZD6, LRP6, DVL3, FRAT2, LPAR1, LPAR3, LPAR4, LPAR6, ARHGEF12, ARHGEF11, ARHGEF1, GNAI1, GNAI2, PTGER4, ADCY2, ADCY3, PRKACA, PRKACB, PRKACG, GNB2, GNB4, GNG2, GNG4, GNG5, GNG7, GNG12, GNG13, COL4A2, COL4A1, COL4A5, COL4A3, LAMA4, LAMC1, ITGA2, ITGA3, ITGA6, ITGAV, ITGB1, PTK2, PIK3CA, PIK3CD, PIK3CB, PIK3R2, PIK3R3, PTEN, NKX3-1, AKT1, AKT2, AKT3, CHUK, IKBKB, NFKB1, PTGS2, BIRC2, BCL2L1, TRAF1, TRAF3, TRAF4, RPS6KB1, CDKN1B, CDKN1A, BCR, ABL1, CBL, STAT5B, PIM1, PIM2, EDNRA, GNAQ, PLCB1, PLCB2, PLCB4, PRKCA, PRKCB, PRKCG, IL15, IFNA4, IFNA17, IL2RB, IL2RG, CSF2RB, IL6R, IL6ST, IL7R, IL12RB1, IL13RA1, IFNAR2, EML4, RASGRP1, RASGRP3, RASGRP4, JAK3, STAT3, STAT2, VEGFA, VEGFC, TGFA, EGFR, PDGFRA, PDGFRB, IGF1, IGF2, IGF1R, FLT3, HGF, MET, FGF1, FGF2, FGF17, FGF9, FGF5, FGF18, FGF19, FGF23, FGFR1, FGFR2, FGFR3, GRB2, SOS1, SOS2, KRAS, NRAS, ARAF, RAF1, MAPK1, ETS1, RPS6KA5, RET, CCDC6, STK4, DAPK1, PLCG2, ELK1, CALM2, CALM1, CAMK2A, CAMK2D, CAMK2G, RALGDS, RALA, RALB, RALBP1, CDC42, RAC2, MAPK8, MAPK10, MAPK9, PAX8, PPARG, RXRG, RARB, ESR1, NCOA1, NCOA3, SP1, PPARD, JUP, PML, RARA, RUNX1, RUNX1T1, SPI1, CSF3R, CSF1R, E2F1, MAX, CDK6, CCND2, SKP2, CCNE1, CCNE2, RB1, GADD45A, POLK, MITF, TGFB2, TGFBR1, TGFBR2, SMAD2, SMAD3, MECOM, HDAC1, HDAC2, MSH3, FAS, BCL2L11, APAF1, CASP7, NFE2L2, GSTM4, GSTM3, GSTM5, TXNRD1, TXNRD2, CUL2, EGLN1, EGLN3, HIF1A, EPAS1, ARNT2, CREBBP, EP300, SLC2A1, JAG1, DLL1, NOTCH1, NOTCH2, FLT4, SUFU, GLI2, AR
250µM	hsa05205	Proteoglycans in cancer	24.119974	24.12	1.1e-22	CD44, SRC, HCLS1, GRB2, KRAS, NRAS, RRAS, RRAS2, MRAS, RAF1, ARAF, MAPK1, IQGAP1, CDC42, ELK1, ESR1, CCND1, ACTB, ACTG1, FLNA, FLNB, TIAM1, ARHGEF1, ANK2, ANK3, GAB1, PIK3CA, PIK3CD, PIK3CB, PIK3R2, PIK3R3, AKT1, AKT2, AKT3, SLC9A1, PPP1CB, PPP1CC, PPP1R12A, ARHGEF12, ITPR1, CAMK2A, CAMK2D, CAMK2G, STAT3, TWIST2, IGF1, IGF1R, PDPK1, RPS6KB1, EIF4B, EGFR, CAV2, CAV3, CDKN1A, CASP3, TNF, ERBB3, MYC, HIF1A, TFAP4, VEGFA, MET, CBL, TIMP3, FAS, TGFB2, SDC1, ITGA2, ITGB1, ITGAV, HGF, FZD7, FZD3, FZD4, FZD5, FZD6, ITGA5, SDC2, RDX, MSN, FGF2, FGFR1, FRS2, PTPN11, SOS1, SOS2, PLCG2, PRKCA, PRKCB, PRKCG, PXN, PTK2, HBEGF, IGF2, WNT2, WNT2B, WNT3, WNT3A, WNT4, WNT5B, WNT7B, IHH, HSPG2, PRKACA, PRKACB, PRKACG, MAPK13, MAPK14, SMAD2, VAV2

250μM	hsa05206	MicroRNAs in cancer	19.751687	12.74	3.3e-13	PTEN, CDKN1B, TIMP3, KRAS, CDK6, CDC25A, CDC25B, HMGA2, CCND1, CCND2, CCNE1, CCNE2, PIM1, MET, DNMT3A, DNMT3B, BCL2L2, EGFR, VEGFA, TGFB2, CREBBP, EP300, SPRY2, ZEB1, ZEB2, ST14, ERBB3, ABL1, TP63, DDIT4, BMF, PAK4, SLC7A1, CCNG1, STMN1, STAT3, MYC, CASP3, NRAS, DNMT1, APC, APC2, IRS1, HNRNPK, RECK, IRS2, NOTCH1, NOTCH2, BMI1, CD44, DICER1, FZD3, ITGA5, RDX, ZFPM2, UBE2I, CDKN1A, NFKB1, IKBKB, PTGS2, PRKCA, PRKCB, PRKCG, GRB2, FGFR3, RPS6KA5, MDM4, PDGFRA, PLCG2, RAF1, SOS1, SOS2, PDGFRB, E2F1, BCL2L11, BMPR2, GLS, SIRT1, WNT3, WNT3A, KIF23, EFNA3, FOXP1, HDAC1, PIK3CA, PIK3R2, MAPK1, FSCN1, HDAC4
