# Supplementary material for: The DCMU Herbicide Shapes T-cell Functions By Modulating Micro-RNA Expression Profiles
Source: Front Immunol. 2022 Jul 28;13:925241. doi: 10.3389/fimmu.2022.925241 (PMC9366666; doi:10.3389/fimmu.2022.925241)
Supplement: Supplementary file 3 [file DataSheet_3.pdf]

Supplemental Table 3. mRNA dysregulated in CTL03.1 cells in response to DCMU exposure during 24h.

DCMU dose (μM)	Gene	BaseMean	log2 Fold Change	SE (log2 Fold Change)	pvalue
10	IER5	156.3	0.871	0.169	2.55e-07
10	TPT1	3910.	0.428	0.092	3.45e-06
10	HLA-A	1659.	0.490	0.116	0.000025
10	HLA-F	180.0	0.576	0.178	0.001216
10	FERMT3	283.1	0.452	0.147	0.002107
10	CD58	223.6	0.499	0.164	0.002418
10	AEN	77.78	0.660	0.222	0.003002
10	RPS12	6263.	0.244	0.084	0.003986
10	HLA-B	1059.	0.300	0.104	0.004033
10	PLEKHO1	228.2	0.440	0.154	0.004299
10	NMI	102.7	0.650	0.229	0.004577
10	RAC1	135.1	0.498	0.175	0.004581
10	TAPBP	245.4	0.408	0.144	0.004828
10	COMT	153.8	0.492	0.183	0.007199
10	RFC1	130.4	0.590	0.223	0.008283
10	MAEA	178.9	0.447	0.171	0.009201
10	AOAH	620.0	0.637	0.246	0.009674
10	RPL14	1960.	0.238	0.092	0.009787
10	RBFOX2	4.090	-2.80	1.095	0.010368
10	MGAT4B	41.21	0.805	0.319	0.011583
10	RPS11	2692.	0.220	0.087	0.011748
10	MMP24OS	5.261	2.320	0.926	0.012231
10	RAC2	705.6	0.271	0.108	0.012255
10	RPL37	2158.	0.258	0.103	0.012477
10	RPL17	1783.	0.211	0.085	0.013702
10	ZNHIT1	167.9	0.404	0.165	0.014579
10	MMP25	125.2	0.442	0.181	0.014834
10	ABHD11	2.947	3.324	1.384	0.016316
10	CORO1A	197.0	0.377	0.157	0.016801
10	ATP2B4	136.4	0.409	0.174	0.018826
10	GNA15	2.781	3.022	1.336	0.023754
10	CD37	487.3	0.258	0.116	0.025938
10	OFD1	57.01	0.608	0.273	0.026284
10	HMGB2	983.8	0.589	0.266	0.026754
10	RPL24	2483.	0.220	0.099	0.026760
10	ABTB2	1.916	3.561	1.616	0.027516
10	RPL38	1623.	0.207	0.094	0.027847
10	ANGEL1	3.455	2.494	1.137	0.028339
10	ASS1	29.75	-0.87	0.401	0.029655
10	MYH7B	119.9	0.419	0.193	0.029987
10	PCNP	91.96	-0.45	0.209	0.030317
10	RPLP2	3663.	0.202	0.094	0.030844
10	UBA52	2800.	0.178	0.082	0.031103
10	MYPOP	32.81	0.723	0.336	0.031533
10	PTPRE	42.67	0.681	0.321	0.033788
10	ITGA1	97.23	0.474	0.224	0.034292
10	ODAPH	89.68	0.509	0.242	0.035598
10	MFSD14B	12.07	-1.24	0.593	0.036077
10	EEF1A1	9942.	0.186	0.088	0.036128
10	TOMM7	899.8	0.197	0.095	0.037358
10	CCND3	107.3	0.404	0.197	0.040111
10	PLAAT4	602.7	0.219	0.107	0.040160
10	TAF1D	77.27	0.563	0.276	0.041259
10	ACTN4	48.85	0.595	0.292	0.041459
10	PARD6B	2.474	-2.85	1.402	0.042053
10	MFAP5	1.649	-3.42	1.689	0.042706
10	FHOD1	60.71	0.524	0.258	0.042746
10	TSPAN4	6.218	-1.58	0.784	0.043122
10	RPL27A	2335.	0.188	0.093	0.043356
10	ANKRD36B	44.74	0.670	0.332	0.043526
10	RPS24	1965.	0.200	0.099	0.044303
10	RPL23	2634.	0.180	0.090	0.045643
10	GNAI2	67.13	0.483	0.242	0.045657
10	LMAN1	107.2	0.436	0.219	0.046808
10	ZCCHC8	28.01	0.751	0.378	0.046818
10	CYP1B1	82.13	-0.51	0.257	0.047483
10	JUND	25.07	0.774	0.390	0.047538
10	RPS21	1632.	0.204	0.103	0.048065
10	CYTIP	261.2	0.282	0.143	0.048810

100	CYP1B1	55.03	-1.48	0.285	1.84e-07
100	IL32	4723.	-0.45	0.096	3.26e-06
100	MYH7B	157.9	0.906	0.203	8.61e-06
100	NPC2	282.3	0.637	0.148	0.000016
100	XCL1	81.88	-1.05	0.246	0.000018
100	ZRANB2	194.5	0.696	0.166	0.000028
100	UCP2	191.8	-0.64	0.158	0.000050
100	TXNIP	974.5	-1.09	0.270	0.000054
100	PFN1	802.5	-0.41	0.106	0.000094
100	FTL	14599	0.439	0.117	0.000171
100	CCL5	11098	0.409	0.109	0.000178
100	S100A4	411.6	-0.46	0.124	0.000185
100	UPK3BL2	71.07	-0.94	0.252	0.000197
100	SOX4	56.54	1.085	0.293	0.000211
100	CTSW	310.9	-0.47	0.135	0.000381
100	CORO1A	190.7	-0.60	0.170	0.000390
100	FERMT3	276.8	-0.59	0.167	0.000418
100	XCL2	49.13	-1.11	0.315	0.000425
100	GLO1	278.7	-0.49	0.141	0.000434
100	RBM3	215.9	-0.51	0.151	0.000648
100	AGTRAP	305.1	-0.45	0.135	0.000744
100	PLAAT4	532.4	-0.40	0.120	0.000824
100	LPXN	312.1	-0.44	0.133	0.000882
100	NEAT1	74.19	0.802	0.244	0.001043
100	IL7R	48.29	0.915	0.286	0.001383
100	ACAA2	317.0	-0.41	0.130	0.001402
100	GAS5	508.0	0.386	0.121	0.001471
100	LPAR6	175.4	-0.50	0.161	0.001767
100	LINC00892	150.0	-0.54	0.173	0.001804
100	GNLY	1015.	0.361	0.117	0.002084
100	ACTB	1409.	-0.36	0.119	0.002202
100	TMEM258	920.2	0.355	0.116	0.002367
100	ZNF207	136.8	-0.53	0.180	0.002981
100	HOPX	679.4	-0.33	0.115	0.003226
100	NCR3	217.9	-0.44	0.151	0.003443
100	CD40LG	211.0	-0.45	0.155	0.003671
100	DHCR24	18.24	-1.41	0.491	0.003946
100	PTMA	3607.	0.321	0.113	0.004477
100	CCNG1	149.7	0.477	0.168	0.004615
100	ACTG1	1263.	-0.27	0.099	0.005044
100	GPX4	151.7	0.500	0.181	0.005888
100	DDIT3	88.28	0.637	0.231	0.005939
100	MALAT1	7465.	0.291	0.106	0.005989
100	ID2	912.7	-0.33	0.123	0.006274
100	LMAN1	114.1	0.570	0.209	0.006458
100	RCBTB2	350.3	-0.41	0.153	0.006760
100	SLAMF7	197.0	-0.42	0.156	0.006802
100	SRSF3	248.7	-0.38	0.144	0.007215
100	EWSR1	41.96	-0.77	0.292	0.008009
100	TNFAIP3	13.71	1.368	0.518	0.008297
100	RAC2	617.2	-0.30	0.115	0.008361
100	TP53I13	73.47	-0.66	0.250	0.008401
100	RFXANK	37.52	-0.79	0.304	0.008993
100	SNRPB2	420.0	-0.32	0.126	0.009704
100	LOC150051	39.25	0.797	0.310	0.010139
100	FDFT1	54.35	-0.68	0.265	0.010386
100	PSTPIP1	138.2	-0.47	0.184	0.010489
100	CAMK1	42.99	-0.76	0.298	0.010778
100	CDC14A	16.55	1.287	0.507	0.011222
100	MSMO1	103.6	-0.50	0.201	0.011865
100	LOC283788	27.87	0.896	0.360	0.012731
100	ACIN1	35.97	0.795	0.324	0.014357
100	PRKCE	2.841	3.241	1.339	0.015506
100	CFL1	246.5	-0.38	0.159	0.016219
100	JPT1	130.7	-0.43	0.180	0.016498
100	SYNE2	84.26	0.513	0.215	0.016987
100	GRK2	213.7	-0.44	0.188	0.017469
100	BDP1	51.71	0.661	0.279	0.017733
100	DPAGT1	37.53	0.726	0.306	0.017772
100	HMGCR	18.52	-1.05	0.444	0.017840
100	CD48	604.1	-0.32	0.136	0.018213
100	UBE2N	202.1	-0.37	0.159	0.018246
100	CRIM1	16.98	-1.14	0.487	0.018606
100	ARRDC3	95.00	0.529	0.225	0.018901

100	SH3BGRL3	4588.	-0.23	0.101	0.018968
100	ERO1A	47.55	-0.68	0.292	0.019181
100	LDLR	10.16	-1.40	0.599	0.019353
100	APOC1	71.26	0.867	0.371	0.019630
100	GBP2	124.6	0.445	0.190	0.019672
100	LTB	381.4	-0.36	0.158	0.019983
100	TBCB	55.95	-0.61	0.266	0.020720
100	AIF1	183.3	-0.37	0.160	0.020806
100	PPIB	628.4	0.289	0.125	0.020991
100	TTC28-AS1	23.38	0.874	0.380	0.021527
100	MAEA	171.0	-0.44	0.193	0.022231
100	PSAP	450.3	0.291	0.127	0.022418
100	CSDE1	241.1	0.340	0.149	0.022858
100	TUBA4A	159.5	-0.39	0.172	0.022933
100	VPS13A	16.31	1.045	0.459	0.023033
100	LIMD2	62.86	-0.58	0.256	0.023542
100	LRRN3	411.4	-0.29	0.131	0.023876
100	THEMIS	182.7	-0.36	0.163	0.023953
100	PGK1	523.8	-0.25	0.114	0.025483
100	TNF	36.84	-0.73	0.328	0.025555
100	CD58	205.5	-0.40	0.182	0.026135
100	NAP1L1	354.4	0.312	0.140	0.026494
100	PDE7B	17.94	0.961	0.433	0.026663
100	ITGAL	168.8	-0.37	0.168	0.026743
100	SLC35B4	7.121	-1.58	0.714	0.026963
100	LINC01260	13.99	-1.09	0.497	0.027743
100	CTSC	973.6	-0.22	0.103	0.027762
100	CALM1	733.0	-0.24	0.111	0.028479
100	PPIA	1859.	-0.21	0.099	0.029337
100	ZNF581	6.962	1.597	0.734	0.029590
100	SELENOK	461.0	0.283	0.130	0.029989
100	BTN3A2	97.73	0.446	0.206	0.030095
100	MOSPD3	10.80	-1.23	0.573	0.030694
100	FRMD8	6.232	-1.62	0.754	0.031423
100	ADA	82.71	-0.50	0.235	0.031621
100	PARK7	459.4	-0.26	0.125	0.031754
100	GOLGB1	64.64	0.524	0.244	0.031889
100	AKR1B1	82.56	0.500	0.233	0.032221
100	YDJC	11.84	-1.16	0.548	0.033037
100	LYSMD3	15.82	0.969	0.457	0.034195
100	ARFGAP3	87.54	0.476	0.225	0.034376
100	BTAF1	14.39	1.021	0.485	0.035375
100	RBX1	600.7	-0.24	0.114	0.036109
100	AHI1	83.76	0.445	0.212	0.036301
100	LRPAP1	71.47	-0.56	0.269	0.036590
100	MCRS1	69.90	-0.48	0.233	0.037073
100	PNISR	205.2	0.325	0.156	0.037230
100	RSRC2	106.9	-0.39	0.192	0.038494
100	C12orf57	307.8	0.297	0.144	0.039267
100	STOM	111.2	-0.39	0.194	0.040328
100	C2orf49	21.26	0.814	0.397	0.040463
100	TMEM167B	56.02	0.515	0.252	0.041097
100	KLF10	9.193	-1.29	0.633	0.041215
100	CAPN1	13.08	-1.03	0.506	0.041266
100	KNOP1	29.93	0.706	0.348	0.042607
100	NAT14	1.243	3.723	1.836	0.042651
100	ITGB2	78.75	-0.48	0.239	0.042735
100	CLIC4	5.847	1.578	0.779	0.042918
100	CD53	859.5	-0.22	0.110	0.043526
100	UNC119B	3.825	2.064	1.025	0.044073
100	DIPK1A	15.83	1.001	0.498	0.044368
100	PYCR1	1.948	2.907	1.449	0.044867
100	DENND11	5.573	1.577	0.786	0.044894
100	FKBP1A	231.2	-0.29	0.148	0.045049
100	RBBP9	7.129	1.512	0.757	0.045859
100	HMGN5	3.072	-2.33	1.173	0.046186
100	GUSBP14	1.663	3.235	1.624	0.046357
100	IFITM2	889.9	-0.21	0.107	0.046745
100	OR4D2	28.51	0.697	0.351	0.046934
100	TESC	40.99	-0.59	0.299	0.047294
100	RNF167	109.4	-0.38	0.195	0.047399
100	PLPP5	39.08	0.621	0.313	0.047535
100	ANXA6	204.7	-0.30	0.153	0.047557
100	CXCR4	122.8	0.381	0.192	0.047855

100	FHOD1	66.51	-0.50	0.257	0.047901
100	HLA-F-AS1	7.751	1.304	0.659	0.047931
100	RHOA	736.1	-0.22	0.111	0.048319
100	LINC02482	14.72	0.955	0.484	0.048371
100	ACSL6	12.76	-1.08	0.552	0.048520
100	RAD51AP1	6.155	1.522	0.771	0.048614
100	PRKD2	66.11	-0.50	0.253	0.048640
100	GUSBP11	1.483	3.307	1.678	0.048770
100	MDN1	8.072	1.281	0.650	0.048884
100	WDR54	73.17	-0.44	0.225	0.049201
100	LCLAT1	3.688	-2.09	1.065	0.049318
100	RPS27L	2136.	-0.18	0.093	0.049547
100	VPS35	83.93	0.449	0.229	0.049867
250	DDIT3	134.3	2.271	0.225	6.16e-24
250	MYH7B	212.2	2.065	0.240	8.76e-18
250	TXNIP	482.1	-2.19	0.295	1.11e-13
250	LOC150051	41.34	2.155	0.290	1.14e-13
250	RGS1	37.21	2.303	0.325	1.38e-12
250	SLC3A2	37.69	1.921	0.303	2.28e-10
250	ATF4	125.8	1.212	0.198	9.20e-10
250	NPC2	228.7	1.224	0.203	1.87e-09
250	CXCR4	120.0	1.688	0.284	3.07e-09
250	IL32	2455.	-1.32	0.225	3.96e-09
250	NCR3	90.11	-1.37	0.235	5.15e-09
250	LURAP1L	19.65	2.495	0.435	9.71e-09
250	RN7SK	25.96	2.029	0.360	1.75e-08
250	RCBTB2	165.2	-1.11	0.200	2.88e-08
250	HOPX	345.2	-1.14	0.211	6.66e-08
250	FTL	11555	0.850	0.161	1.43e-07
250	PFN1	413.0	-0.95	0.182	1.55e-07
250	NEAT1	79.14	1.538	0.302	3.54e-07
250	PLPP5	36.55	1.496	0.294	3.67e-07
250	SETD1A	20.82	1.958	0.398	8.78e-07
250	LINC00892	59.97	-1.45	0.297	9.85e-07
250	TMEM258	702.4	0.747	0.153	1.17e-06
250	CXCR3	88.86	-1.10	0.234	2.37e-06
250	ARRDC3	92.53	1.056	0.227	3.52e-06
250	AGTRAP	146.0	-1.05	0.231	4.77e-06
250	UPK3BL2	37.27	-1.68	0.370	5.34e-06
250	LPAR6	89.64	-1.22	0.270	6.21e-06
250	ANXA6	107.9	-1.02	0.226	6.52e-06
250	ACTB	768.6	-0.85	0.189	6.77e-06
250	DNAJB9	35.56	1.298	0.290	7.91e-06
250	LTB	162.4	-1.21	0.272	8.59e-06
250	XCL1	25.87	-2.05	0.466	0.000010
250	CYP1B1	42.43	-1.56	0.355	0.000010
250	MSMO1	55.20	-1.22	0.278	0.000011
250	SERP1	56.16	1.054	0.241	0.000012
250	XCL2	16.11	-1.99	0.458	0.000013
250	SOX4	79.02	1.402	0.324	0.000015
250	RBM3	102.3	-0.94	0.219	0.000016
250	HM13	36.43	1.211	0.289	0.000029
250	CCR2	63.04	-1.12	0.269	0.000029
250	GMFG	545.5	-0.74	0.179	0.000032
250	UCP2	94.04	-1.15	0.278	0.000034
250	BTG1	73.82	0.939	0.227	0.000035
250	CD52	4583.	-0.68	0.167	0.000038
250	SRSF3	141.7	-0.82	0.202	0.000041
250	BRI3	11.68	2.394	0.584	0.000042
250	LUC7L3	172.1	0.759	0.185	0.000042
250	AHI1	63.94	1.197	0.298	0.000060
250	KDELR2	24.87	1.329	0.335	0.000073
250	CAMK1	20.64	-1.77	0.447	0.000074
250	CTSW	145.6	-0.87	0.220	0.000076
250	IL7R	35.20	1.204	0.305	0.000081
250	GPX4	117.7	0.800	0.204	0.000092
250	ITGB2	36.41	-1.20	0.307	0.000096
250	OSTC	259.2	0.715	0.184	0.000104
250	SH3BGRL3	2554.	-0.59	0.154	0.000106
250	ANKRD36C	46.76	1.058	0.274	0.000115
250	GAS5	361.7	0.739	0.195	0.000148
250	CLIC1	594.5	-0.63	0.167	0.000148
250	MANF	27.66	1.277	0.337	0.000154
250	CD40LG	93.75	-1.00	0.265	0.000160

250	DPEP2	22.76	1.374	0.364	0.000161
250	ARFGAP3	67.01	0.902	0.239	0.000163
250	CISH	51.89	-1.10	0.296	0.000189
250	CCL5	7820.	0.722	0.194	0.000208
250	NUCB2	104.5	0.824	0.223	0.000222
250	CCL3	60.42	1.160	0.314	0.000229
250	GNPDA1	20.66	1.331	0.361	0.000230
250	IFITM1	1094.	-0.59	0.162	0.000236
250	LRRN3	195.9	-0.81	0.222	0.000248
250	CCNG1	115.4	0.793	0.217	0.000263
250	SELENOK	333.1	0.598	0.164	0.000266
250	MYL12A	1692.	-0.58	0.160	0.000283
250	GNL3	44.68	0.969	0.267	0.000290
250	DUSP6	26.30	-1.52	0.421	0.000295
250	LZTFL1	24.74	1.236	0.342	0.000303
250	CORO1A	87.51	-0.84	0.236	0.000344
250	JPT1	73.49	-0.85	0.241	0.000381
250	SIRPG	22.06	-1.50	0.424	0.000384
250	TUBA4A	79.74	-1.08	0.305	0.000386
250	PSME2	322.9	-0.57	0.162	0.000411
250	CHI3L1	39.38	1.620	0.458	0.000411
250	ANKRD36B	37.32	1.103	0.313	0.000428
250	RAC2	317.5	-0.77	0.220	0.000434
250	GM2A	60.36	1.131	0.324	0.000490
250	SLAMF7	94.31	-0.91	0.263	0.000507
250	RPL28	2291.	0.653	0.188	0.000512
250	KLF10	5.923	-2.79	0.803	0.000513
250	ITGAL	91.03	-0.87	0.252	0.000516
250	XAF1	24.04	-1.30	0.375	0.000534
250	RGS2	30.65	1.132	0.327	0.000534
250	TNFAIP3	11.53	1.721	0.500	0.000581
250	MTHFD2	78.23	0.869	0.253	0.000619
250	S100A4	204.9	-0.79	0.231	0.000620
250	SLC8A1-AS1	13.51	-1.95	0.581	0.000757
250	STT3A	77.48	0.846	0.252	0.000797
250	TMEM14B	133.7	-0.65	0.195	0.000804
250	AIF1	73.57	-0.85	0.253	0.000816
250	SSR1	159.1	0.630	0.188	0.000829
250	SDCBP	110.4	0.701	0.211	0.000883
250	SOS1	38.01	-1.14	0.346	0.000891
250	IFITM2	452.9	-0.55	0.167	0.000927
250	LYZ	42.88	1.344	0.407	0.000968
250	ID2	434.0	-0.90	0.274	0.000985
250	ERN1	20.49	1.282	0.389	0.000997
250	TNFAIP8L2	41.32	-0.98	0.299	0.001048
250	ISG20	25.22	1.213	0.370	0.001067
250	HSPA8	914.0	-0.49	0.152	0.001085
250	PLAAT4	272.5	-0.73	0.226	0.001090
250	MYL6	2155.	-0.46	0.142	0.001142
250	ATP5F1E	468.1	-0.58	0.179	0.001198
250	HSPA13	31.66	1.073	0.331	0.001208
250	C7orf50	20.90	1.205	0.374	0.001298
250	CD70	50.38	-0.84	0.265	0.001446
250	NANS	11.76	1.511	0.477	0.001538
250	LGALS1	1308.	-0.45	0.142	0.001558
250	FIS1	173.4	-0.74	0.237	0.001664
250	TMEM167B	41.78	0.853	0.273	0.001803
250	GLO1	139.6	-0.66	0.213	0.001830
250	RNASE6	22.79	-1.21	0.390	0.001833
250	SNRPG	300.9	-0.54	0.174	0.001893
250	LCK	110.1	-0.68	0.221	0.002008
250	CANX	62.60	0.808	0.261	0.002018
250	SELENOS	50.55	0.806	0.261	0.002047
250	ARL6IP5	385.0	-0.53	0.174	0.002106
250	CCND3	49.63	-0.85	0.277	0.002189
250	SPCS2	237.1	0.548	0.179	0.002230
250	RPN2	283.8	0.607	0.201	0.002614
250	BNIP3L	68.96	0.698	0.232	0.002694
250	MRPL54	51.74	-0.82	0.273	0.002727
250	PTMA	2559.	0.485	0.162	0.002783
250	SLFN11	10.83	1.472	0.492	0.002803
250	MCRS1	40.48	-0.85	0.286	0.002870
250	A2M	11.66	2.408	0.808	0.002876
250	RHOC	208.8	-0.57	0.191	0.002879

250	DNAJC15	69.94	-0.72	0.245	0.002954
250	MTERF3	7.601	1.704	0.576	0.003114
250	ACAA2	177.1	-0.56	0.192	0.003140
250	ITGAE	50.33	0.762	0.258	0.003142
250	IFNG	19.91	-1.37	0.465	0.003163
250	RABGAP1L	47.59	0.788	0.267	0.003169
250	ZSWIM8	6.481	1.889	0.640	0.003179
250	PYCARD	26.95	-1.03	0.351	0.003302
250	NDUFA4	594.0	-0.45	0.155	0.003303
250	DCXR	54.85	0.722	0.246	0.003317
250	TTN	36.72	0.986	0.336	0.003345
250	BTG2	22.38	-1.11	0.383	0.003597
250	TMEM147	33.65	0.843	0.290	0.003663
250	UBL5	414.6	-0.44	0.154	0.003886
250	TIPARP	9.359	-1.63	0.566	0.003919
250	EPB41	11.09	1.455	0.504	0.003931
250	ARHGDIB	1090.	-0.51	0.180	0.003977
250	FCRL6	7.592	-1.87	0.651	0.004006
250	TTC3	45.73	0.828	0.288	0.004033
250	FTH1	241.0	0.482	0.167	0.004070
250	KLHL6	12.92	1.311	0.456	0.004110
250	ARF4	121.0	0.554	0.193	0.004148
250	TESC	21.08	-1.09	0.383	0.004183
250	PARK7	268.3	-0.52	0.183	0.004399
250	BTN3A1	40.96	0.771	0.271	0.004416
250	HCST	576.1	-0.60	0.211	0.004428
250	SSR3	194.5	0.590	0.208	0.004525
250	IGLL5	52.68	1.086	0.383	0.004579
250	RAB37	11.36	-1.60	0.568	0.004662
250	.OC10028817!	6.162	1.851	0.657	0.004855
250	HSP90B1	276.2	0.515	0.183	0.004887
250	GPR15	117.3	-0.86	0.306	0.005001
250	COX8A	473.2	-0.44	0.159	0.005037
250	SPSB3	16.67	1.096	0.391	0.005072
250	SQSTM1	57.84	0.931	0.333	0.005219
250	APOC1	55.06	1.235	0.442	0.005270
250	CD48	298.7	-0.62	0.224	0.005321
250	CALM1	418.4	-0.60	0.218	0.005357
250	ZRANB2	139.6	0.710	0.256	0.005649
250	CLEC2D	134.4	0.560	0.203	0.005970
250	AK1	12.64	1.357	0.498	0.006482
250	MMP12	26.93	1.493	0.548	0.006484
250	ENO1	915.7	-0.39	0.146	0.006511
250	ATP5MF	328.2	-0.47	0.174	0.006601
250	TXNDC17	106.7	-0.61	0.225	0.006614
250	PABPC4	10.12	1.481	0.545	0.006640
250	RPA1	17.32	-1.10	0.408	0.006689
250	ANXA1	569.4	-0.42	0.156	0.006694
250	CSGALNACT2	15.38	1.154	0.425	0.006743
250	HSPA5	92.78	0.644	0.238	0.006918
250	OAS3	7.551	-2.07	0.770	0.007042
250	CCR5	35.58	-0.88	0.326	0.007061
250	CCND2	100.6	-0.61	0.227	0.007099
250	UFM1	86.07	0.590	0.219	0.007185
250	NDUFB3	205.5	-0.48	0.179	0.007269
250	ACTG1	694.0	-0.42	0.157	0.007272
250	ERG28	71.30	-0.65	0.243	0.007364
250	FN1	6.548	1.780	0.664	0.007418
250	GIHCG	20.94	1.050	0.392	0.007427
250	GALT	22.27	-0.98	0.366	0.007469
250	NKG7	2035.	-0.51	0.191	0.007554
250	ARPC2	288.9	-0.47	0.178	0.007568
250	ZNF202	1.949	3.509	1.314	0.007602
250	SEC11C	140.4	0.726	0.272	0.007633
250	BTN3A2	69.68	0.613	0.230	0.007752
250	GK	9.414	1.417	0.532	0.007758
250	POLR2K	135.5	-0.53	0.200	0.007782
250	BPTF	30.91	0.922	0.347	0.007901
250	CDC6	5.242	1.951	0.735	0.007973
250	CD53	458.0	-0.55	0.211	0.007995
250	HEATR9	3.680	2.702	1.019	0.008002
250	IDI1	105.0	-0.58	0.222	0.008058
250	MPHOSPH8	125.4	0.560	0.212	0.008344
250	KCTD7	2.834	-2.86	1.088	0.008446

250	ORMDL2	101.5	-0.59	0.225	0.008528
250	SFXN1	27.94	-1.07	0.407	0.008544
250	THEMIS	96.77	-0.60	0.228	0.008552
250	DDX55	12.12	1.267	0.482	0.008555
250	STAT5B	7.895	-1.63	0.622	0.008577
250	SPN	30.38	-0.89	0.342	0.008790
250	SNHG8	76.32	0.579	0.222	0.009076
250	TGIF1	8.962	1.370	0.526	0.009171
250	CKS1B	64.81	0.675	0.259	0.009229
250	SRA1	41.59	-0.73	0.283	0.009265
250	NINJ2	2.844	-3.21	1.237	0.009360
250	DYNLL1	444.2	-0.40	0.154	0.009472
250	TIMM8B	119.1	-0.55	0.212	0.009477
250	IL4	16.11	-1.17	0.454	0.009568
250	MS4A1	5.349	2.643	1.021	0.009673
250	CFDP1	53.37	0.640	0.247	0.009691
250	RBX1	318.9	-0.42	0.165	0.009814
250	AARS1	18.79	0.960	0.372	0.009894
250	PDE7B	13.89	1.106	0.429	0.009918
250	ATP1B1	13.28	-1.26	0.490	0.009962
250	FAM3C	29.38	0.939	0.364	0.010004
250	MX1	34.65	-0.88	0.345	0.010069
250	SUB1	352.8	-0.44	0.174	0.010181
250	USB1	12.88	-1.22	0.476	0.010191
250	KRTCAP2	313.2	0.447	0.174	0.010280
250	PSMB3	237.1	-0.43	0.169	0.010301
250	EML4	46.66	0.669	0.261	0.010347
250	ESYT1	26.29	-0.90	0.353	0.010433
250	RNF113A	65.84	0.601	0.234	0.010452
250	SEC61G	396.5	0.449	0.175	0.010674
250	MED11	27.10	-0.94	0.368	0.010676
250	ABCG1	2.717	3.206	1.256	0.010728
250	PLEK	12.30	1.693	0.664	0.010766
250	PLIN2	93.78	0.640	0.251	0.010945
250	RTN3	27.65	1.052	0.413	0.011010
250	RPS27L	1219.	-0.44	0.174	0.011281
250	MAB21L3	5.518	1.759	0.694	0.011281
250	HINT2	28.56	-0.83	0.329	0.011400
250	SSR4	787.2	0.421	0.166	0.011472
250	PIM1	39.28	-0.76	0.304	0.011636
250	RTF2	176.3	-0.58	0.230	0.011675
250	NDUFB1	199.6	-0.49	0.195	0.011815
250	SYTL2	14.26	1.150	0.457	0.011922
250	SLC25A6	152.0	0.488	0.194	0.012048
250	CFL1	130.8	-0.49	0.197	0.012165
250	CDK2AP2	71.55	0.664	0.265	0.012298
250	SARAF	182.4	0.439	0.175	0.012366
250	SRP14	731.7	-0.37	0.151	0.012380
250	PRDX6	296.0	0.444	0.178	0.012728
250	NEDD9	28.43	-1.03	0.416	0.012753
250	SNRPB2	237.4	-0.44	0.180	0.012754
250	C1orf174	19.20	1.006	0.404	0.012771
250	RUNX1	5.961	-1.81	0.731	0.013157
250	CCR7	27.08	0.973	0.393	0.013284
250	CSF1	4.483	-2.49	1.010	0.013413
250	PGM3	14.17	1.035	0.418	0.013445
250	CCR6	9.975	-1.41	0.573	0.013586
250	GLIPR2	36.15	-0.78	0.319	0.013657
250	GNGT2	13.18	-1.18	0.481	0.013674
250	TMEM43	32.60	0.820	0.332	0.013729
250	FBXO6	27.30	-0.85	0.345	0.013752
250	TNF	18.14	-0.97	0.395	0.013870
250	PTPN7	20.75	-1.05	0.428	0.013951
250	MYL12B	756.6	-0.40	0.163	0.013998
250	HSP90AA1	264.1	-0.43	0.176	0.014017
250	RASAL3	32.42	0.815	0.333	0.014400
250	C3orf18	9.772	-1.42	0.584	0.014554
250	LY96	19.62	0.940	0.385	0.014728
250	SP110	74.81	-0.74	0.304	0.014890
250	ITPR2	25.03	0.968	0.397	0.014911
250	NAA20	56.41	-0.61	0.253	0.015133
250	APOBEC3H	184.7	-0.46	0.190	0.015136
250	TNFSF10	78.56	-0.60	0.249	0.015176
250	TMED2	56.14	0.623	0.256	0.015242

250	EID1	46.25	-0.77	0.318	0.015245
250	CD3D	1307.	-0.39	0.161	0.015278
250	ANKRD36	26.84	0.895	0.369	0.015366
250	UBE2N	123.6	-0.49	0.203	0.015466
250	COMMD1	34.87	-0.92	0.384	0.015614
250	ANKRD44-AS1	22.18	-0.90	0.374	0.015623
250	IFI27	480.4	-0.52	0.218	0.015851
250	ZNF207	72.19	-0.63	0.261	0.015919
250	PARP9	29.76	-0.77	0.323	0.016294
250	MIB2	3.317	2.102	0.875	0.016375
250	BISPR	11.44	-1.20	0.501	0.016376
250	ATP5PD	309.6	-0.39	0.164	0.016398
250	CD44	55.27	0.637	0.265	0.016437
250	ODC1	11.41	1.311	0.547	0.016495
250	ZNF574	2.668	2.445	1.020	0.016611
250	SASH3	18.65	-0.96	0.404	0.016639
250	BET1	51.05	0.646	0.270	0.016766
250	RNF44	6.241	-1.57	0.659	0.016862
250	APOBEC3C	246.8	-0.41	0.172	0.016919
250	PPHLN1	37.41	-0.72	0.302	0.017164
250	PAQR7	2.193	2.831	1.192	0.017595
250	LINC01873	17.04	0.919	0.387	0.017743
250	LST1	31.42	-0.76	0.322	0.018139
250	TMEM140	20.82	1.062	0.451	0.018494
250	SPP1	38.16	1.030	0.438	0.018610
250	TSC22D3	47.03	0.640	0.272	0.018697
250	PSMA5	229.7	-0.43	0.187	0.018701
250	PCNA	110.4	-0.51	0.217	0.018713
250	GPR68	10.54	-1.29	0.552	0.018806
250	NDUFA12	355.9	-0.43	0.185	0.019093
250	YIPF4	16.29	-1.03	0.440	0.019103
250	RNASE4	5.885	1.610	0.687	0.019143
250	C2orf68	12.19	1.067	0.455	0.019160
250	FDFT1	33.22	-0.74	0.319	0.019206
250	MYO1G	9.767	-1.27	0.544	0.019376
250	CCL4L1	10.94	1.143	0.489	0.019538
250	ITGA4	22.26	-0.84	0.360	0.019599
250	OTUB1	140.8	-0.45	0.196	0.019721
250	TSTD1	94.96	-0.52	0.226	0.019894
250	MGST2	49.53	-0.61	0.263	0.020168
250	SLAMF8	37.29	-0.74	0.319	0.020268
250	APRT	124.7	-0.45	0.195	0.020300
250	SLC9A3R1	38.12	-0.77	0.333	0.020321
250	TMSB10	4429.	-0.31	0.134	0.020472
250	CNTRL	16.92	-0.95	0.414	0.020597
250	GLUL	20.05	1.068	0.461	0.020696
250	LCP1	396.5	-0.37	0.161	0.020799
250	TMED10	176.6	0.448	0.193	0.020803
250	NDUFB8	177.5	-0.41	0.180	0.020807
250	HMOX2	99.86	-0.50	0.217	0.021054
250	C12orf75	58.75	-0.59	0.258	0.021164
250	GTF2H5	85.02	-0.53	0.230	0.021205
250	TMEM39A	24.61	0.764	0.331	0.021231
250	CLSPN	6.809	1.437	0.625	0.021520
250	SNHG16	52.07	0.593	0.259	0.021952
250	TMSB4X	12408	-0.39	0.174	0.022000
250	NAP1L1	226.0	0.520	0.227	0.022049
250	LPXN	184.5	-0.55	0.241	0.022142
250	P2RY10	30.38	0.693	0.303	0.022317
250	ZFAND2A	33.43	0.663	0.290	0.022529
250	TCF4	6.296	-1.60	0.702	0.022624
250	IL10RA	29.91	-0.77	0.340	0.022629
250	NFATC3	19.97	-0.91	0.402	0.022925
250	CDKN2B-AS1	1.284	3.450	1.517	0.022952
250	LETMD1	41.23	0.678	0.298	0.022989
250	DEDD2	13.05	-1.05	0.467	0.023756
250	HEXA	59.73	0.538	0.238	0.023943
250	CD3G	130.1	-0.49	0.217	0.024125
250	MTFP1	8.458	1.278	0.567	0.024273
250	FERMT3	152.1	-0.47	0.210	0.024299
250	XYLT1	4.622	-1.83	0.816	0.024318
250	SPINK2	25.88	-1.04	0.462	0.024373
250	CYSTM1	82.60	0.520	0.231	0.024381
250	ITGB2-AS1	7.801	-1.36	0.607	0.024512

250	LYSMD3	13.10	1.013	0.451	0.024682
250	CSDE1	165.0	0.455	0.202	0.024759
250	HNRNPM	15.79	-0.96	0.430	0.024797
250	RPS26	245.8	-0.41	0.183	0.024802
250	RFXANK	21.15	-0.87	0.388	0.024818
250	EBP	56.50	-0.61	0.276	0.025186
250	SAT2	44.58	0.617	0.275	0.025222
250	TCTA	4.614	-1.75	0.784	0.025550
250	PLPP6	1.368	-3.31	1.488	0.025720
250	SLC7A11	3.159	2.205	0.989	0.025828
250	APOBEC3F	12.64	-1.06	0.479	0.026014
250	NCDN	6.512	1.453	0.652	0.026052
250	GTF3C6	37.87	-0.66	0.296	0.026065
250	MRPL47	70.63	-0.52	0.237	0.026165
250	SKAP1	47.66	-0.71	0.322	0.026311
250	ACOT8	8.001	-1.51	0.683	0.026364
250	MAP3K13	19.69	-0.84	0.381	0.026423
250	SSBP1	153.0	-0.56	0.253	0.026447
250	OSER1	88.23	0.495	0.223	0.026531
250	CCDC163	8.136	1.172	0.529	0.026820
250	CEP63	14.73	0.904	0.409	0.027314
250	PSTPIP1	78.45	-0.66	0.301	0.027603
250	NEK1	21.38	0.796	0.361	0.027668
250	ANAPC15	11.37	-1.09	0.499	0.027783
250	ARRB1	8.638	-1.24	0.567	0.027803
250	THOC7	96.69	-0.47	0.214	0.028141
250	LOC374443	37.57	0.628	0.286	0.028144
250	RAB1B	50.94	-0.58	0.265	0.028454
250	MLEC	32.67	0.679	0.310	0.028488
250	IDNK	7.577	-1.33	0.610	0.028499
250	SLC35A4	33.66	-0.67	0.306	0.028636
250	NDUFA13	570.7	-0.32	0.148	0.028673
250	PNISR	149.7	0.501	0.229	0.028770
250	RGL4	26.33	-0.88	0.406	0.028786
250	CDC42	166.8	-0.42	0.192	0.028835
250	ERMP1	20.39	0.806	0.369	0.028956
250	HDLBP	37.17	0.641	0.294	0.029104
250	ATP2B4	58.48	-0.77	0.353	0.029130
250	CD69	44.45	-0.63	0.292	0.029137
250	PGK1	311.8	-0.39	0.178	0.029305
250	PSMA2	364.1	-0.35	0.163	0.029624
250	PNPLA6	3.002	2.134	0.981	0.029705
250	PLAAT3	38.61	-0.71	0.329	0.029713
250	PPP1CA	37.15	-0.64	0.295	0.029772
250	ITPRIPL2	4.399	-1.72	0.796	0.030063
250	STAU1	18.54	0.818	0.377	0.030157
250	FCMR	15.11	0.997	0.460	0.030164
250	OCIAD2	74.99	0.495	0.228	0.030350
250	ARPC3	270.5	-0.38	0.175	0.030455
250	USO1	17.83	0.837	0.387	0.030613
250	CTSC	528.1	-0.45	0.212	0.030798
250	AKR1A1	61.79	0.506	0.234	0.030902
250	DDX5	487.2	-0.43	0.203	0.030968
250	LAIR2	2.406	2.489	1.154	0.031033
250	RNF167	46.35	-0.59	0.274	0.031061
250	SIPA1L1	11.50	0.986	0.457	0.031140
250	RFX3	3.144	2.101	0.975	0.031326
250	TMEM116	10.87	1.026	0.477	0.031588
250	TMEM91	2.745	2.320	1.079	0.031650
250	RHOG	11.33	-1.19	0.555	0.031669
250	GTF2F2	19.27	0.782	0.364	0.031745
250	SKP2	2.869	-2.44	1.138	0.031791
250	PPM1M	5.722	-1.44	0.675	0.032136
250	LINC02273	6.194	-1.43	0.670	0.032238
250	SLC25A20	30.88	-0.69	0.325	0.032334
250	ATP6V0B	88.04	0.454	0.212	0.032446
250	RNF24	4.670	1.568	0.733	0.032519
250	FDX1	7.117	-1.38	0.646	0.032546
250	COQ8A	4.120	1.651	0.772	0.032580
250	ATP5PB	206.9	-0.40	0.189	0.032617
250	GPNMB	18.55	1.141	0.535	0.032860
250	NFIC	6.788	1.354	0.635	0.032923
250	UBB	742.8	-0.32	0.153	0.033221
250	HNRNPA2B1	162.8	-0.40	0.190	0.033626

250	APOBEC3G	146.9	-0.41	0.193	0.033652
250	SNHG1	21.85	0.856	0.403	0.033931
250	BIN2	52.64	-0.57	0.270	0.034019
250	HEMK1	8.947	-1.15	0.546	0.034157
250	UBASH3A	14.11	-0.94	0.448	0.034359
250	S100A6	993.3	-0.31	0.147	0.034387
250	SSR2	980.8	0.343	0.162	0.034540
250	UAP1	9.631	1.067	0.505	0.034759
250	SAP18	236.5	-0.39	0.185	0.034929
250	ITK	36.68	-0.67	0.321	0.035167
250	MDFIC	28.30	-0.77	0.366	0.035443
250	PPARA	4.557	1.588	0.755	0.035478
250	PTRHD1	37.14	-0.61	0.290	0.035506
250	CYBA	252.2	0.376	0.179	0.036091
250	LIPT1	13.34	0.925	0.442	0.036189
250	TMEM38B	8.972	1.261	0.602	0.036218
250	SEPTIN1	48.65	-0.55	0.263	0.036249
250	UPP1	44.93	0.561	0.268	0.036359
250	TNFRSF14-AS1	2.790	2.057	0.983	0.036465
250	DBNL	16.36	-0.88	0.424	0.036624
250	LYAR	17.11	0.873	0.418	0.036639
250	TUT4	13.55	1.001	0.479	0.036831
250	ASB2	13.87	-0.99	0.475	0.037085
250	NCF4	72.58	-0.54	0.261	0.037168
250	ARRDC1-AS1	4.535	1.618	0.776	0.037180
250	PUS10	1.167	3.276	1.573	0.037294
250	NSMAF	16.32	0.858	0.412	0.037355
250	SLC25A51	8.451	1.153	0.554	0.037621
250	UBXN1	73.05	0.466	0.224	0.037643
250	MMP25	55.34	-0.71	0.343	0.037645
250	TPTE2P5	2.363	2.683	1.290	0.037663
250	PSME1	491.6	-0.32	0.157	0.037823
250	EIF1	571.0	0.373	0.180	0.038036
250	SMC6	15.57	0.830	0.400	0.038243
250	CYB561D1	2.604	-2.16	1.048	0.038600
250	IMPDH2	68.81	0.510	0.246	0.038710
250	CCR1	15.44	-0.94	0.456	0.038786
250	PFDN1	96.76	-0.43	0.211	0.038882
250	SETD2	18.06	0.766	0.371	0.039035
250	ANP32B	32.55	0.651	0.315	0.039120
250	ELP6	23.35	-0.73	0.357	0.039280
250	RABGGTB	47.05	0.629	0.305	0.039357
250	CDKN2A	20.46	-0.81	0.394	0.039388
250	ACLY	17.42	-0.84	0.409	0.039403
250	LARS1	36.24	0.592	0.287	0.039418
250	ABCA7	9.848	1.077	0.523	0.039528
250	GYG1	105.9	-0.42	0.208	0.039717
250	TPST2	12.64	-1.07	0.523	0.039748
250	LINC00504	12.26	0.941	0.457	0.039783
250	RPL32P3	8.912	1.085	0.528	0.039815
250	CALR	115.0	0.410	0.200	0.040061
250	PDCL	26.52	-0.68	0.335	0.040093
250	NDUFA3	268.0	-0.36	0.177	0.040434
250	AP1AR	6.125	1.540	0.751	0.040503
250	CLIP4	10.42	1.141	0.557	0.040664
250	PSAP	336.6	0.422	0.206	0.040775
250	MOSPD3	4.437	-1.58	0.777	0.040921
250	CCDC167	96.88	0.449	0.220	0.041270
250	PSMB9	76.43	-0.48	0.237	0.041322
250	CEBPB	6.147	1.282	0.629	0.041442
250	IKZF3	6.808	-1.31	0.646	0.041534
250	VIM	242.7	0.592	0.290	0.041561
250	RTP4	13.70	-0.98	0.482	0.041697
250	SLC38A2	41.67	0.563	0.276	0.041708
250	LAMP2	139.2	0.452	0.222	0.041819
250	FCGRT	11.18	1.263	0.621	0.041862
250	CLEC2B	373.7	0.313	0.154	0.042152
250	TXNDC9	64.68	-0.49	0.243	0.042206
250	LIG1	5.120	1.426	0.702	0.042209
250	POP5	49.76	-0.53	0.264	0.042273
250	PHF3	24.86	0.662	0.326	0.042388
250	DHRS1	15.04	-0.94	0.464	0.042420
250	ERI1	14.65	-1.04	0.514	0.042602
250	DESI1	4.711	-1.59	0.784	0.042661

250	COX6C	508.0	-0.33	0.163	0.042860
250	FASTKD3	13.24	-0.99	0.489	0.042940
250	VAV1	2.770	-2.07	1.026	0.042963
250	DOK2	15.45	-0.92	0.455	0.042975
250	CHMP5	115.3	-0.45	0.226	0.043087
250	TRA2B	25.60	-0.68	0.338	0.043111
250	BRK1	296.3	-0.45	0.224	0.043282
250	GZMA	2778.	-0.43	0.215	0.043346
250	MAP2K1	10.64	-1.05	0.522	0.043347
250	NDFIP2	92.03	-0.47	0.234	0.043494
250	MEI1	43.23	0.586	0.290	0.043815
250	ARID1A	8.812	-1.12	0.559	0.043818
250	SLC35B1	26.09	0.648	0.322	0.044305
250	STX5	1.492	2.683	1.335	0.044411
250	JAK3	17.13	-0.87	0.434	0.044573
250	THAP11	7.800	-1.23	0.613	0.044582
250	MAP4K4	11.89	0.980	0.488	0.044733
250	TRAF3IP3	111.6	-0.48	0.242	0.044822
250	RHOA	395.1	-0.45	0.224	0.044867
250	CCDC88B	7.309	1.207	0.601	0.044916
250	LFNG	4.402	-1.69	0.845	0.044948
250	MVP	7.942	-1.24	0.622	0.044986
250	GRAP2	25.01	-0.72	0.360	0.044992
250	SLFN5	78.99	-0.49	0.245	0.045063
250	LMAN1	81.90	0.476	0.237	0.045166
250	POLH	20.19	-0.75	0.377	0.045192
250	C1orf54	5.787	1.459	0.729	0.045329
250	GBP2	82.17	0.590	0.295	0.045365
250	PMF1	14.24	-0.87	0.438	0.045441
250	GINM1	11.82	0.923	0.461	0.045581
250	STX3	32.42	-0.61	0.308	0.046017
250	RAB29	17.88	-0.80	0.403	0.046092
250	MRPL37	10.12	-1.10	0.554	0.046330
250	C1RL-AS1	2.564	1.976	0.992	0.046333
250	TRPM7	9.325	1.016	0.510	0.046387
250	ATP5IF1	125.9	-0.41	0.207	0.046516
250	ADK	23.98	0.717	0.360	0.046607
250	C15orf61	9.755	0.984	0.495	0.046942
250	TOP1	17.65	-0.80	0.404	0.046951
250	PACC1	5.075	-1.49	0.751	0.046998
250	HPS1	12.81	0.944	0.475	0.047027
250	POGLUT3	1.993	2.397	1.207	0.047160
250	RBM39	121.5	-0.39	0.198	0.047339
250	SRSF7	111.6	-0.40	0.204	0.047442
250	PRRC2C	104.2	0.453	0.229	0.047515
250	AIM2	3.036	2.255	1.138	0.047531
250	RAB1A	19.07	0.746	0.377	0.047659
250	MRPL13	84.36	-0.44	0.225	0.047744
250	C16orf54	13.26	-0.90	0.458	0.047786
250	TPM3	158.7	-0.41	0.209	0.047824
250	TMBIM4	246.3	-0.36	0.186	0.047879
250	CA2	7.013	1.872	0.946	0.047879
250	BRCC3	8.254	-1.15	0.584	0.047982
250	TMEM30A	40.22	-0.56	0.286	0.047987
250	PPP2R3B	1.915	2.379	1.203	0.048061
250	KIAA1109	8.792	1.080	0.546	0.048182
250	SIX4	2.977	1.742	0.882	0.048215
250	SHLD2	3.244	1.735	0.878	0.048221
250	ZNF317	16.04	0.802	0.406	0.048308
250	ITM2B	150.4	-0.41	0.209	0.048635
250	PPP1R15A	8.671	1.039	0.527	0.048677
250	CYB5B	42.88	-0.60	0.307	0.048704
250	SIT1	11.41	-1.15	0.587	0.048923
250	TENT5C	23.82	0.681	0.346	0.049063
250	ETS1	37.51	-0.63	0.321	0.049246
250	ARPC4	66.38	-0.46	0.237	0.049267
250	PELATON	5.781	2.042	1.039	0.049513
250	BANF1	100.5	-0.41	0.213	0.049791
250	DANCR	39.83	0.587	0.299	0.049847
250	PTPN6	17.70	-0.82	0.418	0.049905
