# Supplementary material for: The DCMU Herbicide Shapes T-cell Functions By Modulating Micro-RNA Expression Profiles
Source: Front Immunol. 2022 Jul 28;13:925241. doi: 10.3389/fimmu.2022.925241 (PMC9366666; doi:10.3389/fimmu.2022.925241)

Supplemental Figure 1. KEGG pathways significantly enriched in DEmRNA after DCMU exposures. **(A)** 10μM, **(B)** 100μM, **(C)** 250μM.

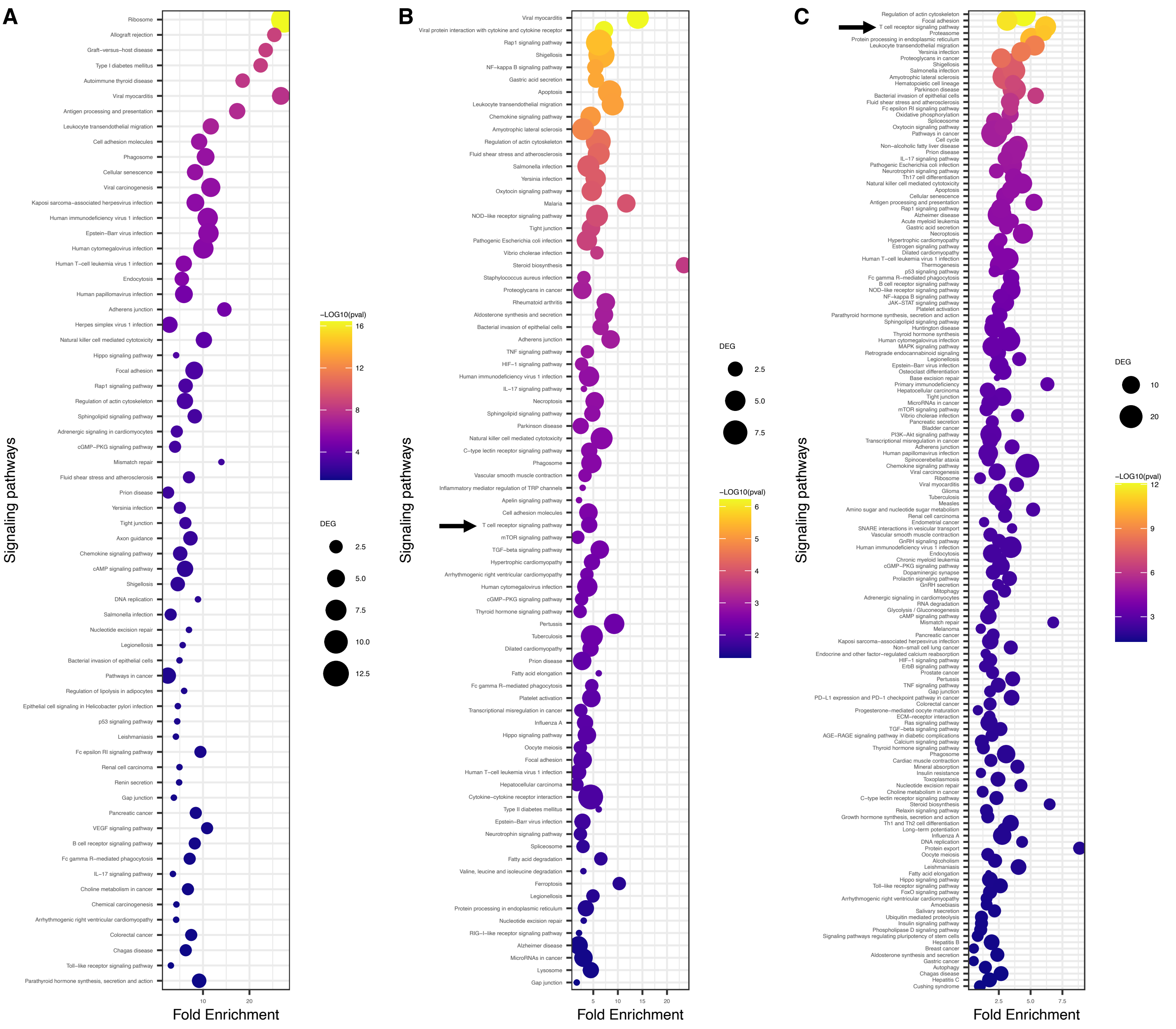

Supplemental Figure 2. Volcano plot of genes involved in the miRNA biogenesis.

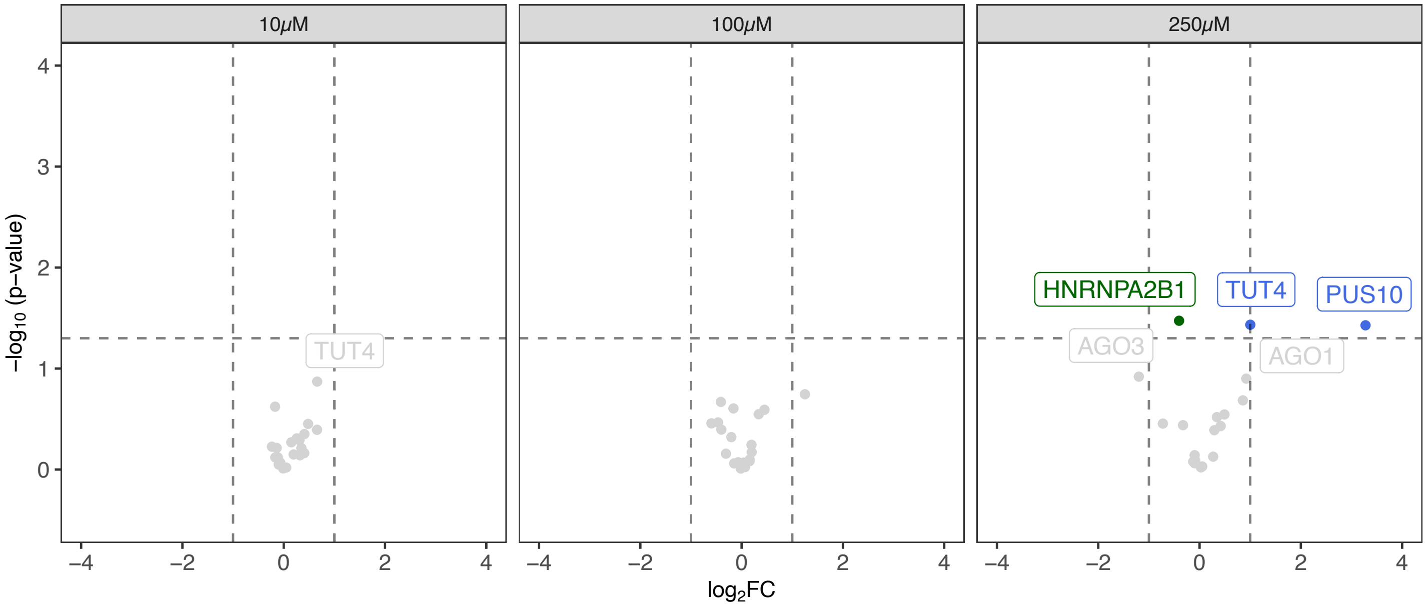

Supplemental Figure 3. Negative controls of cytotoxicity assays. **(A)** Cytotoxicity assays against M6 cells by CTL03.1 cells. M6 cells are melanoma cell lines as well but not recognized by the HLA-A*02 restricted CTL03.1 cells. **(B)** Cytotoxicity assays against Meso13 cells by N5.14 cells. Again, N5.14 cells cannot recognize Meso13 cells due to their HLA restriction. In black CTL03.1 cells exposed to DCMU, in grey CTL03.1 cells exposed to vehicle only. Each time 3 independent experiments were run in triplicate per condition.

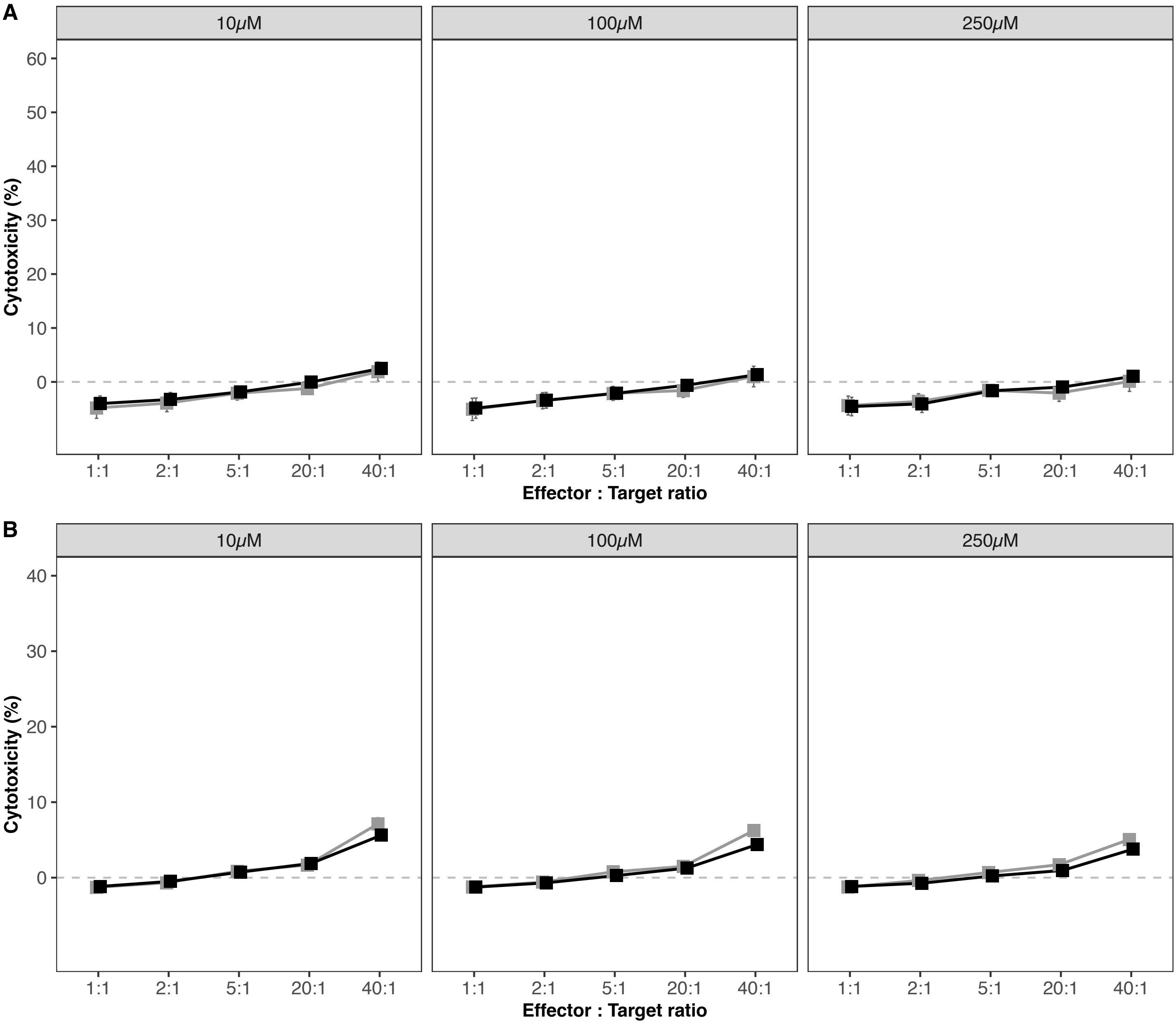

Supplemental Figure 4. (A) IFN-γ protein concentrations in supernatant of CTL03.1 cells transfected with candidate miRNA mimics or antimiR during 24h. **(B)** TNF-α protein concentrations in supernatant of CTL03.1 cells transfected with candidate miRNA mimics or antimiR during 24h. **(C)** Gr-B protein concentrations in supernatant of CTL03.1 cells transfected with candidate miRNA mimics or antimiR during 24h.

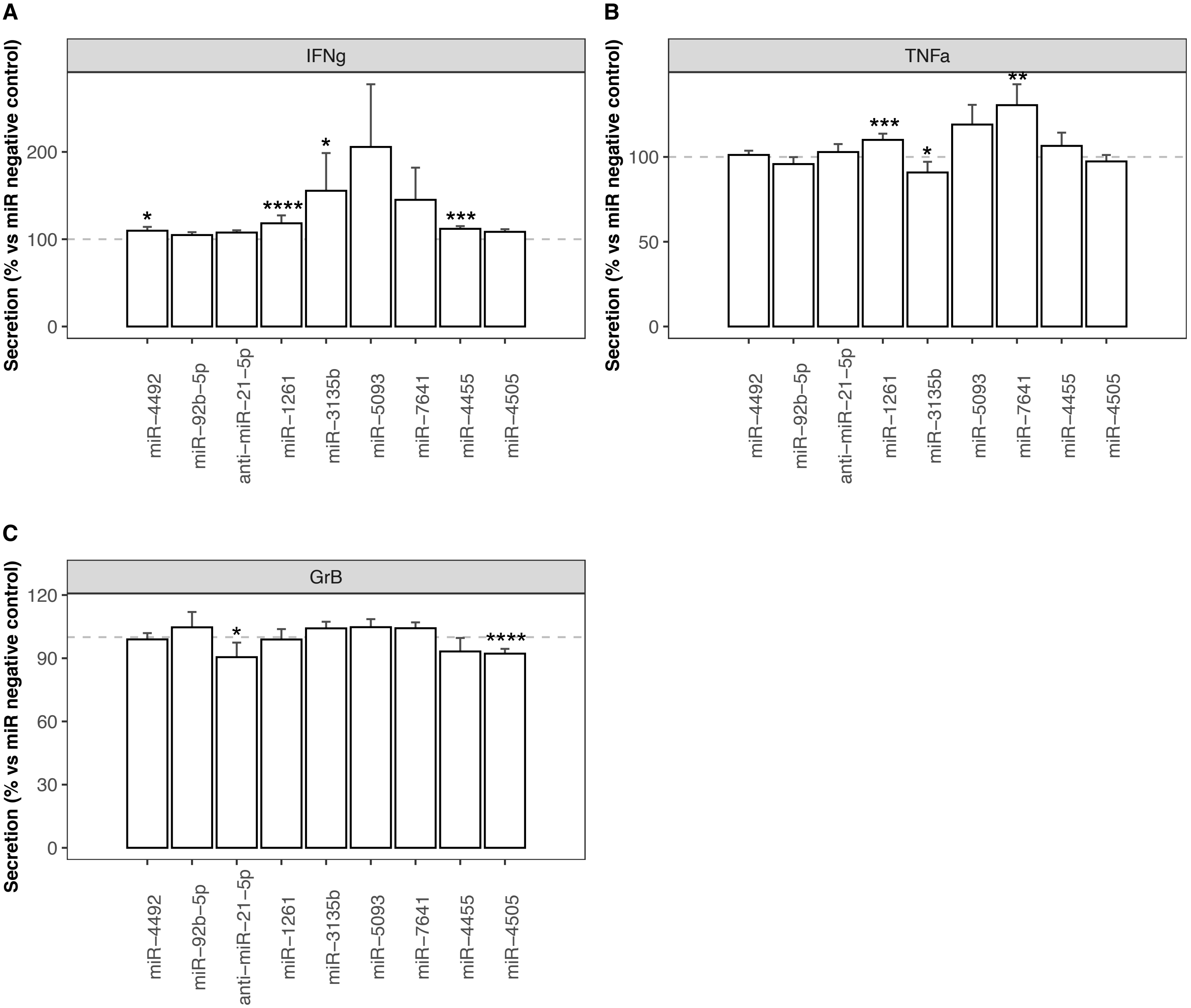

Supplemental Figure 5. (A) hsa-miR-3135b expression in CTL03.1 cells exposed to DCMU or DMSO during 24h **(B)** hsa-miR-21-3p expression in CTL03.1 cells exposed to DCMU or DMSO during 24h**(C)** Gr-B protein concentrations in supernatant of CTL03.1 cells transfected with candidate miRNA mimics or antimiR during 24h. (**C)** Quantitative real-time PCR expression levels of XLC1 in CTL03.1 transfected with increasing doses of miR-3135b mimic.

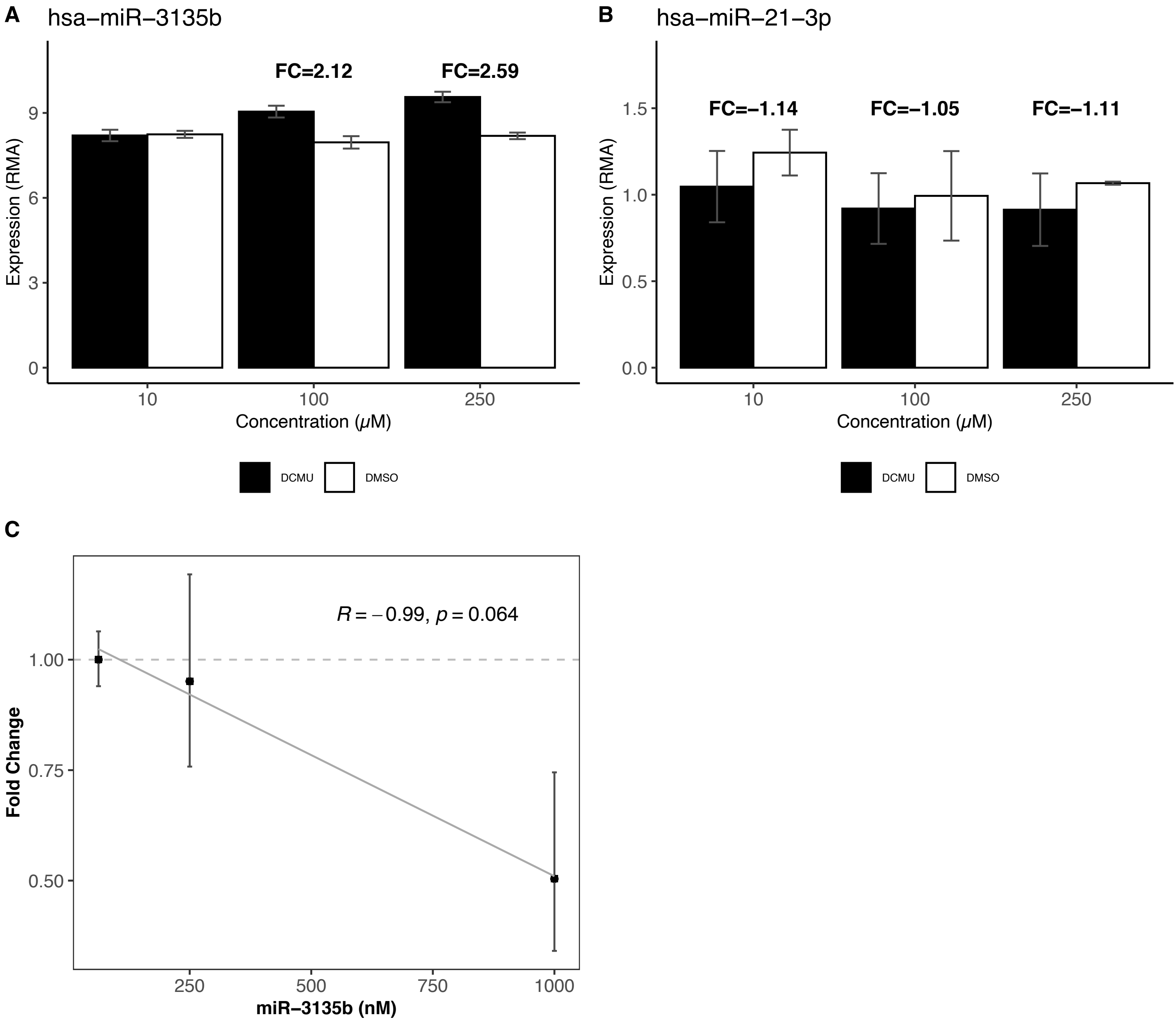

Supplement: Supplementary file 4 [file DataSheet_4.pdf]
