# Supplementary material for: The DCMU Herbicide Shapes T-cell Functions By Modulating Micro-RNA Expression Profiles
Source: Front Immunol. 2022 Jul 28;13:925241. doi: 10.3389/fimmu.2022.925241 (PMC9366666; doi:10.3389/fimmu.2022.925241)
Supplement: Supplementary file 5 [file DataSheet_5.pdf]

Supplemental Table 4. Cytokine concentrations in response to DCMU or DMSO exposures.

Cytokine	T Cell	Treatment	Treatment Concentration (μM)	Cytokine Concentration (pg/mL)	Experiment reference	Exposure
GrB	CD8pol	DMSO	250	6712.94	200710	24h
GrB	CD8pol	DMSO	100	9442.35	200710	24h
GrB	CD8pol	DMSO	10	11818.82	200710	24h
GrB	CD8pol	DCMU	250	13418.82	200710	24h
GrB	CD8pol	DCMU	100	9912.94	200710	24h
GrB	CD8pol	DCMU	10	12501.17	200710	24h
GrB	CD8pol	DMSO	250	7677.64	200710	24h
GrB	CD8pol	DMSO	100	10360	200710	24h
GrB	CD8pol	DMSO	10	12312.94	200710	24h
GrB	CD8pol	DCMU	250	14524.70	200710	24h
GrB	CD8pol	DCMU	100	11254.11	200710	24h
GrB	CD8pol	DCMU	10	12642.35	200710	24h
GrB	CD8pol	DMSO	250	7912.94	200710	24h
GrB	CD8pol	DMSO	100	10524.70	200710	24h
GrB	CD8pol	DMSO	10	13230.58	200710	24h
GrB	CD8pol	DCMU	250	14265.88	200710	24h
GrB	CD8pol	DCMU	100	11348.23	200710	24h
GrB	CD8pol	DCMU	10	12665.88	200710	24h
GrB	CD8pol	DMSO	250	4399.45	200715	24h
GrB	CD8pol	DMSO	100	6491.35	200715	24h
GrB	CD8pol	DMSO	10	8356.21	200715	24h
GrB	CD8pol	DCMU	250	8848.10	200715	24h
GrB	CD8pol	DCMU	100	6858.91	200715	24h
GrB	CD8pol	DCMU	10	7821.08	200715	24h
GrB	CD8pol	DMSO	250	4507.56	200715	24h
GrB	CD8pol	DMSO	100	6388.64	200715	24h
GrB	CD8pol	DMSO	10	8696.75	200715	24h
GrB	CD8pol	DCMU	250	9048.10	200715	24h
GrB	CD8pol	DCMU	100	7064.32	200715	24h
GrB	CD8pol	DCMU	10	8329.18	200715	24h
GrB	CD8pol	DMSO	250	4685.94	200715	24h
GrB	CD8pol	DMSO	100	6307.56	200715	24h
GrB	CD8pol	DMSO	10	8485.94	200715	24h
GrB	CD8pol	DCMU	250	9080.54	200715	24h
GrB	CD8pol	DCMU	100	7042.70	200715	24h
GrB	CD8pol	DCMU	10	8280.54	200715	24h
GrB	CD8pol	DMSO	250	6804.86	200715	D5
GrB	CD8pol	DMSO	100	6210.27	200715	D5
GrB	CD8pol	DMSO	10	8161.62	200715	D5
GrB	CD8pol	DCMU	250	6296.75	200715	D5
GrB	CD8pol	DCMU	100	6821.08	200715	D5
GrB	CD8pol	DCMU	10	8091.35	200715	D5
GrB	CD8pol	DMSO	250	7167.02	200715	D5
GrB	CD8pol	DMSO	100	6356.21	200715	D5
GrB	CD8pol	DMSO	10	8123.78	200715	D5
GrB	CD8pol	DCMU	250	6404.86	200715	D5
GrB	CD8pol	DCMU	100	7215.67	200715	D5
GrB	CD8pol	DCMU	10	8031.89	200715	D5
GrB	CD8pol	DMSO	250	7437.29	200715	D5

GrB	CD8pol	DMSO	100	6475.13	200715	D5
GrB	CD8pol	DMSO	10	8210.27	200715	D5
GrB	CD8pol	DCMU	250	7183.24	200715	D5
GrB	CD8pol	DCMU	100	6831.89	200715	D5
GrB	CD8pol	DCMU	10	8485.94	200715	D5
GrB	CD8pol	DMSO	250	21679.36	201127	D5
GrB	CD8pol	DMSO	100	20028.57	201127	D5
GrB	CD8pol	DMSO	10	20663.49	201127	D5
GrB	CD8pol	DCMU	250	4409.52	201127	D5
GrB	CD8pol	DCMU	100	18219.04	201127	D5
GrB	CD8pol	DCMU	10	19488.88	201127	D5
GrB	CD8pol	DMSO	250	21965.07	201127	D5
GrB	CD8pol	DMSO	100	20219.04	201127	D5
GrB	CD8pol	DMSO	10	20092.06	201127	D5
GrB	CD8pol	DCMU	250	4536.50	201127	D5
GrB	CD8pol	DCMU	100	18758.73	201127	D5
GrB	CD8pol	DCMU	10	22155.55	201127	D5
GrB	CD8pol	DMSO	250	19488.88	201127	D5
GrB	CD8pol	DMSO	100	20853.96	201127	D5
GrB	CD8pol	DMSO	10	27647.61	201127	D5
GrB	CD8pol	DCMU	250	2123.80	201127	D5
GrB	CD8pol	DCMU	100	14123.80	201127	D5
GrB	CD8pol	DCMU	10	24219.04	201127	D5
GrB	CD8pol	DMSO	250	18790.47	201127	D5
GrB	CD8pol	DMSO	100	21107.93	201127	D5
GrB	CD8pol	DMSO	10	27584.12	201127	D5
GrB	CD8pol	DCMU	250	2250.79	201127	D5
GrB	CD8pol	DCMU	100	14790.47	201127	D5
GrB	CD8pol	DCMU	10	23552.38	201127	D5
GrB	CD8pol	DMSO	250	3107.93	201127	D5
GrB	CD8pol	DMSO	100	3996.82	201127	D5
GrB	CD8pol	DMSO	10	3203.17	201127	D5
GrB	CD8pol	DCMU	250	2726.98	201127	D5
GrB	CD8pol	DCMU	100	2822.22	201127	D5
GrB	CD8pol	DCMU	10	2663.49	201127	D5
GrB	CD8pol	DMSO	250	2853.96	201127	D5
GrB	CD8pol	DMSO	100	3266.66	201127	D5
GrB	CD8pol	DMSO	10	2536.50	201127	D5
GrB	CD8pol	DCMU	250	2790.47	201127	D5
GrB	CD8pol	DCMU	100	3012.69	201127	D5
GrB	CD8pol	DCMU	10	3076.19	201127	D5
GrB	CD8pol	DMSO	250	289.55	201201	24h
GrB	CD8pol	DMSO	100	498.50	201201	24h
GrB	CD8pol	DMSO	10	677.61	201201	24h
GrB	CD8pol	DCMU	100	110.44	201201	24h
GrB	CD8pol	DCMU	10	274.62	201201	24h
GrB	CD8pol	DMSO	250	304.47	201201	24h
GrB	CD8pol	DMSO	100	438.80	201201	24h
GrB	CD8pol	DMSO	10	752.23	201201	24h
GrB	CD8pol	DCMU	100	110.44	201201	24h
GrB	CD8pol	DCMU	10	274.62	201201	24h
GrB	CD8pol	DMSO	250	334.32	201201	24h

GrB	CD8pol	DMSO	100	886.56	201201	24h
GrB	CD8pol	DMSO	10	1095.52	201201	24h
GrB	CD8pol	DCMU	100	20.89	201201	24h
GrB	CD8pol	DCMU	10	632.83	201201	24h
GrB	CD8pol	DMSO	250	334.32	201201	24h
GrB	CD8pol	DMSO	100	632.83	201201	24h
GrB	CD8pol	DMSO	10	901.49	201201	24h
GrB	CD8pol	DCMU	10	588.05	201201	24h
GrB	CD8pol	DMSO	250	768.57	201208	24h
GrB	CD8pol	DMSO	100	854.28	201208	24h
GrB	CD8pol	DMSO	10	854.28	201208	24h
GrB	CD8pol	DCMU	250	654.28	201208	24h
GrB	CD8pol	DCMU	100	711.42	201208	24h
GrB	CD8pol	DCMU	10	997.14	201208	24h
GrB	CD8pol	DMSO	250	825.71	201208	24h
GrB	CD8pol	DMSO	100	854.28	201208	24h
GrB	CD8pol	DMSO	10	882.85	201208	24h
GrB	CD8pol	DCMU	250	625.71	201208	24h
GrB	CD8pol	DCMU	100	711.42	201208	24h
GrB	CD8pol	DCMU	10	997.14	201208	24h
GrB	CD8pol	DMSO	250	25340	201208	D5
GrB	CD8pol	DMSO	100	24597.14	201208	D5
GrB	CD8pol	DMSO	10	24911.42	201208	D5
GrB	CD8pol	DCMU	250	16368.57	201208	D5
GrB	CD8pol	DCMU	100	19540	201208	D5
GrB	CD8pol	DCMU	10	24311.42	201208	D5
GrB	CD8pol	DMSO	250	25540	201208	D5
GrB	CD8pol	DMSO	100	24511.42	201208	D5
GrB	CD8pol	DMSO	10	24968.57	201208	D5
GrB	CD8pol	DCMU	250	16397.14	201208	D5
GrB	CD8pol	DCMU	100	18797.14	201208	D5
GrB	CD8pol	DCMU	10	23568.57	201208	D5
GrB	CD8pol	DMSO	250	4399.5	200715	24h
GrB	CD8pol	DMSO	100	6491.4	200715	24h
GrB	CD8pol	DMSO	10	8356.2	200715	24h
GrB	CD8pol	DCMU	250	8848.1	200715	24h
GrB	CD8pol	DCMU	100	6858.9	200715	24h
GrB	CD8pol	DCMU	10	7821.1	200715	24h
GrB	CD8pol	DMSO	250	4507.6	200715	24h
GrB	CD8pol	DMSO	100	6388.6	200715	24h
GrB	CD8pol	DMSO	10	8696.8	200715	24h
GrB	CD8pol	DCMU	250	9048.1	200715	24h
GrB	CD8pol	DCMU	100	7064.3	200715	24h
GrB	CD8pol	DCMU	10	8329.2	200715	24h
GrB	CD8pol	DMSO	250	4685.9	200715	24h
GrB	CD8pol	DMSO	100	6307.6	200715	24h
GrB	CD8pol	DMSO	10	8485.9	200715	24h
GrB	CD8pol	DCMU	250	9080.5	200715	24h
GrB	CD8pol	DCMU	100	7042.7	200715	24h
GrB	CD8pol	DCMU	10	8280.5	200715	24h
GrB	CD8pol	DMSO	250	6804.9	200715	D5
GrB	CD8pol	DMSO	100	6210.3	200715	D5

GrB	CD8pol	DMSO	10	8161.6	200715	D5
GrB	CD8pol	DCMU	250	6296.8	200715	D5
GrB	CD8pol	DCMU	100	6821.1	200715	D5
GrB	CD8pol	DCMU	10	8091.4	200715	D5
GrB	CD8pol	DMSO	250	7167	200715	D5
GrB	CD8pol	DMSO	100	6356.2	200715	D5
GrB	CD8pol	DMSO	10	8123.8	200715	D5
GrB	CD8pol	DCMU	250	6404.9	200715	D5
GrB	CD8pol	DCMU	100	7215.7	200715	D5
GrB	CD8pol	DCMU	10	8031.9	200715	D5
GrB	CD8pol	DMSO	250	7437.3	200715	D5
GrB	CD8pol	DMSO	100	6475.1	200715	D5
GrB	CD8pol	DMSO	10	8210.3	200715	D5
GrB	CD8pol	DCMU	250	7183.2	200715	D5
GrB	CD8pol	DCMU	100	6831.9	200715	D5
GrB	CD8pol	DCMU	10	8485.9	200715	D5
GrB	CD8pol	DMSO	250	289.55	201201	24h
GrB	CD8pol	DMSO	100	498.50	201201	24h
GrB	CD8pol	DMSO	10	677.61	201201	24h
GrB	CD8pol	DCMU	100	110.44	201201	24h
GrB	CD8pol	DCMU	10	274.62	201201	24h
GrB	CD8pol	DMSO	250	304.47	201201	24h
GrB	CD8pol	DMSO	100	438.80	201201	24h
GrB	CD8pol	DMSO	10	752.23	201201	24h
GrB	CD8pol	DCMU	100	110.44	201201	24h
GrB	CD8pol	DCMU	10	274.62	201201	24h
GrB	CD8pol	DMSO	250	334.32	201201	24h
GrB	CD8pol	DMSO	100	886.56	201201	24h
GrB	CD8pol	DMSO	10	1095.52	201201	24h
GrB	CD8pol	DCMU	100	20.89	201201	24h
GrB	CD8pol	DCMU	10	632.83	201201	24h
GrB	CD8pol	DMSO	250	334.32	201201	24h
GrB	CD8pol	DMSO	100	632.83	201201	24h
GrB	CD8pol	DMSO	10	901.49	201201	24h
GrB	CD8pol	DCMU	10	588.05	201201	24h
IL2	CD8pol	DMSO	250	7741.66	200615	24h
IL2	CD8pol	DMSO	100	8175	200615	24h
IL2	CD8pol	DMSO	10	7541.66	200615	24h
IL2	CD8pol	DCMU	250	8025	200615	24h
IL2	CD8pol	DCMU	100	7891.66	200615	24h
IL2	CD8pol	DCMU	10	7225	200615	24h
IL2	CD8pol	DMSO	250	7608.33	200615	24h
IL2	CD8pol	DMSO	100	8125	200615	24h
IL2	CD8pol	DMSO	10	8041.66	200615	24h
IL2	CD8pol	DCMU	250	7275	200615	24h
IL2	CD8pol	DCMU	100	7975	200615	24h
IL2	CD8pol	DCMU	10	7058.33	200615	24h
IL2	CD8pol	DMSO	250	7358.33	200615	24h
IL2	CD8pol	DMSO	100	8125	200615	24h
IL2	CD8pol	DMSO	10	7575	200615	24h
IL2	CD8pol	DCMU	250	7325	200615	24h
IL2	CD8pol	DCMU	100	7941.66	200615	24h

IL2	CD8pol	DCMU	10	7425	200615	24h
IL2	CD8pol	DMSO	250	8791.66	200615	24h
IL2	CD8pol	DMSO	100	8725	200615	24h
IL2	CD8pol	DMSO	10	9791.66	200615	24h
IL2	CD8pol	DCMU	250	8808.33	200615	24h
IL2	CD8pol	DCMU	100	8908.33	200615	24h
IL2	CD8pol	DCMU	10	8225	200615	24h
IL2	CD8pol	DMSO	250	8525	200615	24h
IL2	CD8pol	DMSO	100	8541.66	200615	24h
IL2	CD8pol	DMSO	10	9258.33	200615	24h
IL2	CD8pol	DCMU	250	8575	200615	24h
IL2	CD8pol	DCMU	100	8658.33	200615	24h
IL2	CD8pol	DCMU	10	8825	200615	24h
IL2	CD8pol	DMSO	250	7775	200615	24h
IL2	CD8pol	DMSO	100	8391.66	200615	24h
IL2	CD8pol	DMSO	10	9291.66	200615	24h
IL2	CD8pol	DCMU	250	8391.66	200615	24h
IL2	CD8pol	DCMU	100	8675	200615	24h
IL2	CD8pol	DCMU	10	8208.33	200615	24h
IL2	CD8pol	DMSO	250	16293	191217	24h
IL2	CD8pol	DMSO	100	16113	191217	24h
IL2	CD8pol	DMSO	10	16563	191217	24h
IL2	CD8pol	DCMU	250	21363	191217	24h
IL2	CD8pol	DCMU	100	18453	191217	24h
IL2	CD8pol	DCMU	10	20673	191217	24h
IL2	CD8pol	DMSO	250	15753	191217	24h
IL2	CD8pol	DMSO	100	15483	191217	24h
IL2	CD8pol	DMSO	10	15123	191217	24h
IL2	CD8pol	DCMU	250	19353	191217	24h
IL2	CD8pol	DCMU	100	17823	191217	24h
IL2	CD8pol	DCMU	10	19893	191217	24h
IL2	CD8pol	DMSO	250	14523	191217	24h
IL2	CD8pol	DMSO	100	14943	191217	24h
IL2	CD8pol	DCMU	250	19653	191217	24h
IL2	CD8pol	DCMU	100	16893	191217	24h
IL2	CD8pol	DCMU	10	18663	191217	24h
IL2	CD8pol	DMSO	250	13803	191217	24h
IL2	CD8pol	DMSO	100	14253	191217	24h
IL2	CD8pol	DMSO	10	14643	191217	24h
IL2	CD8pol	DCMU	250	18363	191217	24h
IL2	CD8pol	DCMU	100	16053	191217	24h
IL2	CD8pol	DCMU	10	17883	191217	24h
IL2	CD8pol	DMSO	250	13833	191217	24h
IL2	CD8pol	DMSO	100	14163	191217	24h
IL2	CD8pol	DMSO	10	15213	191217	24h
IL2	CD8pol	DCMU	250	18483	191217	24h
IL2	CD8pol	DCMU	100	16173	191217	24h
IL2	CD8pol	DCMU	10	17523	191217	24h
IL2	CD8pol	DMSO	250	14013	191217	24h
IL2	CD8pol	DMSO	100	14583	191217	24h
IL2	CD8pol	DMSO	10	14523	191217	24h
IL2	CD8pol	DCMU	250	18123	191217	24h

IL2	CD8pol	DCMU	100	15813	191217	24h
IL2	CD8pol	DCMU	10	17583	191217	24h
IFNγ	CD8pol	DMSO	250	6557.14	200615	24h
IFNγ	CD8pol	DMSO	100	6450	200615	24h
IFNγ	CD8pol	DMSO	10	6592.85	200615	24h
IFNγ	CD8pol	DCMU	250	3378.57	200615	24h
IFNγ	CD8pol	DCMU	100	5092.85	200615	24h
IFNγ	CD8pol	DCMU	10	7057.14	200615	24h
IFNγ	CD8pol	DMSO	250	5842.85	200615	24h
IFNγ	CD8pol	DMSO	100	7807.14	200615	24h
IFNγ	CD8pol	DMSO	10	3842.85	200615	24h
IFNγ	CD8pol	DCMU	250	2378.57	200615	24h
IFNγ	CD8pol	DCMU	100	3271.42	200615	24h
IFNγ	CD8pol	DCMU	10	5664.28	200615	24h
IFNγ	CD8pol	DMSO	250	4771.42	200615	24h
IFNγ	CD8pol	DMSO	100	3950	200615	24h
IFNγ	CD8pol	DMSO	10	4271.42	200615	24h
IFNγ	CD8pol	DCMU	250	3307.14	200615	24h
IFNγ	CD8pol	DCMU	100	2878.57	200615	24h
IFNγ	CD8pol	DCMU	10	4128.57	200615	24h
IFNγ	CD8pol	DMSO	250	2128.57	200615	24h
IFNγ	CD8pol	DMSO	100	2235.71	200615	24h
IFNγ	CD8pol	DMSO	10	2985.71	200615	24h
IFNγ	CD8pol	DCMU	250	1450	200615	24h
IFNγ	CD8pol	DCMU	100	1914.28	200615	24h
IFNγ	CD8pol	DCMU	10	2021.42	200615	24h
IFNγ	CD8pol	DMSO	250	2271.42	200615	24h
IFNγ	CD8pol	DMSO	100	2092.85	200615	24h
IFNγ	CD8pol	DMSO	10	1735.71	200615	24h
IFNγ	CD8pol	DCMU	250	1842.85	200615	24h
IFNγ	CD8pol	DCMU	100	1664.28	200615	24h
IFNγ	CD8pol	DCMU	10	2485.71	200615	24h
IFNγ	CD8pol	DMSO	250	2271.42	200615	24h
IFNγ	CD8pol	DMSO	100	2735.71	200615	24h
IFNγ	CD8pol	DMSO	10	1878.57	200615	24h
IFNγ	CD8pol	DCMU	250	2592.85	200615	24h
IFNγ	CD8pol	DCMU	100	1878.57	200615	24h
IFNγ	CD8pol	DCMU	10	1985.71	200615	24h
TNFα	CD8pol	DMSO	250	141.55	200519	24h
TNFα	CD8pol	DMSO	100	133.12	200519	24h
TNFα	CD8pol	DMSO	10	151.03	200519	24h
TNFα	CD8pol	DCMU	250	48.76	200519	24h
TNFα	CD8pol	DCMU	100	100.95	200519	24h
TNFα	CD8pol	DCMU	10	135.68	200519	24h
TNFα	CD8pol	DMSO	250	113.90	200519	24h
TNFα	CD8pol	DMSO	100	122.53	200519	24h
TNFα	CD8pol	DMSO	10	101.99	200519	24h
TNFα	CD8pol	DCMU	250	33.87	200519	24h
TNFα	CD8pol	DCMU	100	81.11	200519	24h
TNFα	CD8pol	DCMU	10	120.54	200519	24h
TNFα	CD8pol	DMSO	250	118.76	200519	24h
TNFα	CD8pol	DMSO	100	122.58	200519	24h

TNFα	CD8pol	DMSO	10	122.79	200519	24h
TNFα	CD8pol	DCMU	250	35.58	200519	24h
TNFα	CD8pol	DCMU	100	87.47	200519	24h
TNFα	CD8pol	DCMU	10	129.27	200519	24h
TNFα	CD8pol	DMSO	250	415.40	200205	24h
TNFα	CD8pol	DMSO	100	458.78	200205	24h
TNFα	CD8pol	DMSO	10	422.38	200205	24h
TNFα	CD8pol	DCMU	250	118.71	200205	24h
TNFα	CD8pol	DCMU	100	360.25	200205	24h
TNFα	CD8pol	DCMU	10	508.78	200205	24h
TNFα	CD8pol	DMSO	250	462.09	200205	24h
TNFα	CD8pol	DMSO	100	446.65	200205	24h
TNFα	CD8pol	DMSO	10	467.24	200205	24h
TNFα	CD8pol	DCMU	250	150.33	200205	24h
TNFα	CD8pol	DCMU	100	302.16	200205	24h
TNFα	CD8pol	DCMU	10	502.53	200205	24h
TNFα	CD8pol	DMSO	250	422.02	200205	24h
TNFα	CD8pol	DMSO	100	422.02	200205	24h
TNFα	CD8pol	DMSO	10	433.78	200205	24h
TNFα	CD8pol	DCMU	250	156.58	200205	24h
TNFα	CD8pol	DCMU	100	320.18	200205	24h
TNFα	CD8pol	DCMU	10	454.74	200205	24h
TNFα	CD8pol	DMSO	250	959.85	200206	24h
TNFα	CD8pol	DMSO	100	1055.63	200206	24h
TNFα	CD8pol	DMSO	10	1007.74	200206	24h
TNFα	CD8pol	DCMU	250	916.90	200206	24h
TNFα	CD8pol	DCMU	100	1054.22	200206	24h
TNFα	CD8pol	DCMU	10	959.15	200206	24h
TNFα	CD8pol	DMSO	250	864.08	200206	24h
TNFα	CD8pol	DMSO	100	1026.05	200206	24h
TNFα	CD8pol	DMSO	10	928.16	200206	24h
TNFα	CD8pol	DCMU	250	768.30	200206	24h
TNFα	CD8pol	DCMU	100	1023.94	200206	24h
TNFα	CD8pol	DCMU	10	930.28	200206	24h
TNFα	CD8pol	DMSO	250	807.74	200206	24h
TNFα	CD8pol	DMSO	100	902.11	200206	24h
TNFα	CD8pol	DMSO	10	853.52	200206	24h
TNFα	CD8pol	DCMU	250	664.08	200206	24h
TNFα	CD8pol	DCMU	100	933.09	200206	24h
TNFα	CD8pol	DCMU	10	868.30	200206	24h
TNFα	CD8pol	DMSO	250	286.41	200309	24h
TNFα	CD8pol	DMSO	100	288.33	200309	24h
TNFα	CD8pol	DMSO	10	355.64	200309	24h
TNFα	CD8pol	DCMU	250	40.89	200309	24h
TNFα	CD8pol	DCMU	100	218.46	200309	24h
TNFα	CD8pol	DCMU	10	305	200309	24h
TNFα	CD8pol	DMSO	250	251.15	200309	24h
TNFα	CD8pol	DMSO	100	302.43	200309	24h
TNFα	CD8pol	DMSO	10	276.79	200309	24h
TNFα	CD8pol	DCMU	250	10.76	200309	24h
TNFα	CD8pol	DCMU	100	171.66	200309	24h
TNFα	CD8pol	DCMU	10	308.84	200309	24h

TNFα	CD8pol	DMSO	250	254.35	200309	24h
TNFα	CD8pol	DMSO	100	321.66	200309	24h
TNFα	CD8pol	DMSO	10	303.71	200309	24h
TNFα	CD8pol	DCMU	250	16.53	200309	24h
TNFα	CD8pol	DCMU	100	184.48	200309	24h
TNFα	CD8pol	DCMU	10	323.58	200309	24h
IFNγ	CTL03.1	DMSO	250	132.82	200901	24h
IFNγ	CTL03.1	DMSO	100	124.13	200901	24h
IFNγ	CTL03.1	DMSO	10	85	200901	24h
IFNγ	CTL03.1	DCMU	250	57.82	200901	24h
IFNγ	CTL03.1	DCMU	100	68.69	200901	24h
IFNγ	CTL03.1	DCMU	10	81.73	200901	24h
IFNγ	CTL03.1	DMSO	250	137.17	200901	24h
IFNγ	CTL03.1	DMSO	100	144.78	200901	24h
IFNγ	CTL03.1	DMSO	10	91.52	200901	24h
IFNγ	CTL03.1	DCMU	250	64.34	200901	24h
IFNγ	CTL03.1	DCMU	100	79.56	200901	24h
IFNγ	CTL03.1	DCMU	10	90.43	200901	24h
IFNγ	CTL03.1	DMSO	250	164.34	200901	24h
IFNγ	CTL03.1	DMSO	100	106.73	200901	24h
IFNγ	CTL03.1	DMSO	10	94.78	200901	24h
IFNγ	CTL03.1	DCMU	250	68.69	200901	24h
IFNγ	CTL03.1	DCMU	100	70.86	200901	24h
IFNγ	CTL03.1	DCMU	10	92.60	200901	24h
IFNγ	CTL03.1	DMSO	250	160	200901	24h
IFNγ	CTL03.1	DMSO	100	108.91	200901	24h
IFNγ	CTL03.1	DMSO	10	89.34	200901	24h
IFNγ	CTL03.1	DCMU	250	66.52	200901	24h
IFNγ	CTL03.1	DCMU	100	68.69	200901	24h
IFNγ	CTL03.1	DCMU	10	93.69	200901	24h
IFNγ	CTL03.1	DMSO	250	996.95	200901	D5
IFNγ	CTL03.1	DMSO	100	801.30	200901	D5
IFNγ	CTL03.1	DMSO	10	628.47	200901	D5
IFNγ	CTL03.1	DCMU	250	430.65	200901	D5
IFNγ	CTL03.1	DCMU	100	627.39	200901	D5
IFNγ	CTL03.1	DCMU	10	632.82	200901	D5
IFNγ	CTL03.1	DMSO	250	1018.69	200901	D5
IFNγ	CTL03.1	DMSO	100	783.91	200901	D5
IFNγ	CTL03.1	DMSO	10	586.08	200901	D5
IFNγ	CTL03.1	DCMU	250	451.30	200901	D5
IFNγ	CTL03.1	DCMU	100	592.60	200901	D5
IFNγ	CTL03.1	DCMU	10	642.60	200901	D5
IFNγ	CTL03.1	DMSO	250	646.95	200901	D5
IFNγ	CTL03.1	DMSO	100	674.13	200901	D5
IFNγ	CTL03.1	DMSO	10	874.13	200901	D5
IFNγ	CTL03.1	DCMU	250	457.82	200901	D5
IFNγ	CTL03.1	DCMU	100	515.43	200901	D5
IFNγ	CTL03.1	DCMU	10	476.30	200901	D5
IFNγ	CTL03.1	DMSO	250	641.52	200901	D5
IFNγ	CTL03.1	DMSO	100	653.47	200901	D5
IFNγ	CTL03.1	DMSO	10	871.95	200901	D5
IFNγ	CTL03.1	DCMU	250	455.65	200901	D5

IFNγ	CTL03.1	DCMU	100	507.82	200901	D5
IFNγ	CTL03.1	DCMU	10	493.69	200901	D5
IFNγ	CTL03.1	DMSO	250	779.66	200915	D5
IFNγ	CTL03.1	DMSO	100	869.66	200915	D5
IFNγ	CTL03.1	DMSO	10	956.33	200915	D5
IFNγ	CTL03.1	DCMU	250	489.66	200915	D5
IFNγ	CTL03.1	DCMU	100	493	200915	D5
IFNγ	CTL03.1	DCMU	10	916.33	200915	D5
IFNγ	CTL03.1	DMSO	250	769.66	200915	D5
IFNγ	CTL03.1	DMSO	100	853	200915	D5
IFNγ	CTL03.1	DMSO	10	916.33	200915	D5
IFNγ	CTL03.1	DCMU	250	476.33	200915	D5
IFNγ	CTL03.1	DCMU	100	476.33	200915	D5
IFNγ	CTL03.1	DCMU	10	873	200915	D5
IFNγ	CTL03.1	DMSO	250	1048.69	200629	24h
IFNγ	CTL03.1	DMSO	100	974.78	200629	24h
IFNγ	CTL03.1	DMSO	10	1031.30	200629	24h
IFNγ	CTL03.1	DCMU	250	861.73	200629	24h
IFNγ	CTL03.1	DCMU	100	935.65	200629	24h
IFNγ	CTL03.1	DCMU	10	1126.95	200629	24h
IFNγ	CTL03.1	DMSO	250	1018.26	200629	24h
IFNγ	CTL03.1	DMSO	100	1000.86	200629	24h
IFNγ	CTL03.1	DMSO	10	1040	200629	24h
IFNγ	CTL03.1	DCMU	250	870.43	200629	24h
IFNγ	CTL03.1	DCMU	100	931.30	200629	24h
IFNγ	CTL03.1	DCMU	10	1222.60	200629	24h
IFNγ	CTL03.1	DMSO	250	1013.91	200629	24h
IFNγ	CTL03.1	DMSO	100	970.43	200629	24h
IFNγ	CTL03.1	DMSO	10	1105.21	200629	24h
IFNγ	CTL03.1	DCMU	250	848.69	200629	24h
IFNγ	CTL03.1	DCMU	100	948.69	200629	24h
IFNγ	CTL03.1	DCMU	10	1070.43	200629	24h
IFNγ	CTL03.1	DMSO	250	1000.86	200629	24h
IFNγ	CTL03.1	DMSO	100	961.73	200629	24h
IFNγ	CTL03.1	DMSO	10	1070.43	200629	24h
IFNγ	CTL03.1	DCMU	250	848.69	200629	24h
IFNγ	CTL03.1	DCMU	100	944.34	200629	24h
IFNγ	CTL03.1	DCMU	10	1057.39	200629	24h
IFNγ	CTL03.1	DMSO	250	81.21	200804	24h
IFNγ	CTL03.1	DMSO	100	84.24	200804	24h
IFNγ	CTL03.1	DMSO	10	84.24	200804	24h
IFNγ	CTL03.1	DCMU	250	53.93	200804	24h
IFNγ	CTL03.1	DCMU	100	66.06	200804	24h
IFNγ	CTL03.1	DCMU	10	72.12	200804	24h
IFNγ	CTL03.1	DMSO	250	84.24	200804	24h
IFNγ	CTL03.1	DMSO	100	75.15	200804	24h
IFNγ	CTL03.1	DMSO	10	84.24	200804	24h
IFNγ	CTL03.1	DCMU	250	35.75	200804	24h
IFNγ	CTL03.1	DCMU	100	60	200804	24h
IFNγ	CTL03.1	DCMU	10	69.09	200804	24h
IFNγ	CTL03.1	DMSO	250	78.18	200804	24h
IFNγ	CTL03.1	DMSO	100	81.21	200804	24h

IFNγ	CTL03.1	DMSO	10	87.27	200804	24h
IFNγ	CTL03.1	DCMU	250	38.78	200804	24h
IFNγ	CTL03.1	DCMU	100	66.06	200804	24h
IFNγ	CTL03.1	DCMU	10	72.12	200804	24h
IFNγ	CTL03.1	DMSO	250	420.60	200804	D5
IFNγ	CTL03.1	DMSO	100	378.18	200804	D5
IFNγ	CTL03.1	DMSO	10	384.24	200804	D5
IFNγ	CTL03.1	DCMU	250	360	200804	D5
IFNγ	CTL03.1	DCMU	100	384.24	200804	D5
IFNγ	CTL03.1	DCMU	10	569.09	200804	D5
IFNγ	CTL03.1	DMSO	250	438.78	200804	D5
IFNγ	CTL03.1	DMSO	100	393.33	200804	D5
IFNγ	CTL03.1	DMSO	10	390.30	200804	D5
IFNγ	CTL03.1	DCMU	250	366.06	200804	D5
IFNγ	CTL03.1	DCMU	100	390.30	200804	D5
IFNγ	CTL03.1	DCMU	10	584.24	200804	D5
IFNγ	CTL03.1	DMSO	250	423.63	200804	D5
IFNγ	CTL03.1	DMSO	100	402.42	200804	D5
IFNγ	CTL03.1	DMSO	10	381.21	200804	D5
IFNγ	CTL03.1	DCMU	250	366.06	200804	D5
IFNγ	CTL03.1	DCMU	100	372.12	200804	D5
IFNγ	CTL03.1	DCMU	10	566.06	200804	D5
GrB	CTL03.1	DMSO	250	18277.92	200901	24h
GrB	CTL03.1	DMSO	100	19784.41	200901	24h
GrB	CTL03.1	DMSO	10	16148.05	200901	24h
GrB	CTL03.1	DCMU	250	10122.07	200901	24h
GrB	CTL03.1	DCMU	100	12979.22	200901	24h
GrB	CTL03.1	DCMU	10	11368.83	200901	24h
GrB	CTL03.1	DMSO	250	16823.37	200901	24h
GrB	CTL03.1	DMSO	100	19810.38	200901	24h
GrB	CTL03.1	DMSO	10	15914.28	200901	24h
GrB	CTL03.1	DCMU	250	10277.92	200901	24h
GrB	CTL03.1	DCMU	100	12537.66	200901	24h
GrB	CTL03.1	DCMU	10	12849.35	200901	24h
GrB	CTL03.1	DMSO	250	20148.05	200901	24h
GrB	CTL03.1	DMSO	100	16589.61	200901	24h
GrB	CTL03.1	DMSO	10	16693.50	200901	24h
GrB	CTL03.1	DCMU	250	8563.63	200901	24h
GrB	CTL03.1	DCMU	100	12070.12	200901	24h
GrB	CTL03.1	DCMU	10	12070.12	200901	24h
GrB	CTL03.1	DMSO	250	18771.42	200901	24h
GrB	CTL03.1	DMSO	100	14303.89	200901	24h
GrB	CTL03.1	DMSO	10	14641.55	200901	24h
GrB	CTL03.1	DCMU	250	7810.38	200901	24h
GrB	CTL03.1	DCMU	100	12771.42	200901	24h
GrB	CTL03.1	DCMU	10	14797.40	200901	24h
GrB	CTL03.1	DMSO	250	32174.02	200901	D5
GrB	CTL03.1	DMSO	100	24018.18	200901	D5
GrB	CTL03.1	DMSO	10	18693.50	200901	D5
GrB	CTL03.1	DCMU	250	11836.36	200901	D5
GrB	CTL03.1	DCMU	100	19914.28	200901	D5
GrB	CTL03.1	DCMU	10	16200	200901	D5

GrB	CTL03.1	DMSO	250	31576.62	200901	D5
GrB	CTL03.1	DMSO	100	24979.22	200901	D5
GrB	CTL03.1	DMSO	10	19654.54	200901	D5
GrB	CTL03.1	DCMU	250	11862.33	200901	D5
GrB	CTL03.1	DCMU	100	19446.75	200901	D5
GrB	CTL03.1	DCMU	10	17706.49	200901	D5
GrB	CTL03.1	DMSO	250	12953.24	200901	D5
GrB	CTL03.1	DMSO	100	5862.33	200901	D5
GrB	CTL03.1	DMSO	10	6849.35	200901	D5
GrB	CTL03.1	DCMU	250	3992.20	200901	D5
GrB	CTL03.1	DCMU	100	5394.80	200901	D5
GrB	CTL03.1	DCMU	10	5602.59	200901	D5
GrB	CTL03.1	DMSO	250	12200	200901	D5
GrB	CTL03.1	DMSO	100	6303.89	200901	D5
GrB	CTL03.1	DMSO	10	7290.90	200901	D5
GrB	CTL03.1	DCMU	250	4303.89	200901	D5
GrB	CTL03.1	DCMU	100	5212.98	200901	D5
GrB	CTL03.1	DCMU	10	6018.18	200901	D5
GrB	CTL03.1	DMSO	250	117008.33	200916	D5
GrB	CTL03.1	DMSO	100	169175	200916	D5
GrB	CTL03.1	DMSO	10	221508.33	200916	D5
GrB	CTL03.1	DCMU	250	47591.66	200916	D5
GrB	CTL03.1	DCMU	100	109758.33	200916	D5
GrB	CTL03.1	DCMU	10	196258.33	200916	D5
GrB	CTL03.1	DMSO	250	140841.66	200916	D5
GrB	CTL03.1	DMSO	100	168091.66	200916	D5
GrB	CTL03.1	DMSO	10	201508.33	200916	D5
GrB	CTL03.1	DCMU	250	50425	200916	D5
GrB	CTL03.1	DCMU	100	118091.66	200916	D5
GrB	CTL03.1	DCMU	10	199675	200916	D5
GrB	CTL03.1	DMSO	250	21679.36	201105	24h
GrB	CTL03.1	DMSO	100	20028.57	201105	24h
GrB	CTL03.1	DMSO	10	20663.49	201105	24h
GrB	CTL03.1	DCMU	250	4409.52	201105	24h
GrB	CTL03.1	DCMU	100	18219.04	201105	24h
GrB	CTL03.1	DCMU	10	19488.88	201105	24h
GrB	CTL03.1	DMSO	250	21965.07	201105	24h
GrB	CTL03.1	DMSO	100	20219.04	201105	24h
GrB	CTL03.1	DMSO	10	20092.06	201105	24h
GrB	CTL03.1	DCMU	250	4536.50	201105	24h
GrB	CTL03.1	DCMU	100	18758.73	201105	24h
GrB	CTL03.1	DCMU	10	22155.55	201105	24h
GrB	CTL03.1	DMSO	250	19488.88	201105	D5
GrB	CTL03.1	DMSO	100	20853.96	201105	D5
GrB	CTL03.1	DMSO	10	27647.61	201105	D5
GrB	CTL03.1	DCMU	250	2123.80	201105	D5
GrB	CTL03.1	DCMU	100	14123.80	201105	D5
GrB	CTL03.1	DCMU	10	24219.04	201105	D5
GrB	CTL03.1	DMSO	250	18790.47	201105	D5
GrB	CTL03.1	DMSO	100	21107.93	201105	D5
GrB	CTL03.1	DMSO	10	27584.12	201105	D5
GrB	CTL03.1	DCMU	250	2250.79	201105	D5

GrB	CTL03.1	DCMU	100	14790.47	201105	D5
GrB	CTL03.1	DCMU	10	23552.38	201105	D5
GrB	CTL03.1	DMSO	250	22124.52	200629	24h
GrB	CTL03.1	DMSO	100	29822.64	200629	24h
GrB	CTL03.1	DMSO	10	28539.62	200629	24h
GrB	CTL03.1	DCMU	250	12615.09	200629	24h
GrB	CTL03.1	DCMU	100	35709.43	200629	24h
GrB	CTL03.1	DCMU	10	32652.83	200629	24h
GrB	CTL03.1	DMSO	250	30954.71	200629	24h
GrB	CTL03.1	DMSO	100	32615.09	200629	24h
GrB	CTL03.1	DMSO	10	38652.83	200629	24h
GrB	CTL03.1	DCMU	250	15369.81	200629	24h
GrB	CTL03.1	DCMU	100	44841.50	200629	24h
GrB	CTL03.1	DCMU	10	44916.98	200629	24h
GrB	CTL03.1	DMSO	250	20916.98	200629	24h
GrB	CTL03.1	DMSO	100	30879.24	200629	24h
GrB	CTL03.1	DMSO	10	29520.75	200629	24h
GrB	CTL03.1	DCMU	250	15143.39	200629	24h
GrB	CTL03.1	DCMU	100	41067.92	200629	24h
GrB	CTL03.1	DCMU	10	47143.39	200629	24h
GrB	CTL03.1	DMSO	250	32388.67	200629	24h
GrB	CTL03.1	DMSO	100	32615.09	200629	24h
GrB	CTL03.1	DMSO	10	43483.01	200629	24h
GrB	CTL03.1	DCMU	250	18388.67	200629	24h
GrB	CTL03.1	DCMU	100	45483.01	200629	24h
GrB	CTL03.1	DCMU	10	58728.30	200629	24h
GrB	CTL03.1	DMSO	250	16336.17	200803	24h
GrB	CTL03.1	DMSO	100	20974.46	200803	24h
GrB	CTL03.1	DMSO	10	28336.17	200803	24h
GrB	CTL03.1	DCMU	250	5314.89	200803	24h
GrB	CTL03.1	DCMU	100	18846.80	200803	24h
GrB	CTL03.1	DCMU	10	19272.34	200803	24h
GrB	CTL03.1	DMSO	250	17314.89	200803	24h
GrB	CTL03.1	DMSO	100	20889.36	200803	24h
GrB	CTL03.1	DMSO	10	29910.63	200803	24h
GrB	CTL03.1	DCMU	250	5400	200803	24h
GrB	CTL03.1	DCMU	100	20889.36	200803	24h
GrB	CTL03.1	DCMU	10	22889.36	200803	24h
GrB	CTL03.1	DMSO	250	21400	200803	24h
GrB	CTL03.1	DMSO	100	27910.63	200803	24h
GrB	CTL03.1	DMSO	10	34165.95	200803	24h
GrB	CTL03.1	DCMU	250	5953.19	200803	24h
GrB	CTL03.1	DCMU	100	32548.93	200803	24h
GrB	CTL03.1	DCMU	10	47485.10	200803	24h
GrB	CTL03.1	DMSO	250	24804.25	200803	24h
GrB	CTL03.1	DMSO	100	28336.17	200803	24h
GrB	CTL03.1	DMSO	10	30548.93	200803	24h
GrB	CTL03.1	DCMU	250	6378.72	200803	24h
GrB	CTL03.1	DCMU	100	31697.87	200803	24h
GrB	CTL03.1	DCMU	10	47910.63	200803	24h
GrB	CTL03.1	DMSO	250	3272.34	200803	D5
GrB	CTL03.1	DMSO	100	2548.93	200803	D5

GrB	CTL03.1	DMSO	10	2165.95	200803	D5
GrB	CTL03.1	DCMU	250	3017.02	200803	D5
GrB	CTL03.1	DCMU	100	2634.04	200803	D5
GrB	CTL03.1	DCMU	10	4463.82	200803	D5
GrB	CTL03.1	DMSO	250	2889.36	200803	D5
GrB	CTL03.1	DMSO	100	2463.82	200803	D5
GrB	CTL03.1	DMSO	10	2208.51	200803	D5
GrB	CTL03.1	DCMU	250	2676.59	200803	D5
GrB	CTL03.1	DCMU	100	2506.38	200803	D5
GrB	CTL03.1	DCMU	10	4548.93	200803	D5
GrB	CTL03.1	DMSO	250	1527.65	200803	D5
GrB	CTL03.1	DMSO	100	1782.97	200803	D5
GrB	CTL03.1	DMSO	10	719.14	200803	D5
GrB	CTL03.1	DCMU	250	2889.36	200803	D5
GrB	CTL03.1	DCMU	100	2251.06	200803	D5
GrB	CTL03.1	DCMU	10	2165.95	200803	D5
GrB	CTL03.1	DMSO	250	1655.31	200803	D5
GrB	CTL03.1	DMSO	100	1527.65	200803	D5
GrB	CTL03.1	DMSO	10	676.59	200803	D5
GrB	CTL03.1	DCMU	250	2378.72	200803	D5
GrB	CTL03.1	DCMU	100	2038.29	200803	D5
GrB	CTL03.1	DCMU	10	1655.31	200803	D5
TNFα	CTL03.1	DMSO	250	40.18	200903	D5
TNFα	CTL03.1	DMSO	100	40.55	200903	D5
TNFα	CTL03.1	DMSO	10	49.88	200903	D5
TNFα	CTL03.1	DCMU	250	34.21	200903	D5
TNFα	CTL03.1	DCMU	100	46.90	200903	D5
TNFα	CTL03.1	DCMU	10	27.12	200903	D5
TNFα	CTL03.1	DMSO	250	85.70	200903	D5
TNFα	CTL03.1	DMSO	100	70.78	200903	D5
TNFα	CTL03.1	DMSO	10	33.84	200903	D5
TNFα	CTL03.1	DCMU	250	52.5	200903	D5
TNFα	CTL03.1	DCMU	100	61.45	200903	D5
TNFα	CTL03.1	DCMU	10	59.21	200903	D5
TNFα	CTL03.1	DMSO	250	70.41	200903	D5
TNFα	CTL03.1	DMSO	100	68.17	200903	D5
TNFα	CTL03.1	DMSO	10	35.70	200903	D5
TNFα	CTL03.1	DCMU	250	42.42	200903	D5
TNFα	CTL03.1	DCMU	100	45.41	200903	D5
TNFα	CTL03.1	DCMU	10	33.09	200903	D5
TNFα	CTL03.1	DMSO	250	24.14	200903	D5
TNFα	CTL03.1	DMSO	100	25.26	200903	D5
TNFα	CTL03.1	DMSO	10	17.79	200903	D5
TNFα	CTL03.1	DCMU	250	25.63	200903	D5
TNFα	CTL03.1	DCMU	100	28.99	200903	D5
TNFα	CTL03.1	DCMU	10	18.91	200903	D5
TNFα	CTL03.1	DMSO	250	20.41	200903	D5
TNFα	CTL03.1	DMSO	100	14.44	200903	D5
TNFα	CTL03.1	DMSO	10	15.93	200903	D5
TNFα	CTL03.1	DCMU	250	20.78	200903	D5
TNFα	CTL03.1	DCMU	100	20.78	200903	D5
TNFα	CTL03.1	DCMU	10	18.54	200903	D5

TNFα	CTL03.1	DMSO	250	23.39	200903	D5
TNFα	CTL03.1	DMSO	100	15.55	200903	D5
TNFα	CTL03.1	DMSO	10	15.93	200903	D5
TNFα	CTL03.1	DCMU	250	27.12	200903	D5
TNFα	CTL03.1	DCMU	100	22.64	200903	D5
TNFα	CTL03.1	DCMU	10	16.67	200903	D5
TNFα	CTL03.1	DMSO	250	23.76	200903	D5
TNFα	CTL03.1	DMSO	100	21.52	200903	D5
TNFα	CTL03.1	DMSO	10	20.78	200903	D5
TNFα	CTL03.1	DCMU	250	26.75	200903	D5
TNFα	CTL03.1	DCMU	100	21.90	200903	D5
TNFα	CTL03.1	DCMU	10	19.66	200903	D5
TNFα	CTL03.1	DMSO	250	24.51	200903	D5
TNFα	CTL03.1	DMSO	100	18.91	200903	D5
TNFα	CTL03.1	DMSO	10	18.17	200903	D5
TNFα	CTL03.1	DCMU	250	21.15	200903	D5
TNFα	CTL03.1	DCMU	100	20.41	200903	D5
TNFα	CTL03.1	DCMU	10	18.17	200903	D5
TNFα	CTL03.1	DMSO	250	25.26	200903	D5
TNFα	CTL03.1	DMSO	100	21.52	200903	D5
TNFα	CTL03.1	DMSO	10	17.05	200903	D5
TNFα	CTL03.1	DCMU	250	20.41	200903	D5
TNFα	CTL03.1	DCMU	100	19.66	200903	D5
TNFα	CTL03.1	DCMU	10	16.30	200903	D5
TNFα	CTL03.1	DMSO	250	118.37	200915	D5
TNFα	CTL03.1	DMSO	100	125.13	200915	D5
TNFα	CTL03.1	DMSO	10	183.24	200915	D5
TNFα	CTL03.1	DCMU	250	87.29	200915	D5
TNFα	CTL03.1	DCMU	100	61.62	200915	D5
TNFα	CTL03.1	DCMU	10	199.45	200915	D5
TNFα	CTL03.1	DMSO	250	127.83	200915	D5
TNFα	CTL03.1	DMSO	100	133.24	200915	D5
TNFα	CTL03.1	DMSO	10	198.10	200915	D5
TNFα	CTL03.1	DCMU	250	98.10	200915	D5
TNFα	CTL03.1	DCMU	100	85.94	200915	D5
TNFα	CTL03.1	DCMU	10	214.32	200915	D5
TNFα	CTL03.1	DMSO	250	517.6	201105	24h
TNFα	CTL03.1	DMSO	100	529.6	201105	24h
TNFα	CTL03.1	DMSO	10	578.4	201105	24h
TNFα	CTL03.1	DCMU	250	220	201105	24h
TNFα	CTL03.1	DCMU	100	484	201105	24h
TNFα	CTL03.1	DCMU	10	619.2	201105	24h
TNFα	CTL03.1	DMSO	250	551.2	201105	24h
TNFα	CTL03.1	DMSO	100	517.6	201105	24h
TNFα	CTL03.1	DMSO	10	567.2	201105	24h
TNFα	CTL03.1	DCMU	250	220.8	201105	24h
TNFα	CTL03.1	DCMU	100	469.6	201105	24h
TNFα	CTL03.1	DCMU	10	612	201105	24h
TNFα	CTL03.1	DMSO	250	549.6	201105	D5
TNFα	CTL03.1	DMSO	100	573.6	201105	D5
TNFα	CTL03.1	DMSO	10	528.8	201105	D5
TNFα	CTL03.1	DCMU	250	160.8	201105	D5

TNFα	CTL03.1	DCMU	100	296	201105	D5
TNFα	CTL03.1	DCMU	10	680.8	201105	D5
TNFα	CTL03.1	DMSO	250	559.2	201105	D5
TNFα	CTL03.1	DMSO	100	600.8	201105	D5
TNFα	CTL03.1	DMSO	10	580	201105	D5
TNFα	CTL03.1	DCMU	250	177.6	201105	D5
TNFα	CTL03.1	DCMU	100	279.2	201105	D5
TNFα	CTL03.1	DCMU	10	628	201105	D5
TNFα	CTL03.1	DMSO	250	1614.16	200626	24h
TNFα	CTL03.1	DMSO	100	1626.66	200626	24h
TNFα	CTL03.1	DMSO	10	1664.16	200626	24h
TNFα	CTL03.1	DCMU	250	968.33	200626	24h
TNFα	CTL03.1	DCMU	100	1239.16	200626	24h
TNFα	CTL03.1	DCMU	10	1435	200626	24h
TNFα	CTL03.1	DMSO	250	1593.33	200626	24h
TNFα	CTL03.1	DMSO	100	1614.16	200626	24h
TNFα	CTL03.1	DMSO	10	1589.16	200626	24h
TNFα	CTL03.1	DCMU	250	930.83	200626	24h
TNFα	CTL03.1	DCMU	100	1226.66	200626	24h
TNFα	CTL03.1	DCMU	10	1380.83	200626	24h
TNFα	CTL03.1	DMSO	250	1568.33	200626	24h
TNFα	CTL03.1	DMSO	100	1597.5	200626	24h
TNFα	CTL03.1	DMSO	10	1693.33	200626	24h
TNFα	CTL03.1	DCMU	250	876.66	200626	24h
TNFα	CTL03.1	DCMU	100	1222.5	200626	24h
TNFα	CTL03.1	DCMU	10	1685	200626	24h
TNFα	CTL03.1	DMSO	250	1547.5	200626	24h
TNFα	CTL03.1	DMSO	100	1647.5	200626	24h
TNFα	CTL03.1	DMSO	10	1668.33	200626	24h
TNFα	CTL03.1	DCMU	250	943.33	200626	24h
TNFα	CTL03.1	DCMU	100	1289.16	200626	24h
TNFα	CTL03.1	DCMU	10	1685	200626	24h
TNFα	CTL03.1	DMSO	250	394.77	200804	24h
TNFα	CTL03.1	DMSO	100	401.59	200804	24h
TNFα	CTL03.1	DMSO	10	376.59	200804	24h
TNFα	CTL03.1	DCMU	250	299.31	200804	24h
TNFα	CTL03.1	DCMU	100	301.59	200804	24h
TNFα	CTL03.1	DCMU	10	360.68	200804	24h
TNFα	CTL03.1	DMSO	250	422.04	200804	24h
TNFα	CTL03.1	DMSO	100	419.77	200804	24h
TNFα	CTL03.1	DMSO	10	390.22	200804	24h
TNFα	CTL03.1	DCMU	250	328.86	200804	24h
TNFα	CTL03.1	DCMU	100	337.95	200804	24h
TNFα	CTL03.1	DCMU	10	369.77	200804	24h
TNFα	CTL03.1	DMSO	250	472.04	200804	24h
TNFα	CTL03.1	DMSO	100	412.95	200804	24h
TNFα	CTL03.1	DMSO	10	419.77	200804	24h
TNFα	CTL03.1	DCMU	250	376.59	200804	24h
TNFα	CTL03.1	DCMU	100	419.77	200804	24h
TNFα	CTL03.1	DCMU	10	453.86	200804	24h
TNFα	CTL03.1	DMSO	250	472.04	200804	24h
TNFα	CTL03.1	DMSO	100	419.77	200804	24h

TNFα	CTL03.1	DMSO	10	419.77	200804	24h
TNFα	CTL03.1	DCMU	250	387.95	200804	24h
TNFα	CTL03.1	DCMU	100	428.86	200804	24h
TNFα	CTL03.1	DCMU	10	451.59	200804	24h
IL2	CTL03.1	DMSO	250	2231.25	200626	24h
IL2	CTL03.1	DMSO	100	2371.87	200626	24h
IL2	CTL03.1	DMSO	10	2200	200626	24h
IL2	CTL03.1	DCMU	250	2309.37	200626	24h
IL2	CTL03.1	DCMU	100	2176.56	200626	24h
IL2	CTL03.1	DCMU	10	2168.75	200626	24h
IL2	CTL03.1	DMSO	250	2270.31	200626	24h
IL2	CTL03.1	DMSO	100	2434.37	200626	24h
IL2	CTL03.1	DMSO	10	2192.18	200626	24h
IL2	CTL03.1	DCMU	250	2325	200626	24h
IL2	CTL03.1	DCMU	100	2168.75	200626	24h
IL2	CTL03.1	DCMU	10	2270.31	200626	24h
IL2	CTL03.1	DMSO	250	2403.12	200626	24h
IL2	CTL03.1	DMSO	100	2160.93	200626	24h
IL2	CTL03.1	DMSO	10	2098.43	200626	24h
IL2	CTL03.1	DCMU	250	2098.43	200626	24h
IL2	CTL03.1	DCMU	100	2207.81	200626	24h
IL2	CTL03.1	DCMU	10	2160.93	200626	24h
IL2	CTL03.1	DMSO	250	2410.93	200626	24h
IL2	CTL03.1	DMSO	100	2160.93	200626	24h
IL2	CTL03.1	DMSO	10	2137.5	200626	24h
IL2	CTL03.1	DCMU	250	2192.18	200626	24h
IL2	CTL03.1	DCMU	100	2239.06	200626	24h
IL2	CTL03.1	DCMU	10	2114.06	200626	24h
IL2	CTL03.1	DMSO	250	6350	200805	D5
IL2	CTL03.1	DMSO	100	6520.73	200805	D5
IL2	CTL03.1	DMSO	10	6532.92	200805	D5
IL2	CTL03.1	DCMU	250	6484.14	200805	D5
IL2	CTL03.1	DCMU	100	6057.31	200805	D5
IL2	CTL03.1	DCMU	10	5789.02	200805	D5
IL2	CTL03.1	DMSO	250	6715.85	200805	D5
IL2	CTL03.1	DMSO	100	6679.26	200805	D5
IL2	CTL03.1	DMSO	10	6374.39	200805	D5
IL2	CTL03.1	DCMU	250	6545.12	200805	D5
IL2	CTL03.1	DCMU	100	6228.04	200805	D5
IL2	CTL03.1	DCMU	10	6069.51	200805	D5
IL2	CTL03.1	DMSO	250	7142.68	200805	D5
IL2	CTL03.1	DMSO	100	7057.31	200805	D5
IL2	CTL03.1	DMSO	10	7459.75	200805	D5
IL2	CTL03.1	DCMU	250	7423.17	200805	D5
IL2	CTL03.1	DCMU	100	7203.65	200805	D5
IL2	CTL03.1	DCMU	10	7045.12	200805	D5
IL2	CTL03.1	DMSO	250	7203.65	200805	D5
IL2	CTL03.1	DMSO	100	7142.68	200805	D5
IL2	CTL03.1	DMSO	10	7642.68	200805	D5
IL2	CTL03.1	DCMU	250	7691.46	200805	D5
IL2	CTL03.1	DCMU	100	7581.70	200805	D5
IL2	CTL03.1	DCMU	10	7630.48	200805	D5

IFNγ	N5.14	DMSO	250	1349.66	200915	D5
IFNγ	N5.14	DMSO	100	1653	200915	D5
IFNγ	N5.14	DMSO	10	1596.33	200915	D5
IFNγ	N5.14	DCMU	250	1313	200915	D5
IFNγ	N5.14	DCMU	100	1503	200915	D5
IFNγ	N5.14	DCMU	10	1626.33	200915	D5
IFNγ	N5.14	DMSO	250	1333	200915	D5
IFNγ	N5.14	DMSO	100	1719.66	200915	D5
IFNγ	N5.14	DMSO	10	1649.66	200915	D5
IFNγ	N5.14	DCMU	250	1346.33	200915	D5
IFNγ	N5.14	DCMU	100	1559.66	200915	D5
IFNγ	N5.14	DCMU	10	1593	200915	D5
IFNγ	N5.14	DMSO	250	212.93	201127	24h
IFNγ	N5.14	DMSO	100	169.18	201127	24h
IFNγ	N5.14	DMSO	10	154.81	201127	24h
IFNγ	N5.14	DCMU	250	70.43	201127	24h
IFNγ	N5.14	DCMU	100	137.31	201127	24h
IFNγ	N5.14	DCMU	10	149.81	201127	24h
IFNγ	N5.14	DMSO	250	195.43	201127	24h
IFNγ	N5.14	DMSO	100	152.31	201127	24h
IFNγ	N5.14	DMSO	10	141.68	201127	24h
IFNγ	N5.14	DCMU	250	66.68	201127	24h
IFNγ	N5.14	DCMU	100	125.43	201127	24h
IFNγ	N5.14	DCMU	10	147.93	201127	24h
IFNγ	N5.14	DMSO	250	171.68	201127	24h
IFNγ	N5.14	DMSO	100	163.56	201127	24h
IFNγ	N5.14	DMSO	10	137.93	201127	24h
IFNγ	N5.14	DCMU	250	51.68	201127	24h
IFNγ	N5.14	DCMU	100	122.31	201127	24h
IFNγ	N5.14	DCMU	10	157.31	201127	24h
IFNγ	N5.14	DMSO	250	167.31	201127	24h
IFNγ	N5.14	DMSO	100	154.81	201127	24h
IFNγ	N5.14	DMSO	10	137.31	201127	24h
IFNγ	N5.14	DCMU	250	52.93	201127	24h
IFNγ	N5.14	DCMU	100	122.93	201127	24h
IFNγ	N5.14	DCMU	10	153.56	201127	24h
IFNγ	N5.14	DMSO	250	135.87	201201	D5
IFNγ	N5.14	DMSO	100	101.67	201201	D5
IFNγ	N5.14	DMSO	10	106.19	201201	D5
IFNγ	N5.14	DCMU	250	108.12	201201	D5
IFNγ	N5.14	DCMU	100	104.90	201201	D5
IFNγ	N5.14	DCMU	10	127.48	201201	D5
IFNγ	N5.14	DMSO	250	126.19	201201	D5
IFNγ	N5.14	DMSO	100	81.03	201201	D5
IFNγ	N5.14	DMSO	10	91.35	201201	D5
IFNγ	N5.14	DCMU	250	86.83	201201	D5
IFNγ	N5.14	DCMU	100	97.80	201201	D5
IFNγ	N5.14	DCMU	10	114.58	201201	D5
IFNγ	N5.14	DMSO	250	98.45	201201	D5
IFNγ	N5.14	DMSO	100	46.83	201201	D5
IFNγ	N5.14	DMSO	10	77.16	201201	D5
IFNγ	N5.14	DCMU	250	82.32	201201	D5

IFNγ	N5.14	DCMU	100	86.83	201201	D5
IFNγ	N5.14	DCMU	10	99.09	201201	D5
IFNγ	N5.14	DMSO	250	105.54	201201	D5
IFNγ	N5.14	DMSO	100	66.19	201201	D5
IFNγ	N5.14	DMSO	10	81.67	201201	D5
IFNγ	N5.14	DCMU	250	85.54	201201	D5
IFNγ	N5.14	DCMU	100	92	201201	D5
IFNγ	N5.14	DCMU	10	104.90	201201	D5
IFNγ	N5.14	DMSO	250	1170.43	200629	24h
IFNγ	N5.14	DMSO	100	1183.47	200629	24h
IFNγ	N5.14	DMSO	10	1205.21	200629	24h
IFNγ	N5.14	DCMU	250	1018.26	200629	24h
IFNγ	N5.14	DCMU	100	1148.69	200629	24h
IFNγ	N5.14	DCMU	10	1348.69	200629	24h
IFNγ	N5.14	DMSO	250	1157.39	200629	24h
IFNγ	N5.14	DMSO	100	1161.73	200629	24h
IFNγ	N5.14	DMSO	10	1248.69	200629	24h
IFNγ	N5.14	DCMU	250	953.04	200629	24h
IFNγ	N5.14	DCMU	100	1096.52	200629	24h
IFNγ	N5.14	DCMU	10	1200.86	200629	24h
IFNγ	N5.14	DMSO	250	1140	200629	24h
IFNγ	N5.14	DMSO	100	1022.60	200629	24h
IFNγ	N5.14	DMSO	10	1044.34	200629	24h
IFNγ	N5.14	DCMU	250	866.08	200629	24h
IFNγ	N5.14	DCMU	100	1009.56	200629	24h
IFNγ	N5.14	DCMU	10	1140	200629	24h
IFNγ	N5.14	DMSO	250	1161.73	200629	24h
IFNγ	N5.14	DMSO	100	931.30	200629	24h
IFNγ	N5.14	DMSO	10	940	200629	24h
IFNγ	N5.14	DCMU	250	813.91	200629	24h
IFNγ	N5.14	DCMU	100	948.69	200629	24h
IFNγ	N5.14	DCMU	10	983.47	200629	24h
IFNγ	N5.14	DMSO	250	641.81	200804	D5
IFNγ	N5.14	DMSO	100	563.03	200804	D5
IFNγ	N5.14	DMSO	10	560	200804	D5
IFNγ	N5.14	DCMU	250	556.96	200804	D5
IFNγ	N5.14	DCMU	100	535.75	200804	D5
IFNγ	N5.14	DCMU	10	514.54	200804	D5
IFNγ	N5.14	DMSO	250	635.75	200804	D5
IFNγ	N5.14	DMSO	100	556.96	200804	D5
IFNγ	N5.14	DMSO	10	553.93	200804	D5
IFNγ	N5.14	DCMU	250	508.48	200804	D5
IFNγ	N5.14	DCMU	100	529.69	200804	D5
IFNγ	N5.14	DCMU	10	520.60	200804	D5
IFNγ	N5.14	DMSO	250	632.72	200804	D5
IFNγ	N5.14	DMSO	100	538.78	200804	D5
IFNγ	N5.14	DMSO	10	550.90	200804	D5
IFNγ	N5.14	DCMU	250	517.57	200804	D5
IFNγ	N5.14	DCMU	100	541.81	200804	D5
IFNγ	N5.14	DCMU	10	505.45	200804	D5
IFNγ	N5.14	DMSO	250	135.87	201201	D5
IFNγ	N5.14	DMSO	100	101.67	201201	D5

IFNγ	N5.14	DMSO	10	106.19	201201	D5
IFNγ	N5.14	DCMU	250	108.12	201201	D5
IFNγ	N5.14	DCMU	100	104.90	201201	D5
IFNγ	N5.14	DCMU	10	127.48	201201	D5
IFNγ	N5.14	DMSO	250	126.19	201201	D5
IFNγ	N5.14	DMSO	100	81.03	201201	D5
IFNγ	N5.14	DMSO	10	91.35	201201	D5
IFNγ	N5.14	DCMU	250	86.83	201201	D5
IFNγ	N5.14	DCMU	100	97.80	201201	D5
IFNγ	N5.14	DCMU	10	114.58	201201	D5
IFNγ	N5.14	DMSO	250	98.45	201201	D5
IFNγ	N5.14	DMSO	100	46.83	201201	D5
IFNγ	N5.14	DMSO	10	77.16	201201	D5
IFNγ	N5.14	DCMU	250	82.32	201201	D5
IFNγ	N5.14	DCMU	100	86.83	201201	D5
IFNγ	N5.14	DCMU	10	99.09	201201	D5
IFNγ	N5.14	DMSO	250	105.54	201201	D5
IFNγ	N5.14	DMSO	100	66.19	201201	D5
IFNγ	N5.14	DMSO	10	81.67	201201	D5
IFNγ	N5.14	DCMU	250	85.54	201201	D5
IFNγ	N5.14	DCMU	100	92	201201	D5
IFNγ	N5.14	DCMU	10	104.90	201201	D5
TNFα	N5.14	DMSO	250	142.70	200915	D5
TNFα	N5.14	DMSO	100	187.29	200915	D5
TNFα	N5.14	DMSO	10	169.72	200915	D5
TNFα	N5.14	DCMU	250	126.48	200915	D5
TNFα	N5.14	DCMU	100	179.18	200915	D5
TNFα	N5.14	DCMU	10	184.59	200915	D5
TNFα	N5.14	DMSO	250	134.59	200915	D5
TNFα	N5.14	DMSO	100	179.18	200915	D5
TNFα	N5.14	DMSO	10	162.97	200915	D5
TNFα	N5.14	DCMU	250	127.83	200915	D5
TNFα	N5.14	DCMU	100	171.08	200915	D5
TNFα	N5.14	DCMU	10	184.59	200915	D5
TNFα	N5.14	DMSO	250	48.8	201105	24h
TNFα	N5.14	DMSO	100	124.8	201105	24h
TNFα	N5.14	DMSO	10	37.6	201105	24h
TNFα	N5.14	DCMU	250	52	201105	24h
TNFα	N5.14	DCMU	100	38.4	201105	24h
TNFα	N5.14	DCMU	10	40.8	201105	24h
TNFα	N5.14	DMSO	250	49.6	201105	24h
TNFα	N5.14	DMSO	100	122.4	201105	24h
TNFα	N5.14	DMSO	10	39.2	201105	24h
TNFα	N5.14	DCMU	250	51.2	201105	24h
TNFα	N5.14	DCMU	100	33.6	201105	24h
TNFα	N5.14	DCMU	10	39.2	201105	24h
TNFα	N5.14	DMSO	250	89.6	201105	D5
TNFα	N5.14	DMSO	100	61.6	201105	D5
TNFα	N5.14	DMSO	10	83.2	201105	D5
TNFα	N5.14	DCMU	250	144.8	201105	D5
TNFα	N5.14	DCMU	100	84	201105	D5
TNFα	N5.14	DCMU	10	75.2	201105	D5

TNFα	N5.14	DMSO	250	99.2	201105	D5
TNFα	N5.14	DMSO	100	64	201105	D5
TNFα	N5.14	DMSO	10	84	201105	D5
TNFα	N5.14	DCMU	250	149.6	201105	D5
TNFα	N5.14	DCMU	100	84.8	201105	D5
TNFα	N5.14	DCMU	10	72.8	201105	D5
TNFα	N5.14	DMSO	250	1780.83	200626	24h
TNFα	N5.14	DMSO	100	1860	200626	24h
TNFα	N5.14	DMSO	10	1743.33	200626	24h
TNFα	N5.14	DCMU	250	1030.83	200626	24h
TNFα	N5.14	DCMU	100	1397.5	200626	24h
TNFα	N5.14	DCMU	10	1764.16	200626	24h
TNFα	N5.14	DMSO	250	1776.66	200626	24h
TNFα	N5.14	DMSO	100	1939.16	200626	24h
TNFα	N5.14	DMSO	10	1893.33	200626	24h
TNFα	N5.14	DCMU	250	1068.33	200626	24h
TNFα	N5.14	DCMU	100	1530.83	200626	24h
TNFα	N5.14	DCMU	10	1805.83	200626	24h
TNFα	N5.14	DMSO	250	1822.5	200626	24h
TNFα	N5.14	DMSO	100	1797.5	200626	24h
TNFα	N5.14	DMSO	10	1655.83	200626	24h
TNFα	N5.14	DCMU	250	1060	200626	24h
TNFα	N5.14	DCMU	100	1655.83	200626	24h
TNFα	N5.14	DCMU	10	1835	200626	24h
TNFα	N5.14	DMSO	250	1726.66	200626	24h
TNFα	N5.14	DMSO	100	1814.16	200626	24h
TNFα	N5.14	DMSO	10	1764.16	200626	24h
TNFα	N5.14	DCMU	250	1101.66	200626	24h
TNFα	N5.14	DCMU	100	1597.5	200626	24h
TNFα	N5.14	DCMU	10	1893.33	200626	24h
TNFα	N5.14	DMSO	250	758.40	200804	24h
TNFα	N5.14	DMSO	100	790.22	200804	24h
TNFα	N5.14	DMSO	10	803.86	200804	24h
TNFα	N5.14	DCMU	250	481.13	200804	24h
TNFα	N5.14	DCMU	100	703.86	200804	24h
TNFα	N5.14	DCMU	10	812.95	200804	24h
TNFα	N5.14	DMSO	250	831.13	200804	24h
TNFα	N5.14	DMSO	100	790.22	200804	24h
TNFα	N5.14	DMSO	10	835.68	200804	24h
TNFα	N5.14	DCMU	250	483.40	200804	24h
TNFα	N5.14	DCMU	100	708.40	200804	24h
TNFα	N5.14	DCMU	10	847.04	200804	24h
TNFα	N5.14	DMSO	250	733.40	200804	24h
TNFα	N5.14	DMSO	100	878.86	200804	24h
TNFα	N5.14	DMSO	10	826.59	200804	24h
TNFα	N5.14	DCMU	250	453.86	200804	24h
TNFα	N5.14	DCMU	100	558.40	200804	24h
TNFα	N5.14	DCMU	10	740.22	200804	24h
TNFα	N5.14	DMSO	250	817.5	200804	24h
TNFα	N5.14	DMSO	100	833.40	200804	24h
TNFα	N5.14	DMSO	10	806.13	200804	24h
TNFα	N5.14	DCMU	250	453.86	200804	24h

TNFα	N5.14	DCMU	100	551.59	200804	24h
TNFα	N5.14	DCMU	10	740.22	200804	24h
GrB	N5.14	DMSO	250	32008.33	200916	D5
GrB	N5.14	DMSO	100	28091.66	200916	D5
GrB	N5.14	DMSO	10	50258.33	200916	D5
GrB	N5.14	DCMU	250	23258.33	200916	D5
GrB	N5.14	DCMU	100	17925	200916	D5
GrB	N5.14	DCMU	10	40091.66	200916	D5
GrB	N5.14	DMSO	250	30758.33	200916	D5
GrB	N5.14	DMSO	100	26675	200916	D5
GrB	N5.14	DMSO	10	47341.66	200916	D5
GrB	N5.14	DCMU	250	21008.33	200916	D5
GrB	N5.14	DCMU	100	16925	200916	D5
GrB	N5.14	DCMU	10	41008.33	200916	D5
GrB	N5.14	DMSO	250	3107.93	201105	24h
GrB	N5.14	DMSO	100	3996.82	201105	24h
GrB	N5.14	DMSO	10	3203.17	201105	24h
GrB	N5.14	DCMU	250	2726.98	201105	24h
GrB	N5.14	DCMU	100	2822.22	201105	24h
GrB	N5.14	DCMU	10	2663.49	201105	24h
GrB	N5.14	DMSO	250	2853.96	201105	24h
GrB	N5.14	DMSO	100	3266.66	201105	24h
GrB	N5.14	DMSO	10	2536.50	201105	24h
GrB	N5.14	DCMU	250	2790.47	201105	24h
GrB	N5.14	DCMU	100	3012.69	201105	24h
GrB	N5.14	DCMU	10	3076.19	201105	24h
GrB	N5.14	DMSO	250	3965.07	201105	D5
GrB	N5.14	DMSO	100	2377.77	201105	D5
GrB	N5.14	DMSO	10	3044.44	201105	D5
GrB	N5.14	DCMU	250	9076.19	201105	D5
GrB	N5.14	DCMU	100	3488.88	201105	D5
GrB	N5.14	DCMU	10	2695.23	201105	D5
GrB	N5.14	DMSO	250	4536.50	201105	D5
GrB	N5.14	DMSO	100	2441.26	201105	D5
GrB	N5.14	DMSO	10	3076.19	201105	D5
GrB	N5.14	DCMU	250	10155.55	201105	D5
GrB	N5.14	DCMU	100	3234.92	201105	D5
GrB	N5.14	DCMU	10	2695.23	201105	D5
GrB	N5.14	DMSO	250	8501.88	200629	24h
GrB	N5.14	DMSO	100	16350.94	200629	24h
GrB	N5.14	DMSO	10	19671.69	200629	24h
GrB	N5.14	DCMU	250	5369.81	200629	24h
GrB	N5.14	DCMU	100	14992.45	200629	24h
GrB	N5.14	DCMU	10	20162.26	200629	24h
GrB	N5.14	DMSO	250	10275.47	200629	24h
GrB	N5.14	DMSO	100	16992.45	200629	24h
GrB	N5.14	DMSO	10	20728.30	200629	24h
GrB	N5.14	DCMU	250	5860.37	200629	24h
GrB	N5.14	DCMU	100	19445.28	200629	24h
GrB	N5.14	DCMU	10	18615.09	200629	24h
GrB	N5.14	DMSO	250	10954.71	200629	24h
GrB	N5.14	DMSO	100	19860.37	200629	24h

GrB	N5.14	DMSO	10		16577.35	200629	24h
GrB	N5.14	DCMU	250		4501.88	200629	24h
GrB	N5.14	DCMU	100		14615.09	200629	24h
GrB	N5.14	DCMU	10		16954.71	200629	24h
GrB	N5.14	DMSO	250		11067.92	200629	24h
GrB	N5.14	DMSO	100		20426.41	200629	24h
GrB	N5.14	DMSO	10		18237.73	200629	24h
GrB	N5.14	DCMU	250		4879.24	200629	24h
GrB	N5.14	DCMU	100		15483.01	200629	24h
GrB	N5.14	DCMU	10		17747.16	200629	24h
GrB	N5.14	DMSO	250		12165.95	200803	D5
GrB	N5.14	DMSO	100		11400	200803	D5
GrB	N5.14	DMSO	10		2421.27	200803	D5
GrB	N5.14	DCMU	250		13442.55	200803	D5
GrB	N5.14	DCMU	100		18123.40	200803	D5
GrB	N5.14	DCMU	10		14974.46	200803	D5
GrB	N5.14	DMSO	250		13144.68	200803	D5
GrB	N5.14	DMSO	100		11272.34	200803	D5
GrB	N5.14	DMSO	10		1995.74	200803	D5
GrB	N5.14	DCMU	250		13868.08	200803	D5
GrB	N5.14	DCMU	100		16931.91	200803	D5
GrB	N5.14	DCMU	10		16506.38	200803	D5
IL2	N5.14	DMSO	250		2207.81	200626	24h
IL2	N5.14	DMSO	100		2176.56	200626	24h
IL2	N5.14	DMSO	10		2106.25	200626	24h
IL2	N5.14	DCMU	250		2075	200626	24h
IL2	N5.14	DCMU	100		2176.56	200626	24h
IL2	N5.14	DCMU	10		2090.62	200626	24h
IL2	N5.14	DMSO	250		2403.12	200626	24h
IL2	N5.14	DMSO	100		2356.25	200626	24h
IL2	N5.14	DMSO	10		2231.25	200626	24h
IL2	N5.14	DCMU	250		2168.75	200626	24h
IL2	N5.14	DCMU	100		2348.43	200626	24h
IL2	N5.14	DCMU	10		2184.37	200626	24h
IL2	N5.14	DMSO	250		2317.18	200626	24h
IL2	N5.14	DMSO	100		2168.75	200626	24h
IL2	N5.14	DMSO	10		2215.62	200626	24h
IL2	N5.14	DCMU	250		2215.62	200626	24h
IL2	N5.14	DCMU	100		2301.56	200626	24h
IL2	N5.14	DCMU	10		2309.37	200626	24h
IL2	N5.14	DMSO	250		2395.31	200626	24h
IL2	N5.14	DMSO	100		2254.68	200626	24h
IL2	N5.14	DMSO	10		2293.75	200626	24h
IL2	N5.14	DCMU	250		2192.18	200626	24h
IL2	N5.14	DCMU	100		2426.56	200626	24h
IL2	N5.14	DCMU	10		2340.62	200626	24h
IL2	N5.14	DMSO	250		6569.51	200805	D5
IL2	N5.14	DMSO	100		6435.36	200805	D5
IL2	N5.14	DMSO	10		6862.19	200805	D5
IL2	N5.14	DCMU	250		6654.87	200805	D5
IL2	N5.14	DCMU	100		6276.82	200805	D5
IL2	N5.14	DCMU	10		6667.07	200805	D5

IL2	N5.14	DMSO	250	6776.82	200805	D5
IL2	N5.14	DMSO	100	6691.46	200805	D5
IL2	N5.14	DMSO	10	6874.39	200805	D5
IL2	N5.14	DCMU	250	6947.56	200805	D5
IL2	N5.14	DCMU	100	6447.56	200805	D5
IL2	N5.14	DCMU	10	6593.90	200805	D5
IL2	N5.14	DMSO	250	6618.29	200805	D5
IL2	N5.14	DMSO	100	6618.29	200805	D5
IL2	N5.14	DMSO	10	6606.09	200805	D5
IL2	N5.14	DCMU	250	6545.12	200805	D5
IL2	N5.14	DCMU	100	6240.24	200805	D5
IL2	N5.14	DCMU	10	6593.90	200805	D5
IL2	N5.14	DMSO	250	6813.41	200805	D5
IL2	N5.14	DMSO	100	6740.24	200805	D5
IL2	N5.14	DMSO	10	6618.29	200805	D5
IL2	N5.14	DCMU	250	6569.51	200805	D5
IL2	N5.14	DCMU	100	6252.43	200805	D5
IL2	N5.14	DCMU	10	6593.90	200805	D5
IL2	N5.14	DMSO	250	7325	200615	24h
IL2	N5.14	DMSO	100	7658.33	200615	24h
IL2	N5.14	DMSO	10	7225	200615	24h
IL2	N5.14	DCMU	250	7325	200615	24h
IL2	N5.14	DCMU	100	6758.33	200615	24h
IL2	N5.14	DCMU	10	6891.66	200615	24h
IL2	N5.14	DMSO	250	7208.33	200615	24h
IL2	N5.14	DMSO	100	7925	200615	24h
IL2	N5.14	DMSO	10	7791.66	200615	24h
IL2	N5.14	DCMU	250	7458.33	200615	24h
IL2	N5.14	DCMU	100	7291.66	200615	24h
IL2	N5.14	DCMU	10	6875	200615	24h
IL2	N5.14	DMSO	250	6541.66	200615	24h
IL2	N5.14	DMSO	100	7175	200615	24h
IL2	N5.14	DMSO	10	7125	200615	24h
IL2	N5.14	DCMU	250	7291.66	200615	24h
IL2	N5.14	DCMU	100	6741.66	200615	24h
IL2	N5.14	DCMU	10	7091.66	200615	24h
